# Supplementary figures and images for: Bone regeneration in Ds-Red pig calvarial defect using allogenic transplantation of EGFP-pMSCs – A comparison of host cells and seeding cells in the scaffold (part 1 of 2)
Source: PLoS One. 2019 Jul 18;14(7):e0215499. doi: 10.1371/journal.pone.0215499 (PMC6638893; doi:10.1371/journal.pone.0215499)

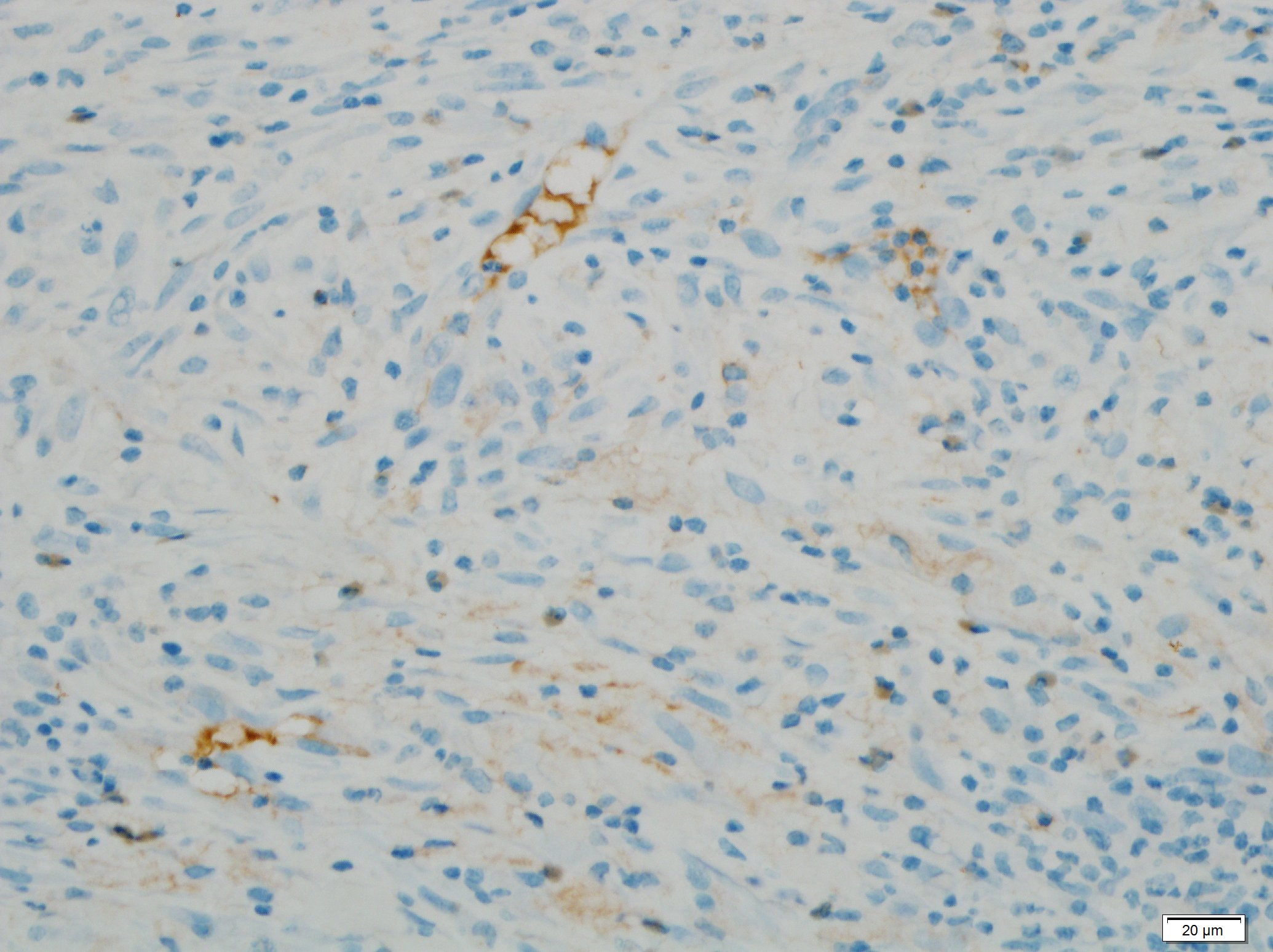

Supplement: S1 File — (ZIP) [file pone.0215499.s001.zip › CD68 and IHC stain/1 week/2-5 40x-1.jpg]

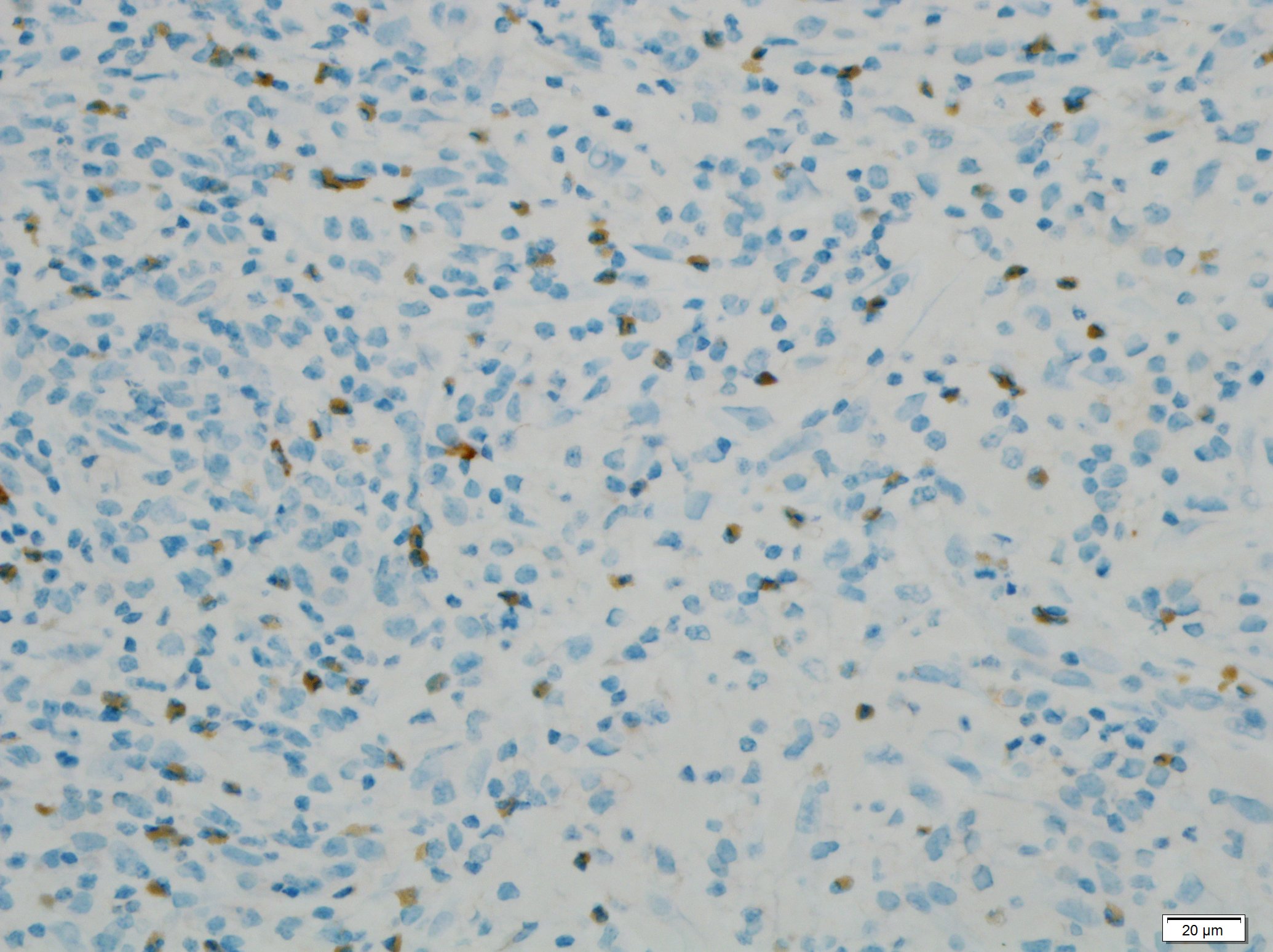

Supplement: S1 File — (ZIP) [file pone.0215499.s001.zip › CD68 and IHC stain/1 week/2-5 40x-2.jpg]

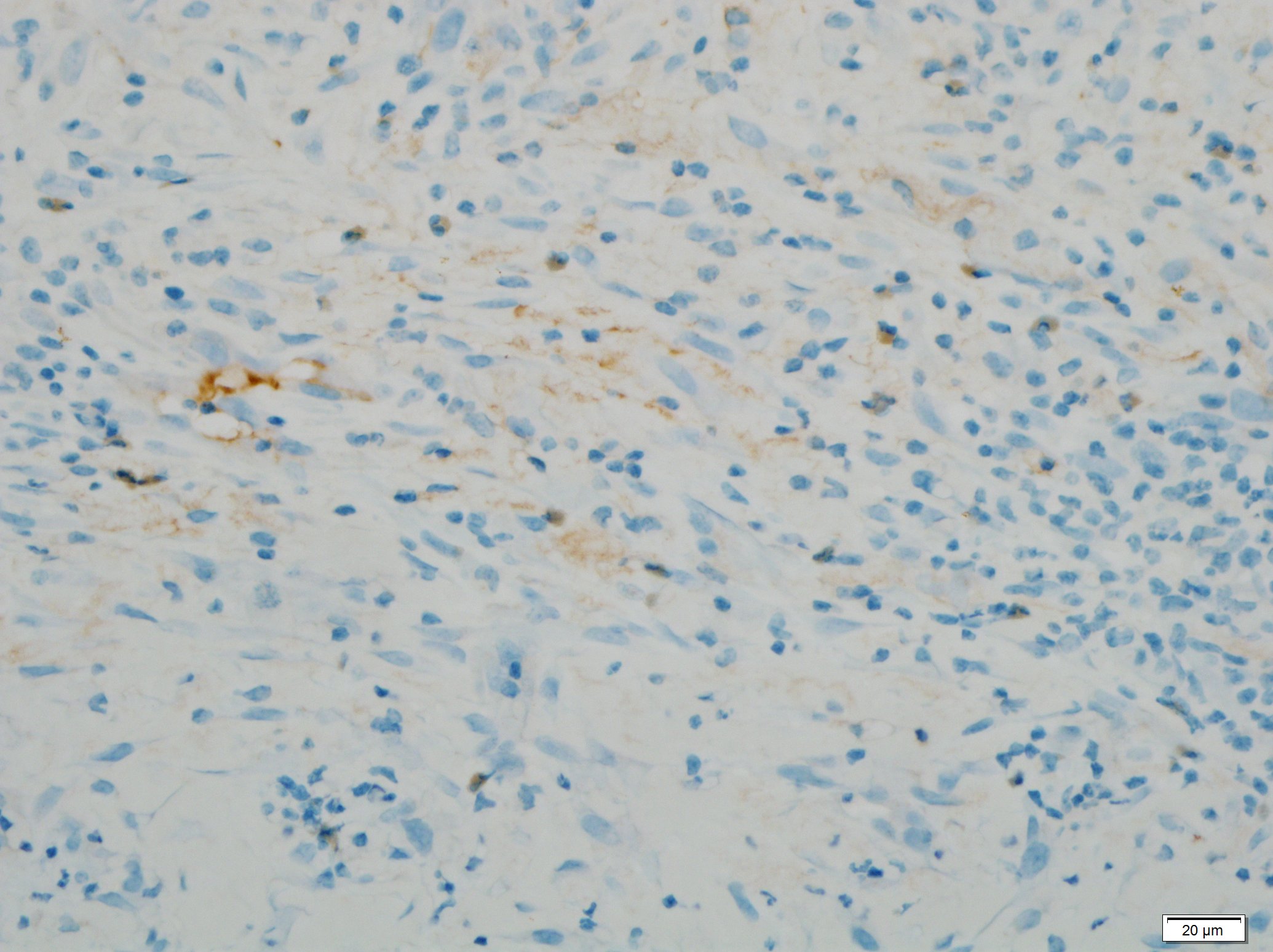

Supplement: S1 File — (ZIP) [file pone.0215499.s001.zip › CD68 and IHC stain/1 week/2-5 40x-3.jpg]

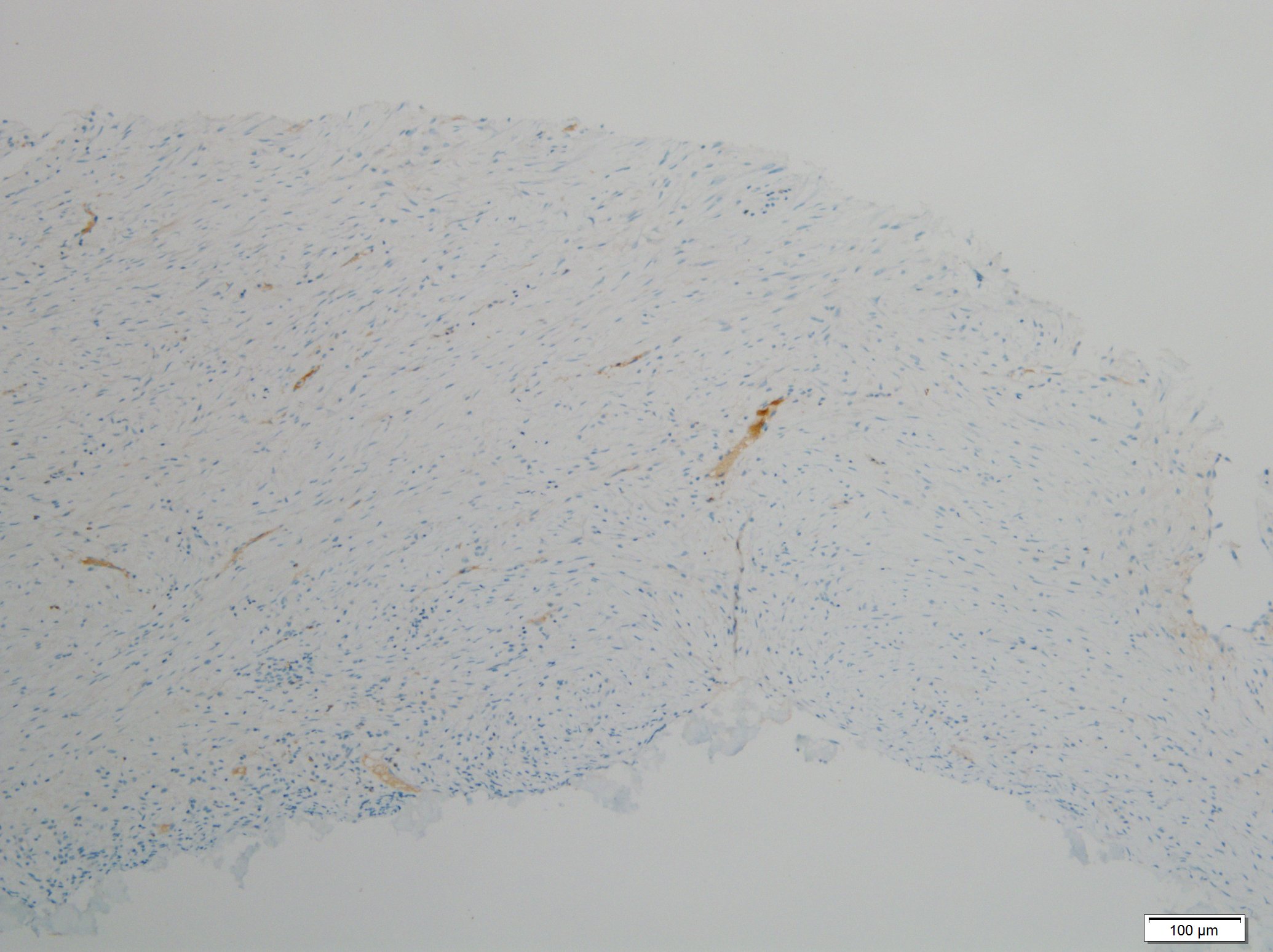

Supplement: S1 File — (ZIP) [file pone.0215499.s001.zip › CD68 and IHC stain/1 week/2-6 10x-1.jpg]

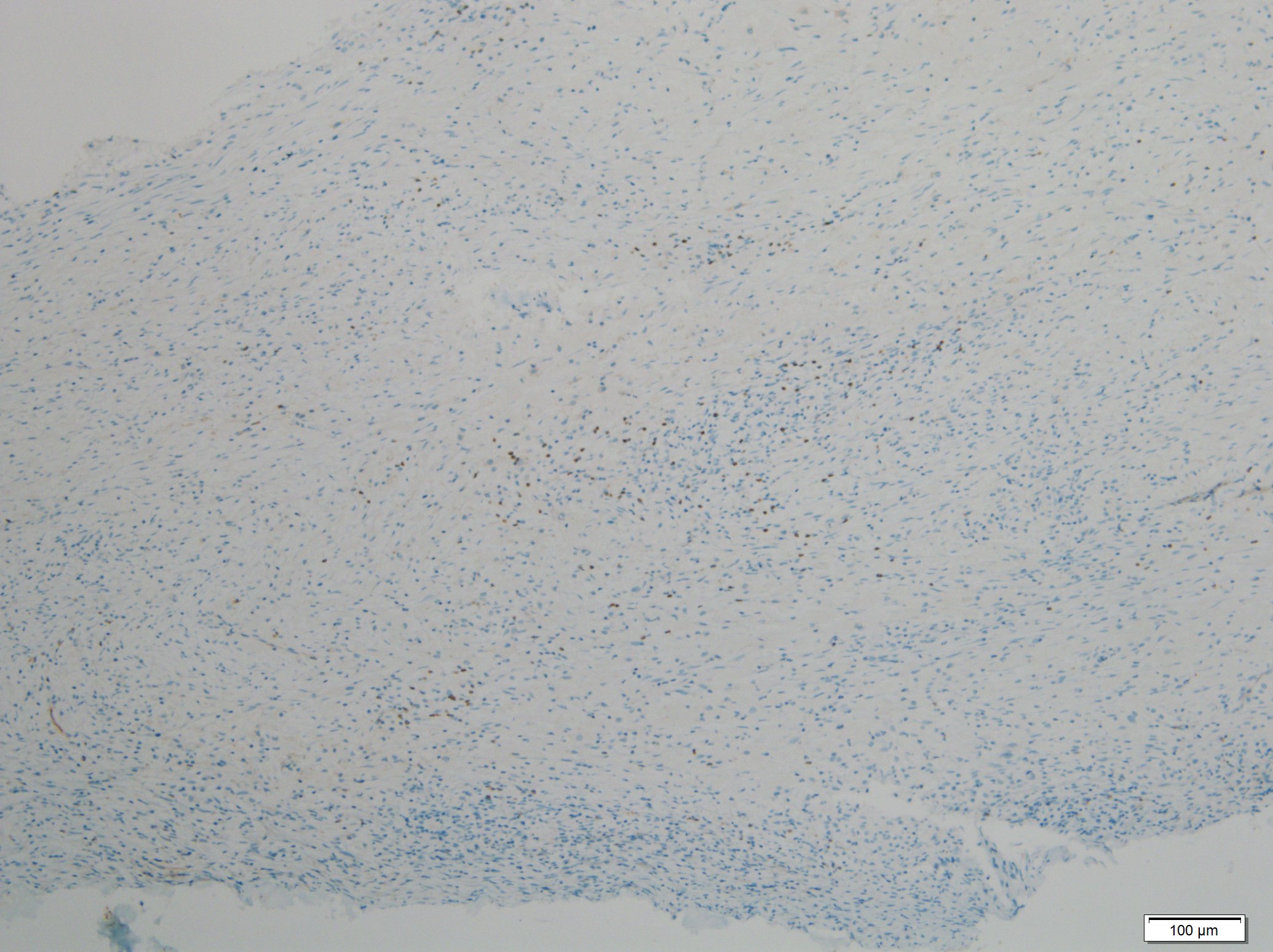

Supplement: S1 File — (ZIP) [file pone.0215499.s001.zip › CD68 and IHC stain/1 week/2-6 10x-2.jpg]

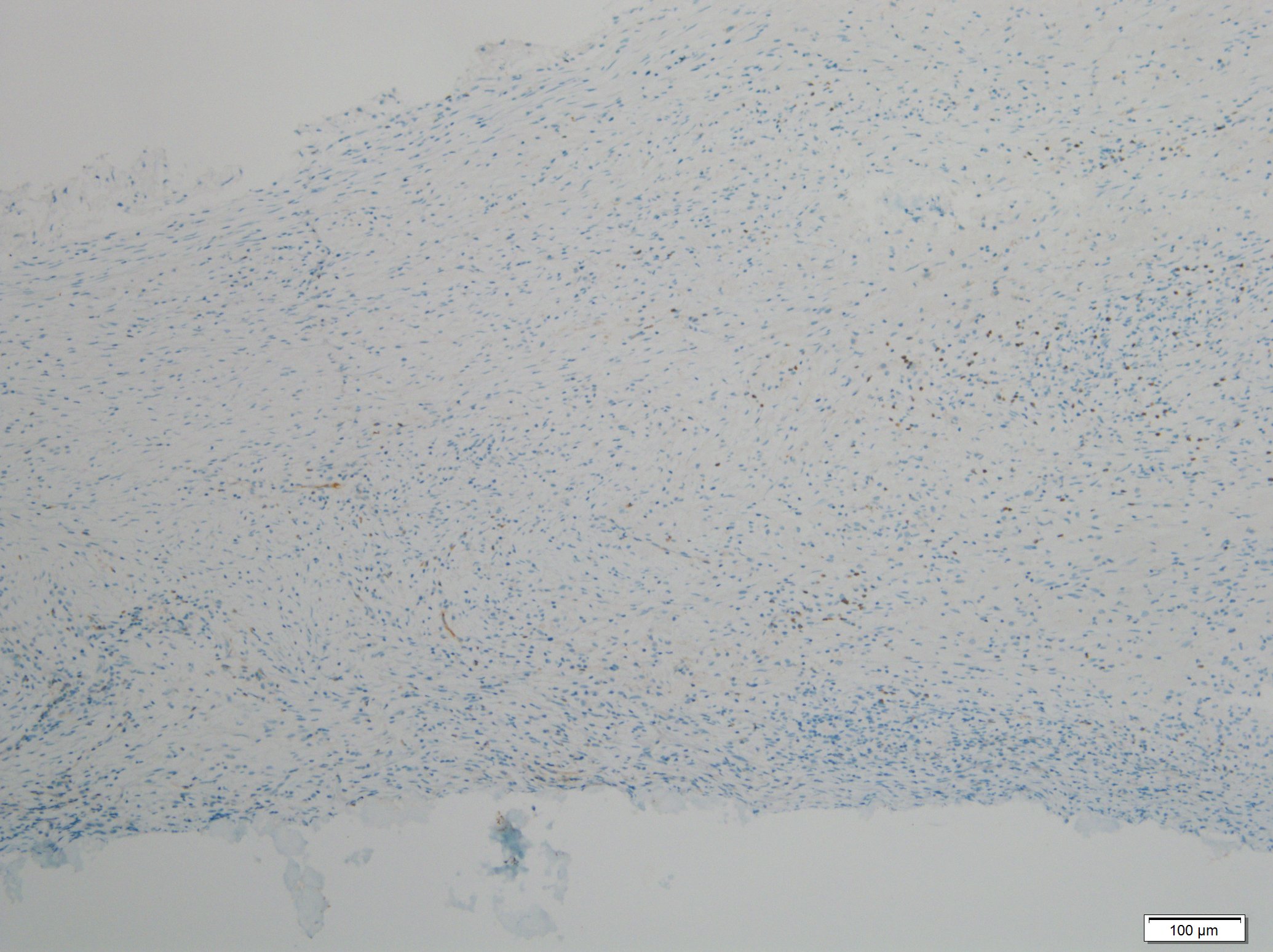

Supplement: S1 File — (ZIP) [file pone.0215499.s001.zip › CD68 and IHC stain/1 week/2-6 10x-3.jpg]

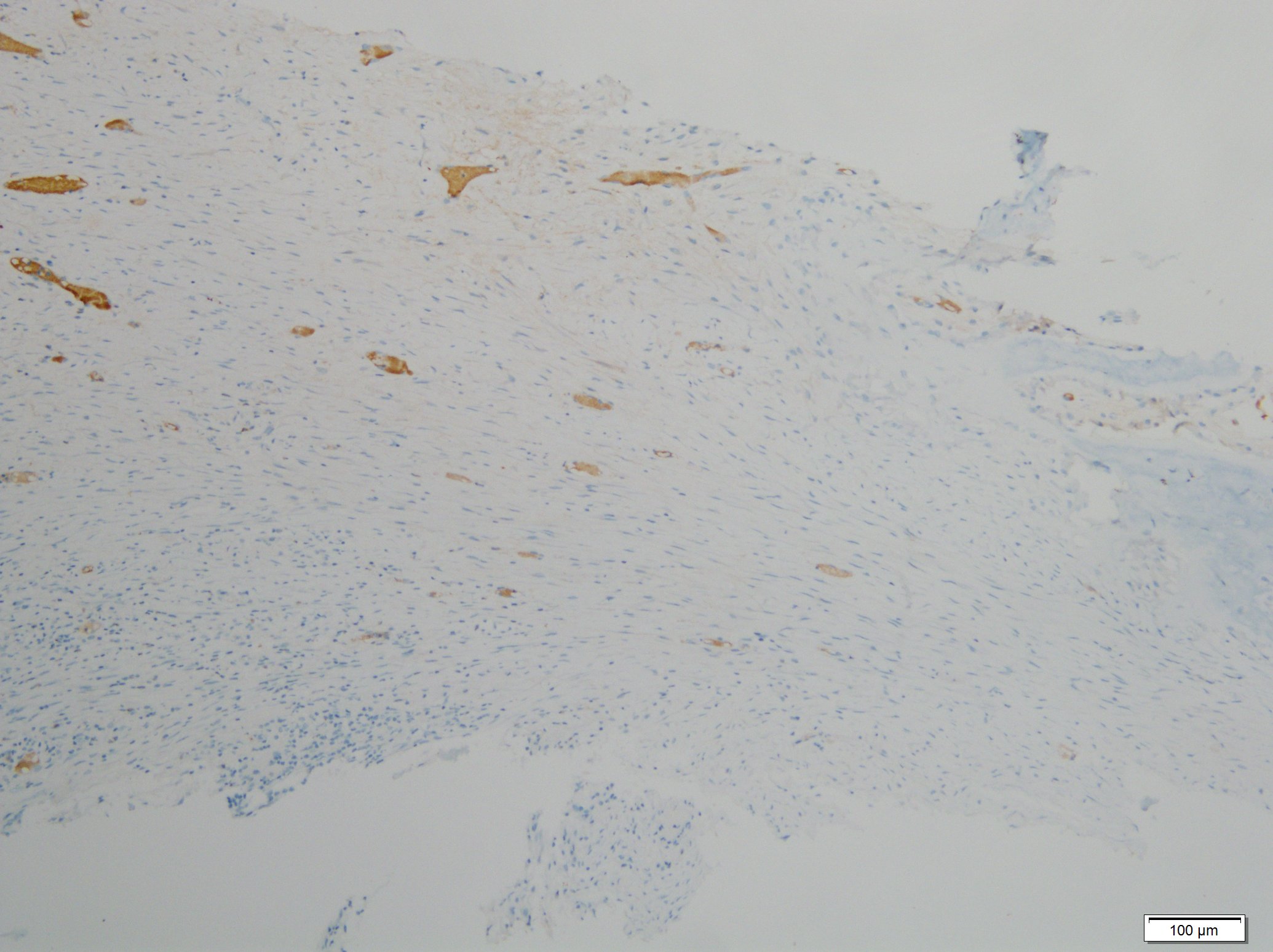

Supplement: S1 File — (ZIP) [file pone.0215499.s001.zip › CD68 and IHC stain/1 week/2-7 10x-1.jpg]

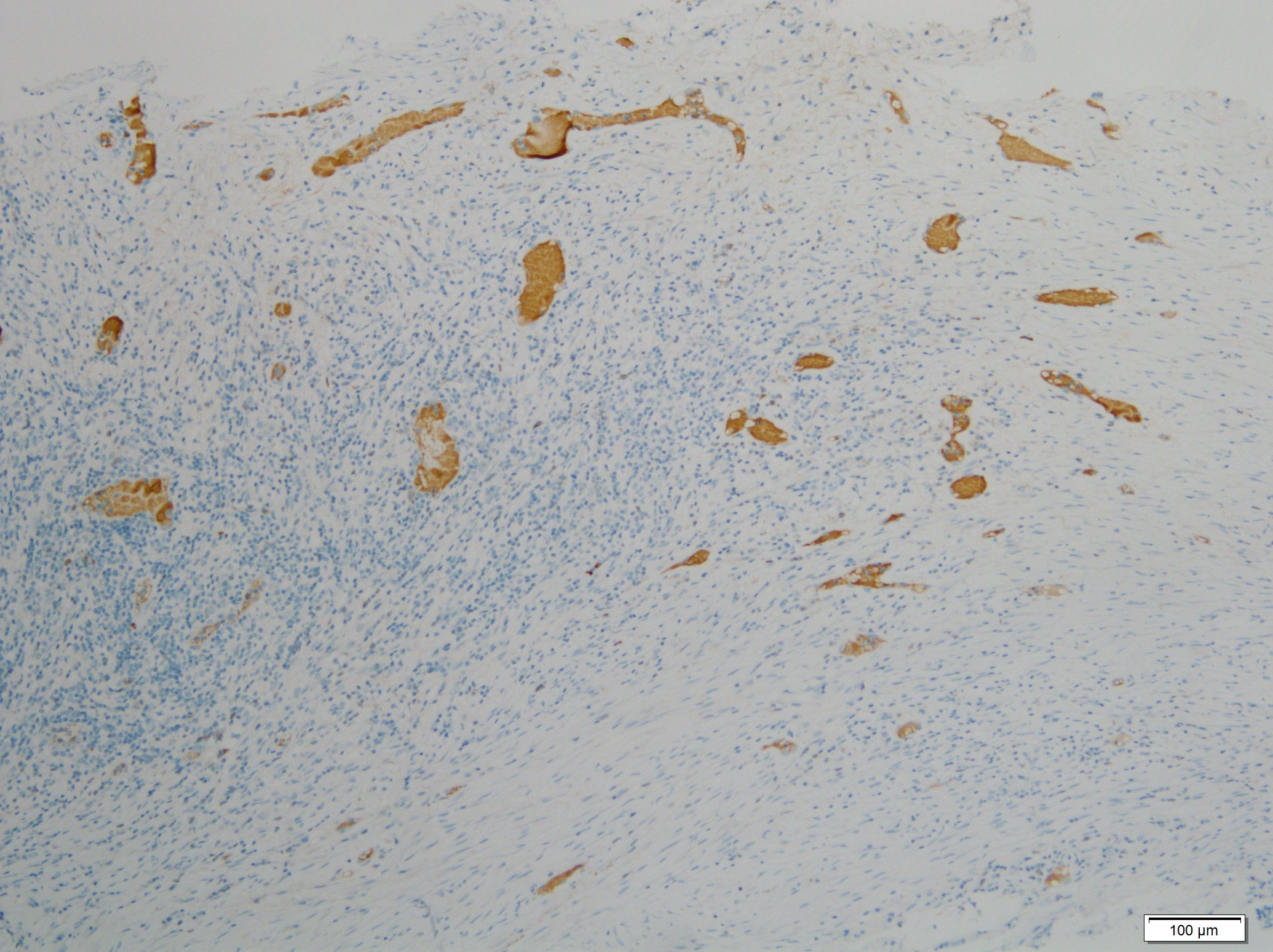

Supplement: S1 File — (ZIP) [file pone.0215499.s001.zip › CD68 and IHC stain/1 week/2-7 10x-2.jpg]

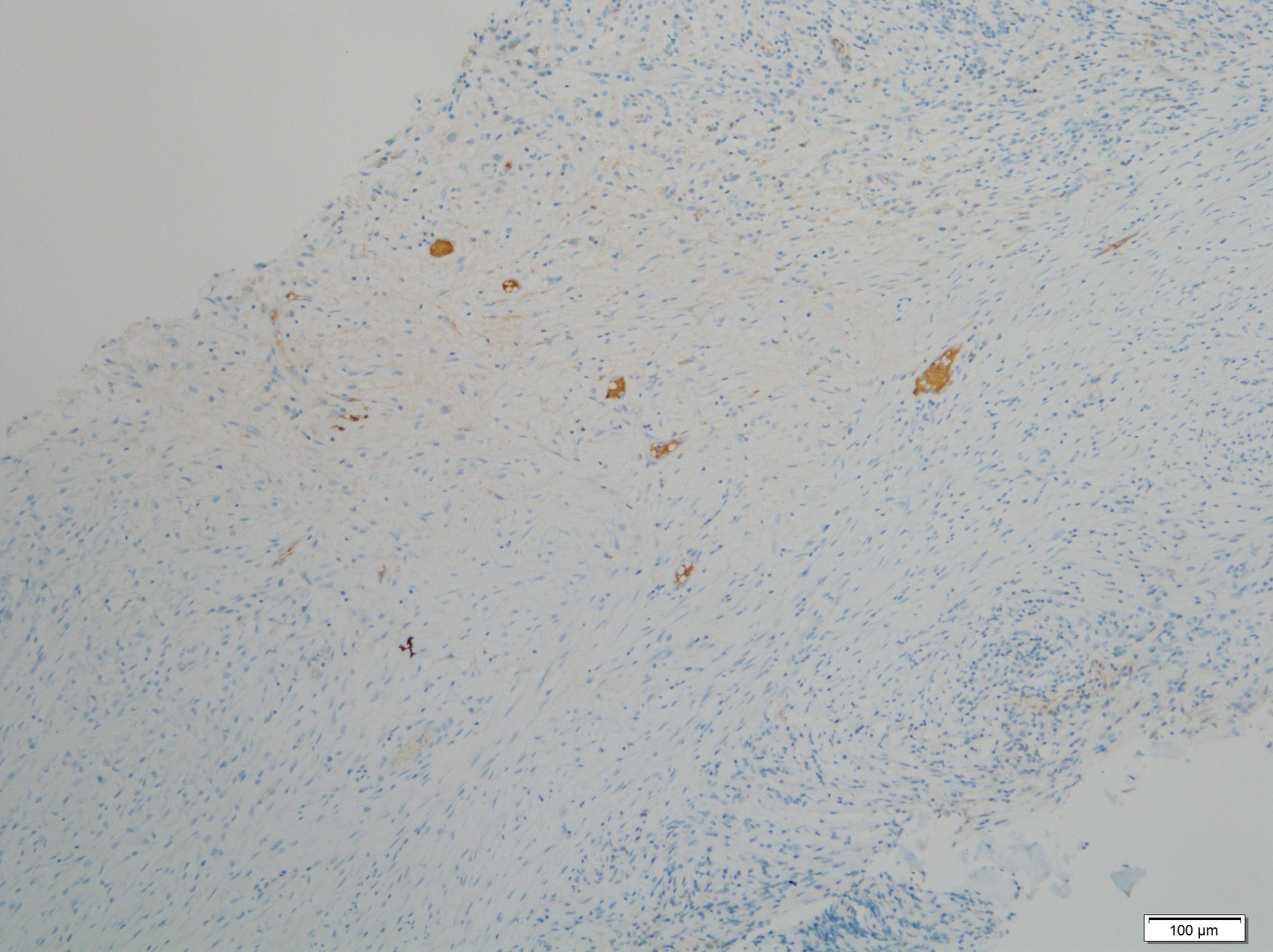

Supplement: S1 File — (ZIP) [file pone.0215499.s001.zip › CD68 and IHC stain/1 week/2-7 10x-3.jpg]

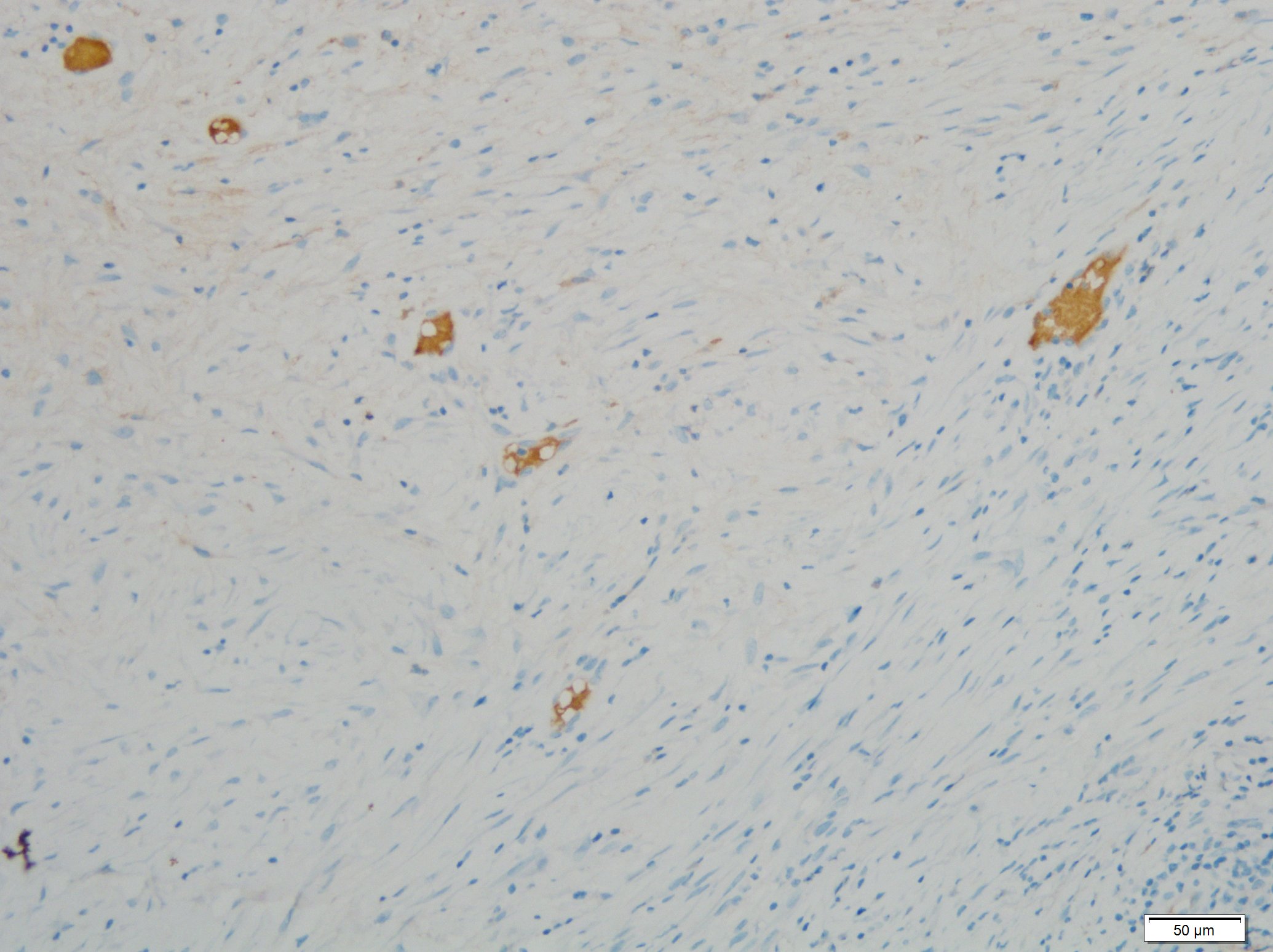

Supplement: S1 File — (ZIP) [file pone.0215499.s001.zip › CD68 and IHC stain/1 week/2-7 20x-1.jpg]

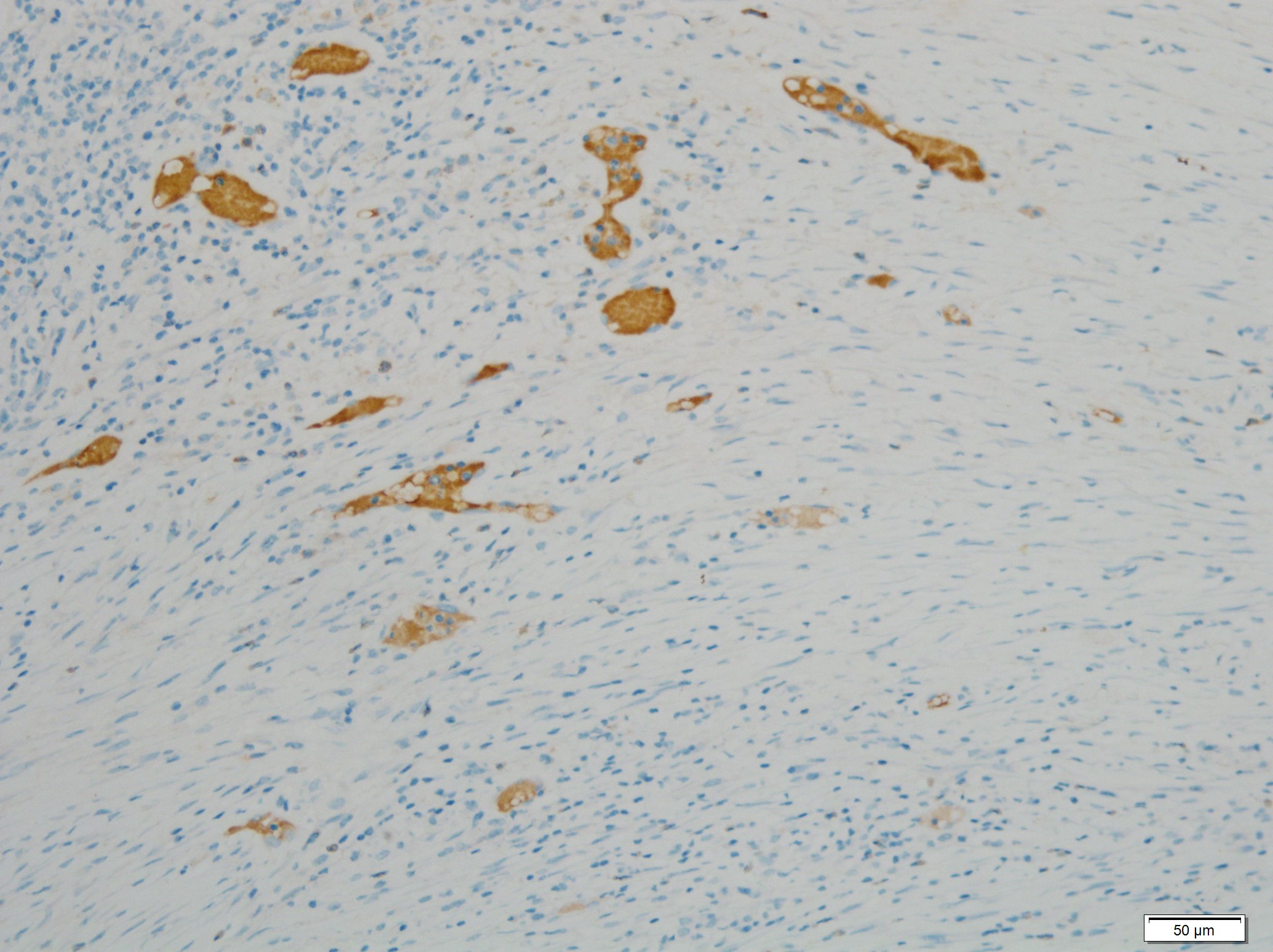

Supplement: S1 File — (ZIP) [file pone.0215499.s001.zip › CD68 and IHC stain/1 week/2-7 20x-2.jpg]

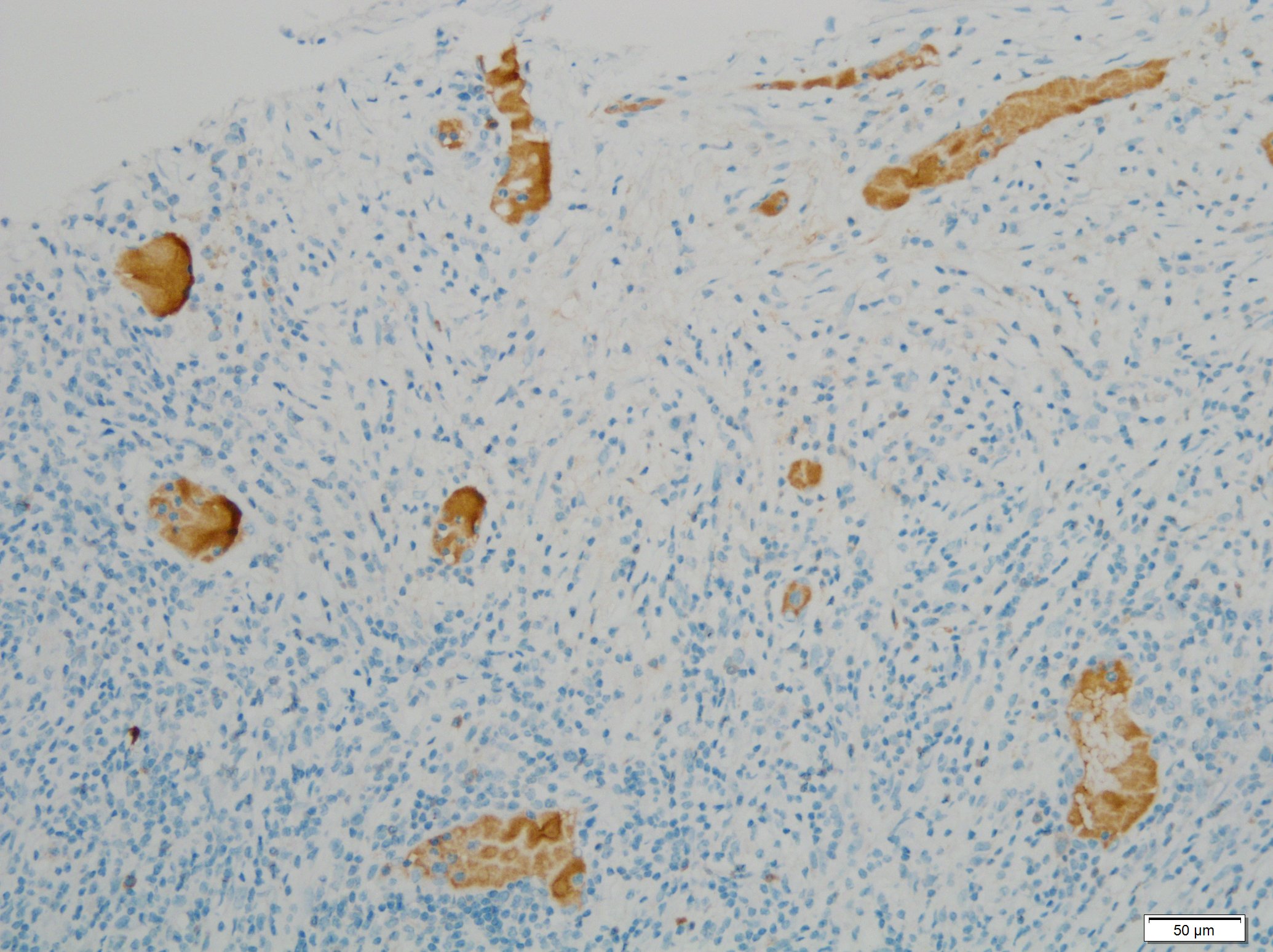

Supplement: S1 File — (ZIP) [file pone.0215499.s001.zip › CD68 and IHC stain/1 week/2-7 20x-3.jpg]

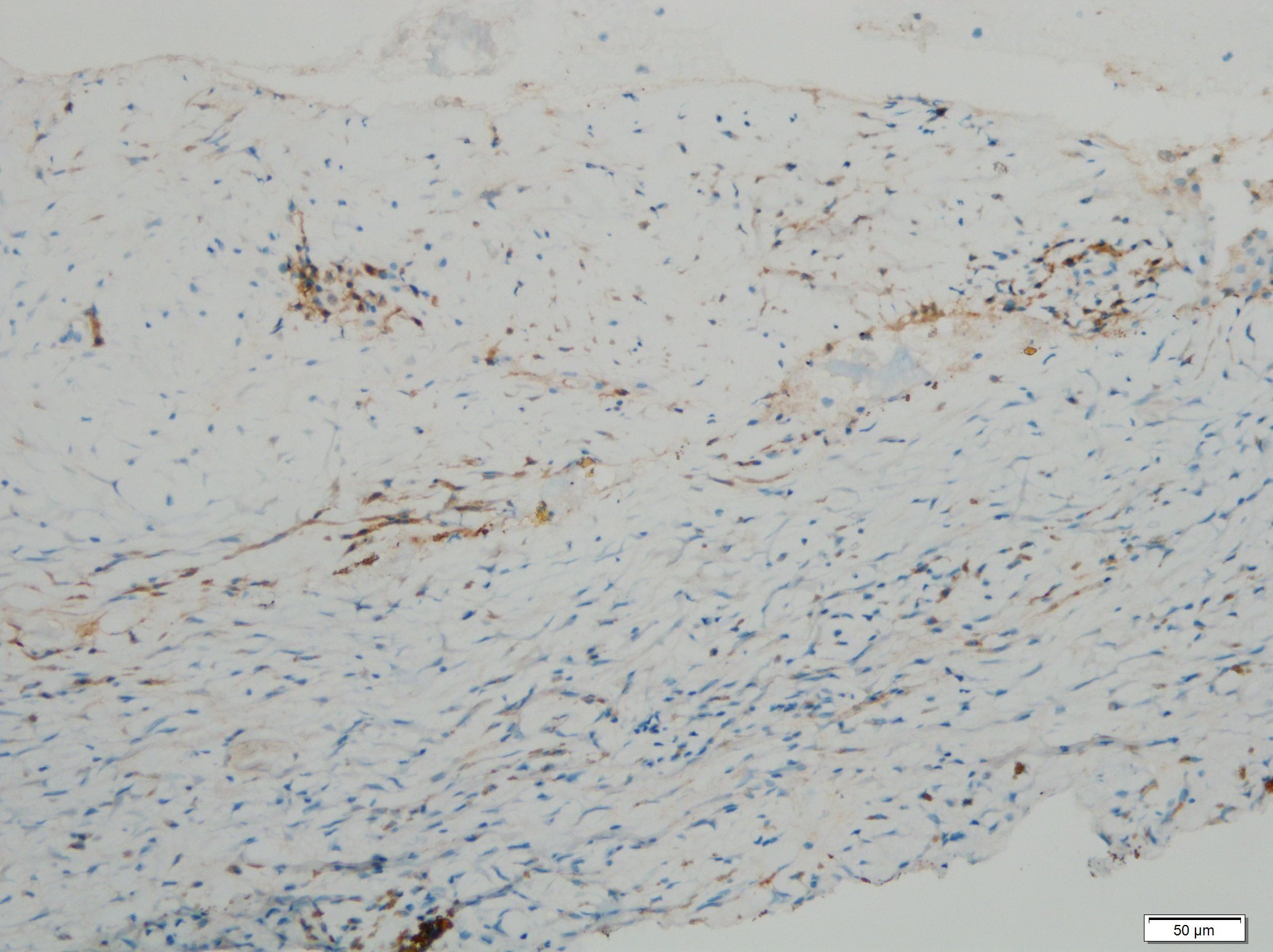

Supplement: S1 File — (ZIP) [file pone.0215499.s001.zip › CD68 and IHC stain/2 weeks/1-6 20x-1.jpg]

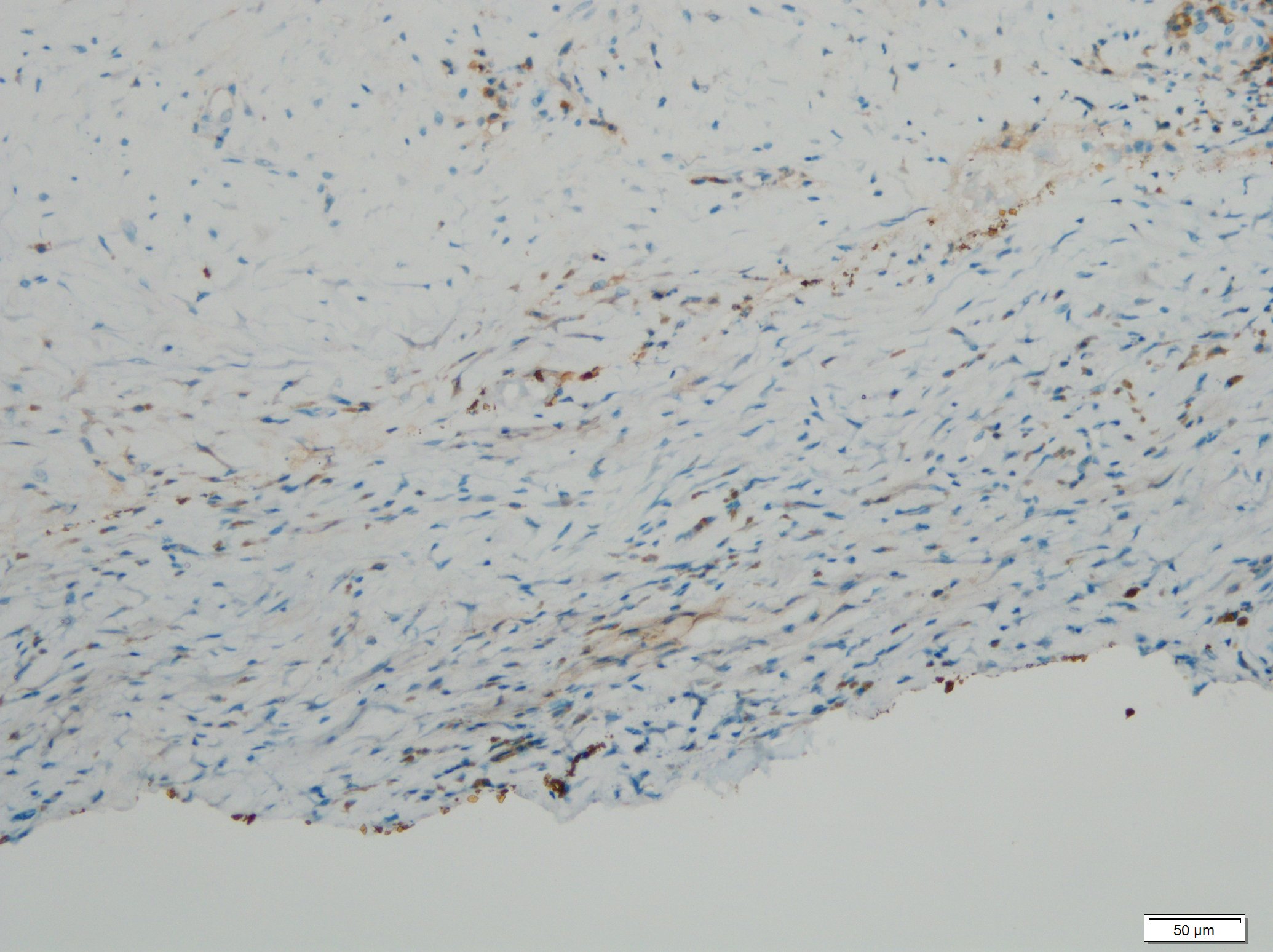

Supplement: S1 File — (ZIP) [file pone.0215499.s001.zip › CD68 and IHC stain/2 weeks/1-6 20x-2.jpg]

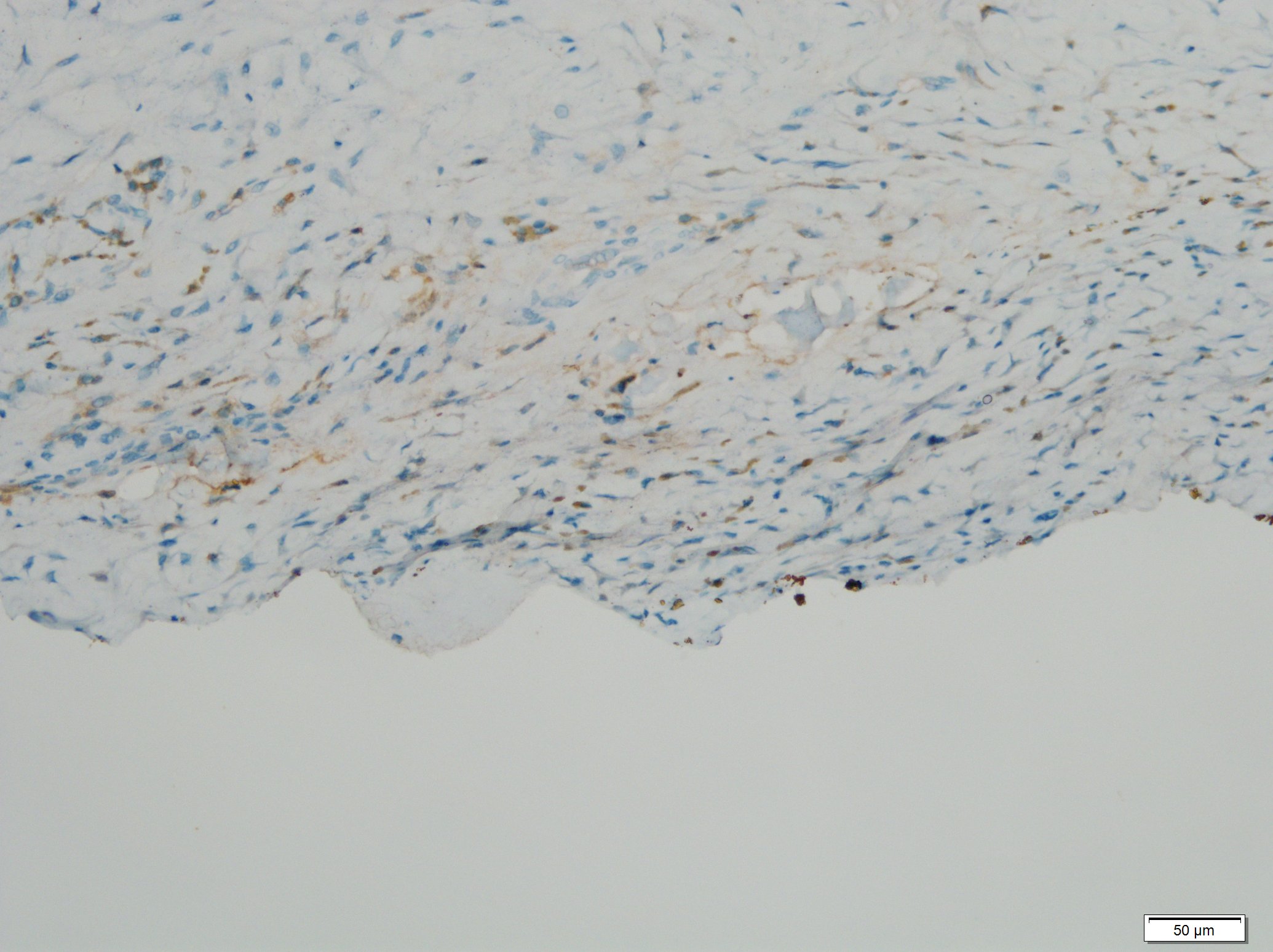

Supplement: S1 File — (ZIP) [file pone.0215499.s001.zip › CD68 and IHC stain/2 weeks/1-6 20x-3.jpg]

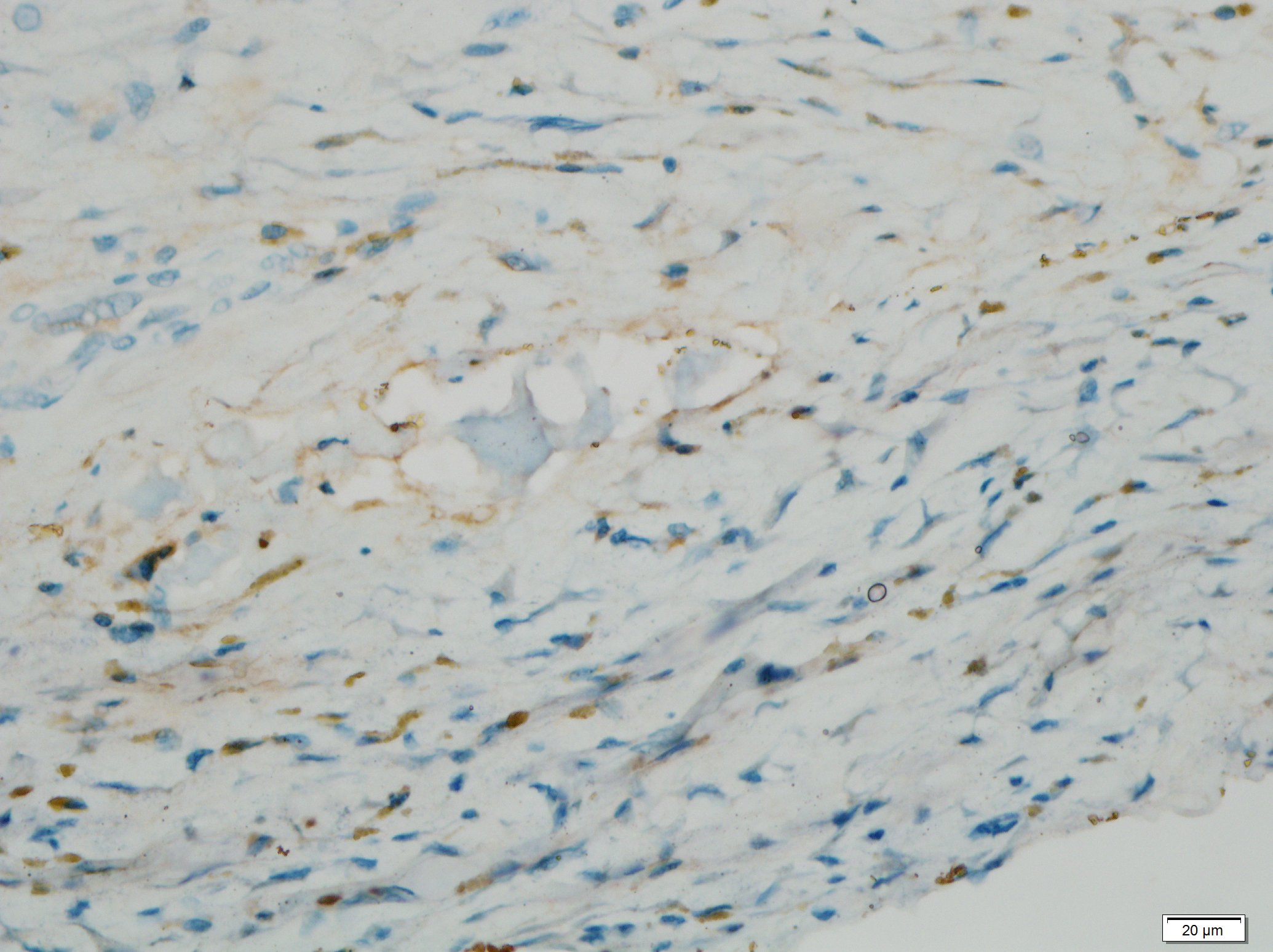

Supplement: S1 File — (ZIP) [file pone.0215499.s001.zip › CD68 and IHC stain/2 weeks/1-6 40x-1.jpg]

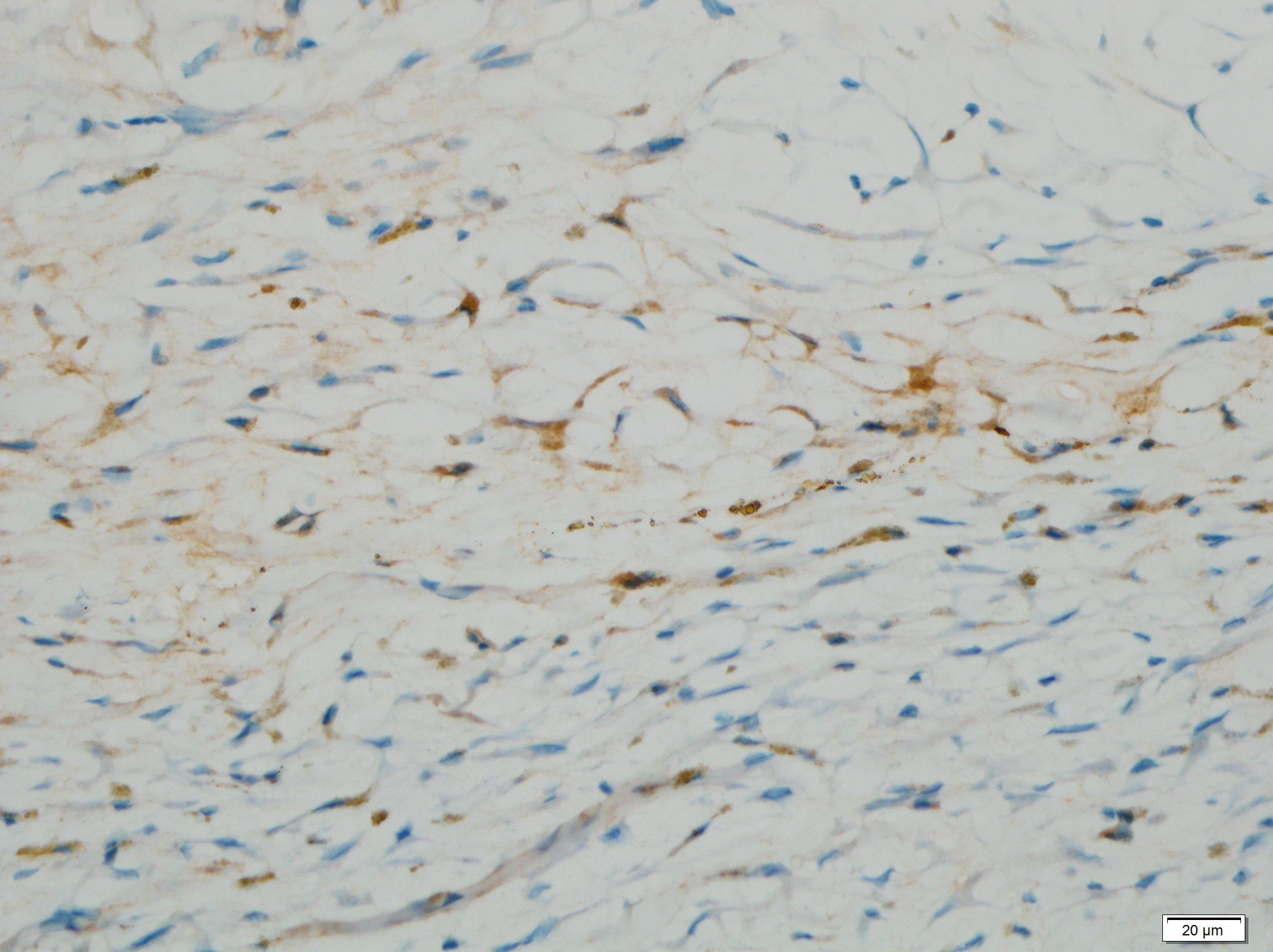

Supplement: S1 File — (ZIP) [file pone.0215499.s001.zip › CD68 and IHC stain/2 weeks/1-6 40x-2.jpg]

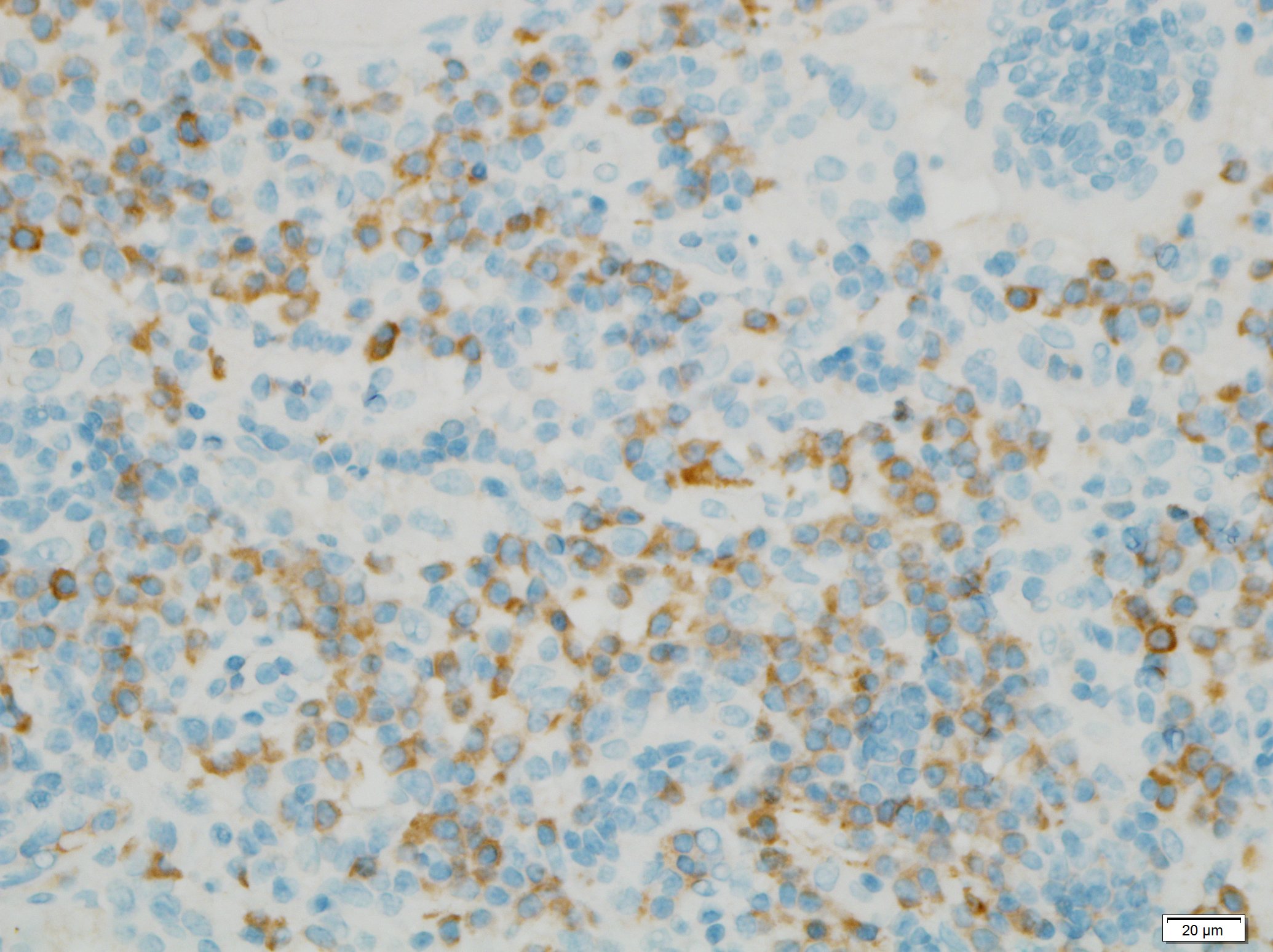

Supplement: S1 File — (ZIP) [file pone.0215499.s001.zip › CD68 and IHC stain/2 weeks/1-7 40x-1.jpg]

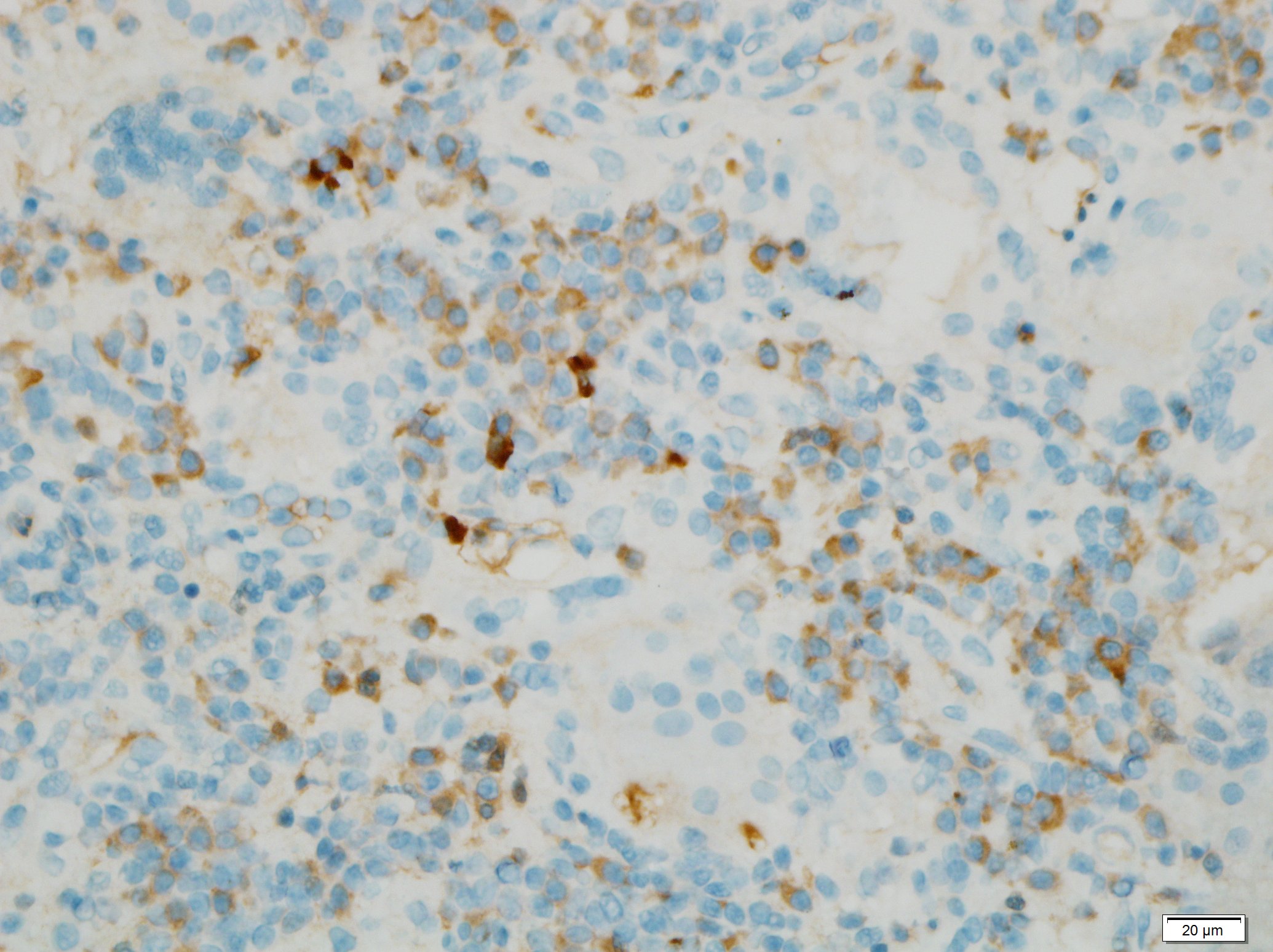

Supplement: S1 File — (ZIP) [file pone.0215499.s001.zip › CD68 and IHC stain/2 weeks/1-7 40x-2.jpg]

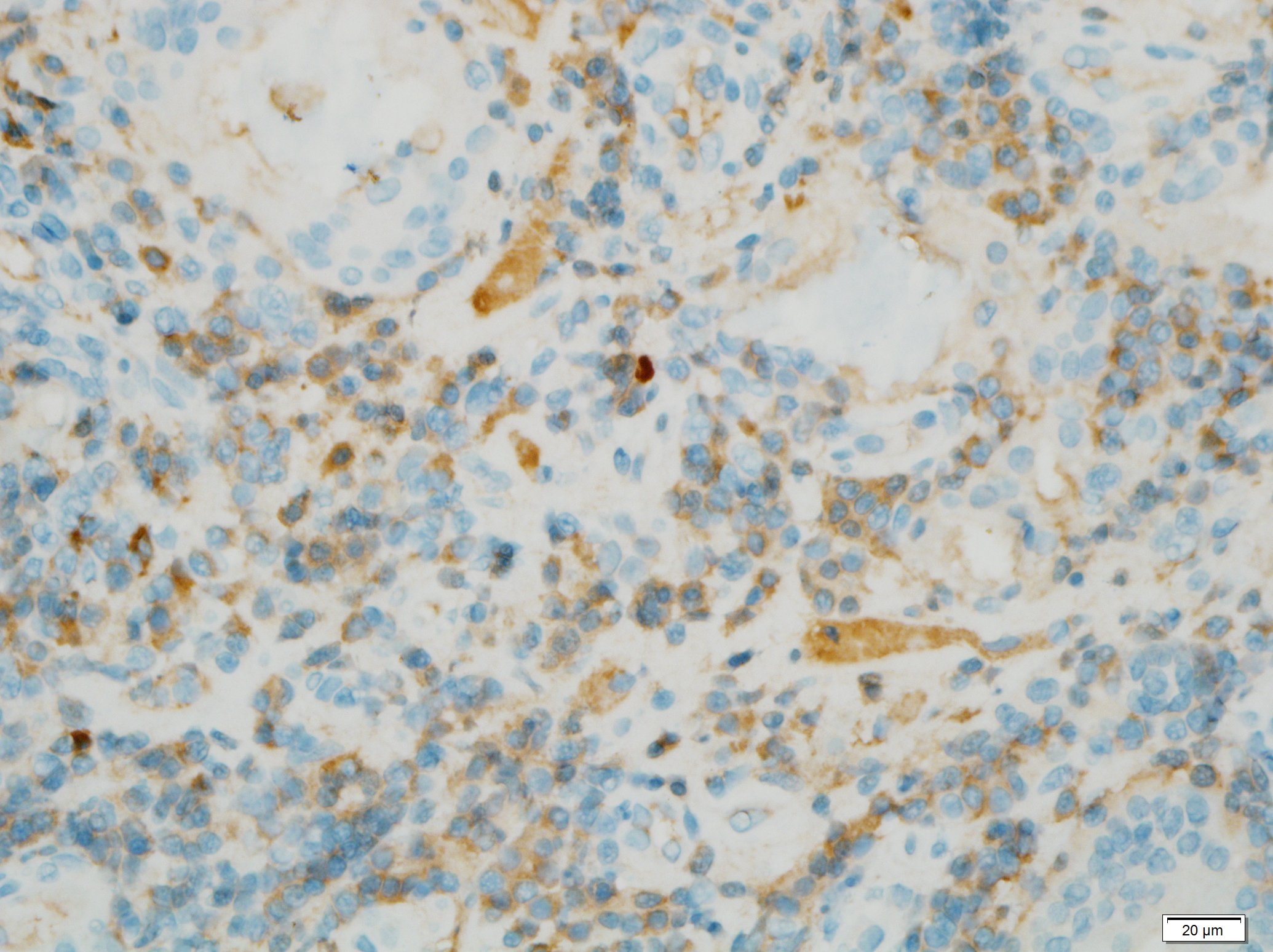

Supplement: S1 File — (ZIP) [file pone.0215499.s001.zip › CD68 and IHC stain/2 weeks/1-7 40x-3.jpg]

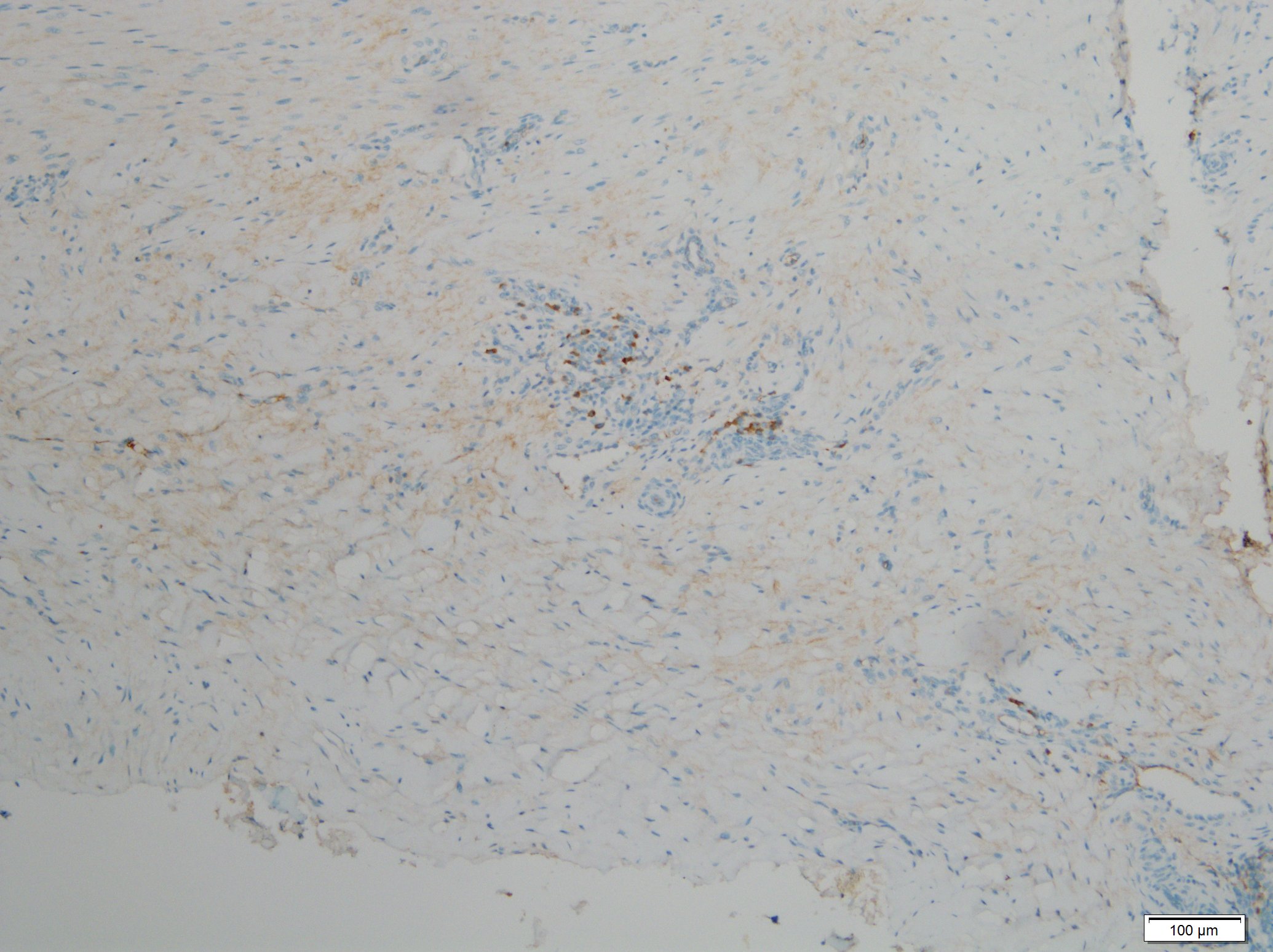

Supplement: S1 File — (ZIP) [file pone.0215499.s001.zip › CD68 and IHC stain/3 weeks/No. 3/3-1 10x-1.jpg]

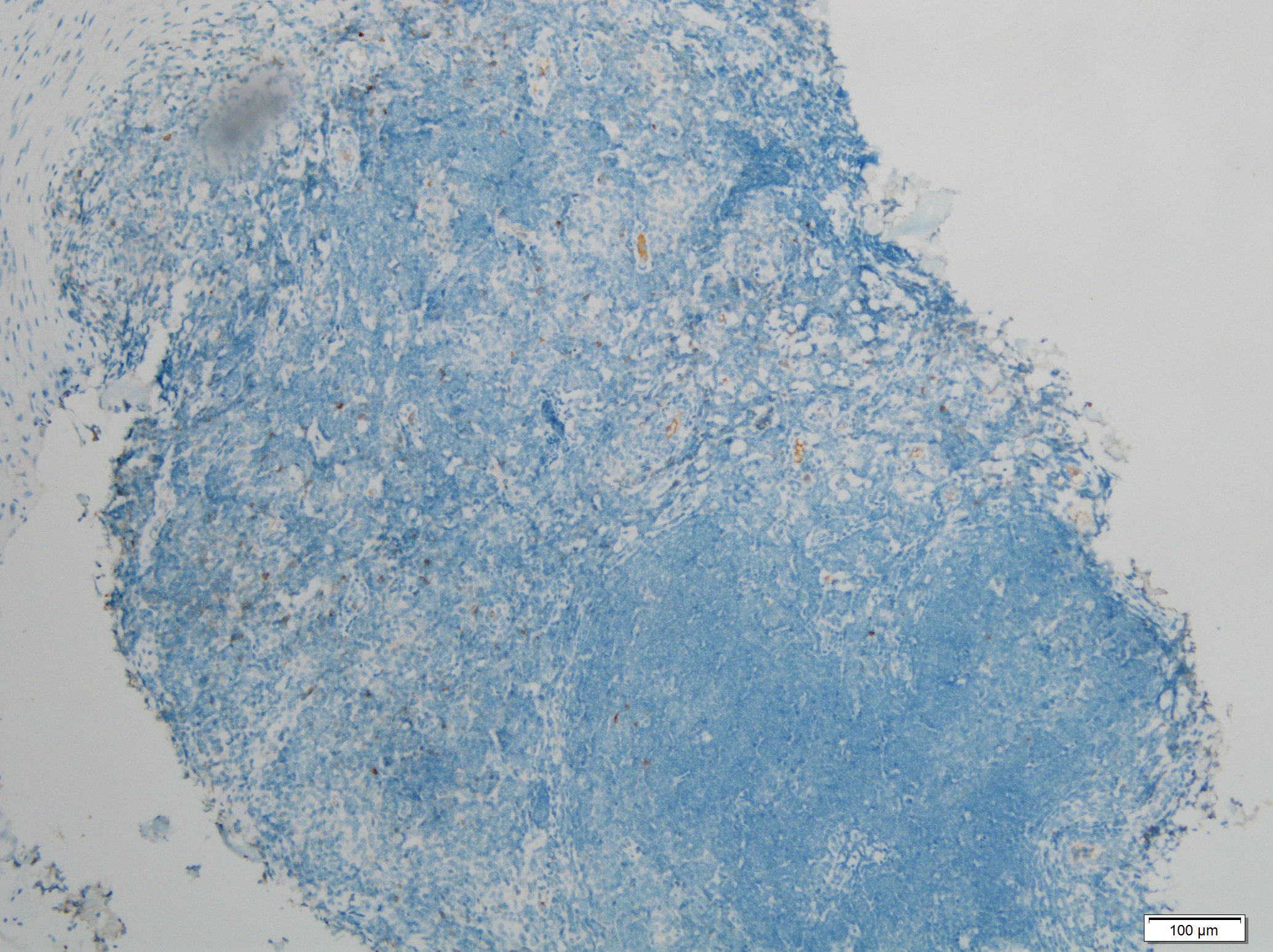

Supplement: S1 File — (ZIP) [file pone.0215499.s001.zip › CD68 and IHC stain/3 weeks/No. 3/3-1 10x-2.jpg]

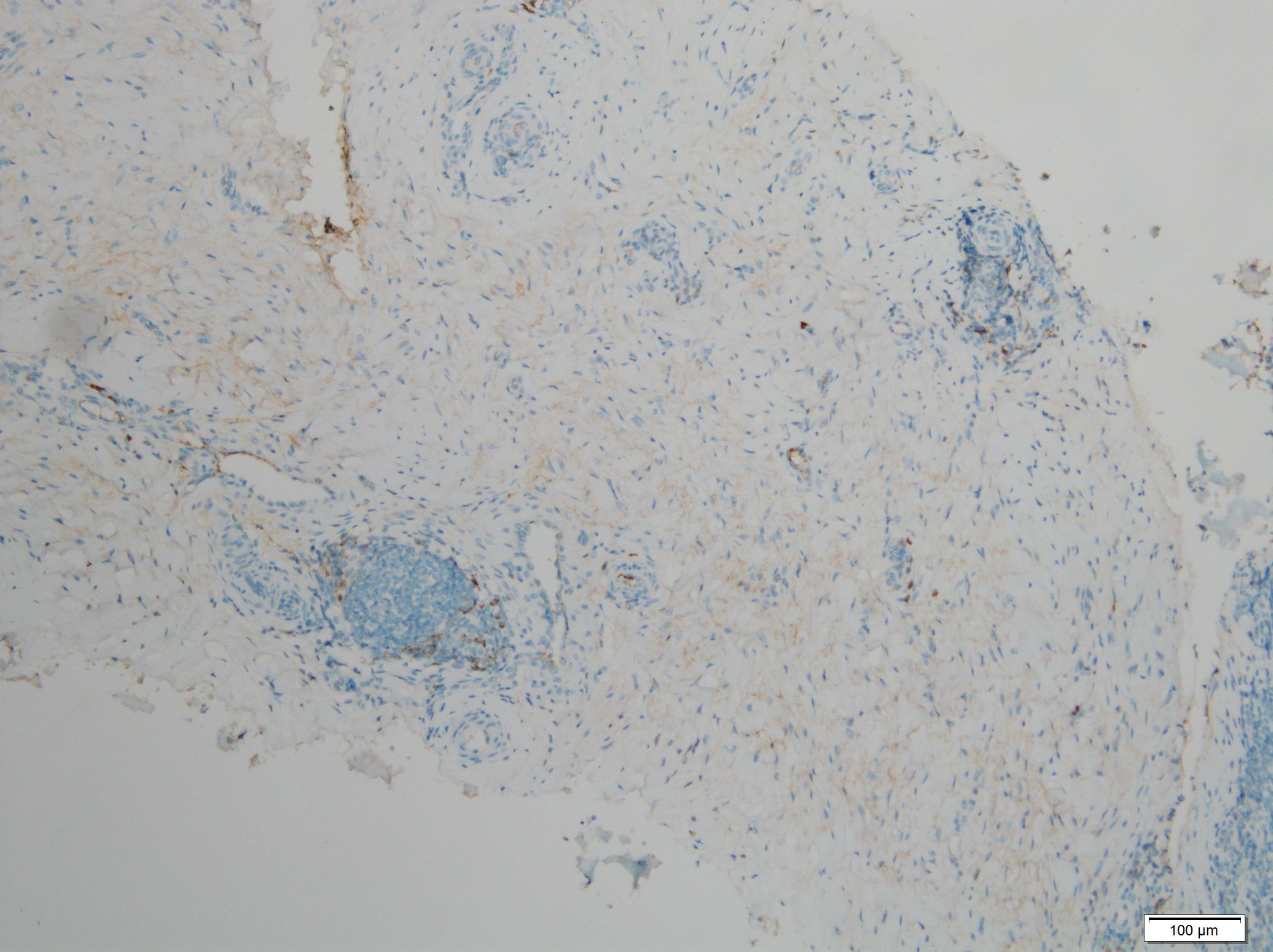

Supplement: S1 File — (ZIP) [file pone.0215499.s001.zip › CD68 and IHC stain/3 weeks/No. 3/3-1 10x-3.jpg]

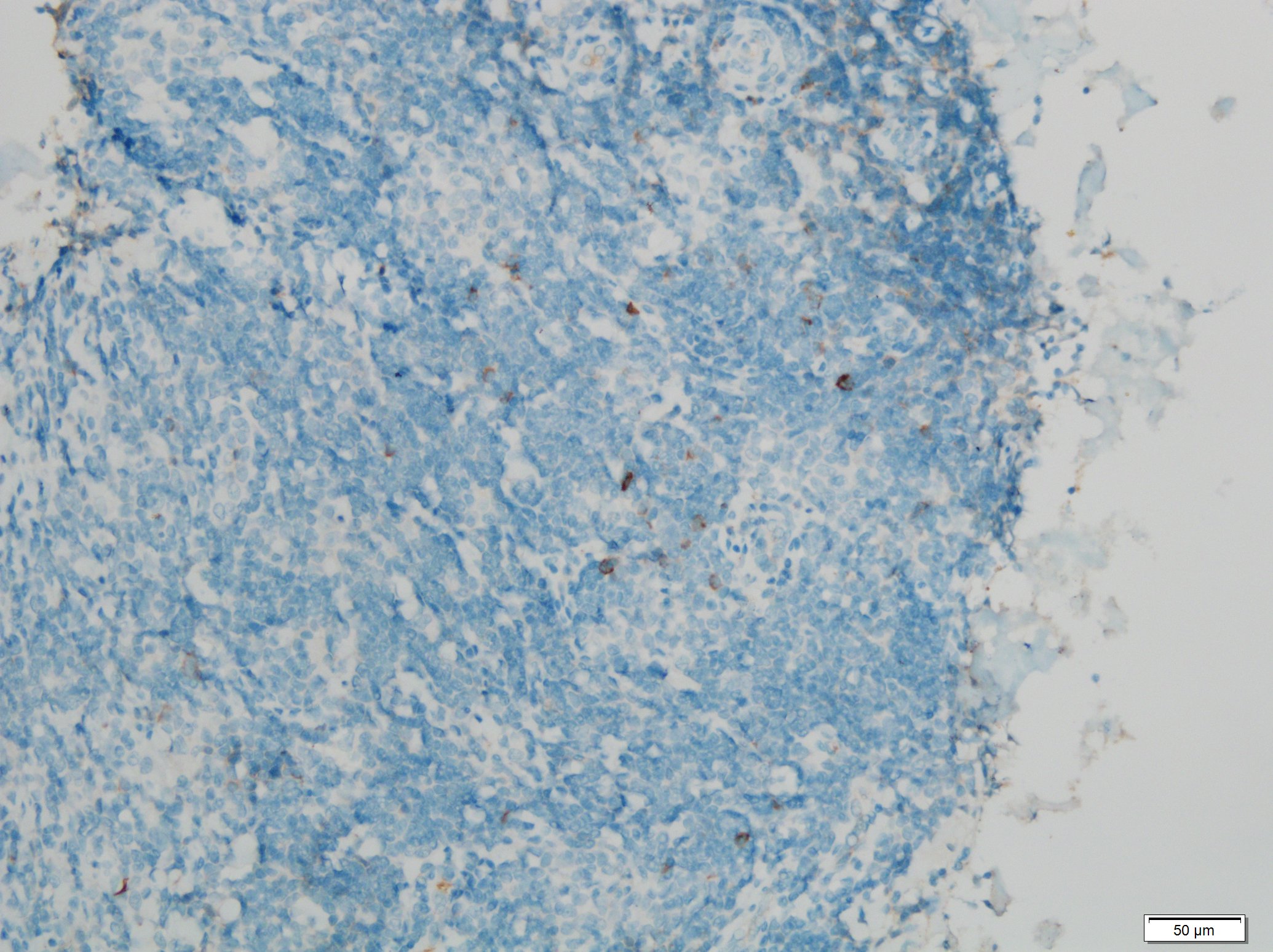

Supplement: S1 File — (ZIP) [file pone.0215499.s001.zip › CD68 and IHC stain/3 weeks/No. 3/3-1 20x-1.jpg]

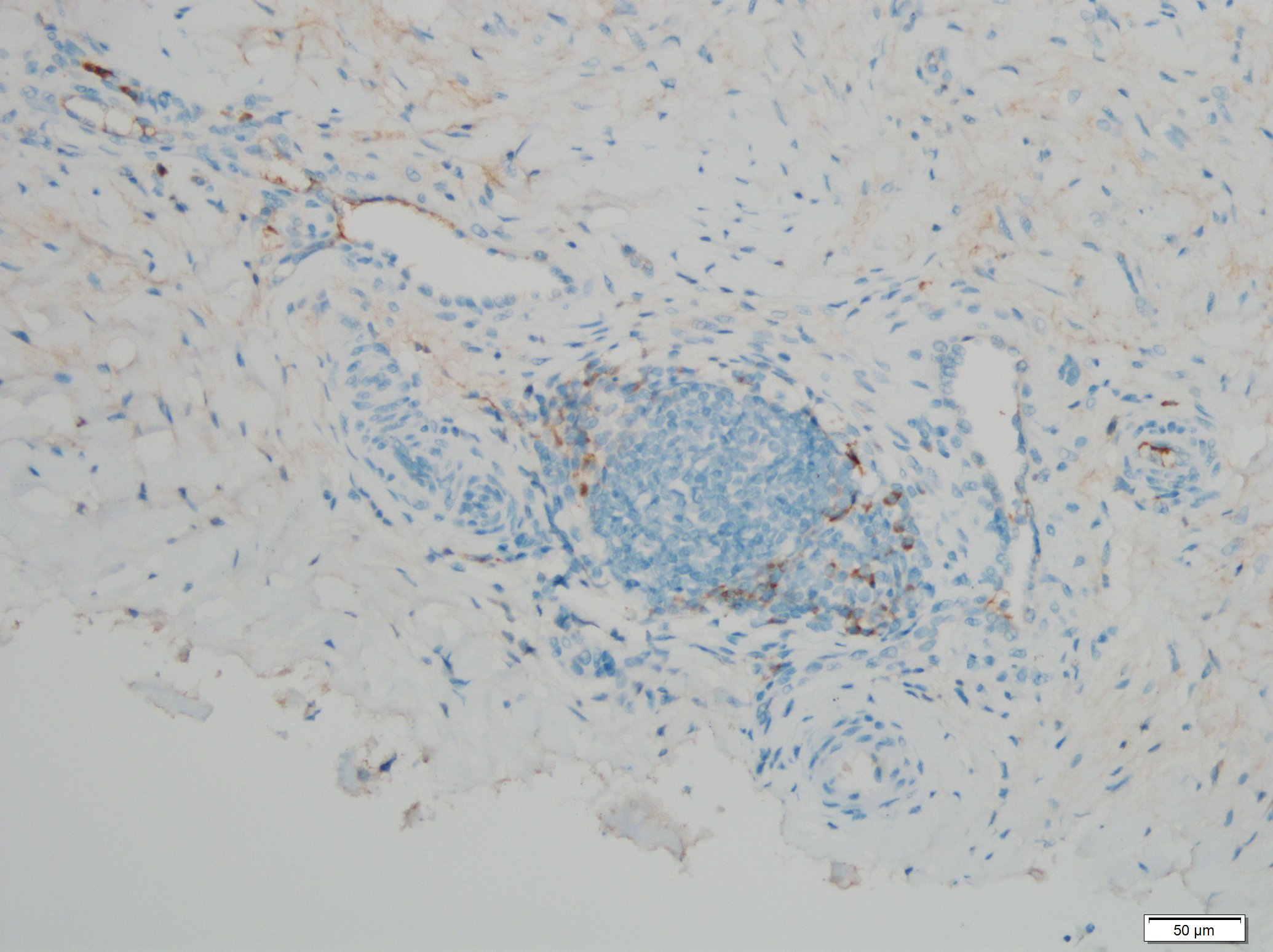

Supplement: S1 File — (ZIP) [file pone.0215499.s001.zip › CD68 and IHC stain/3 weeks/No. 3/3-1 20x-2.jpg]

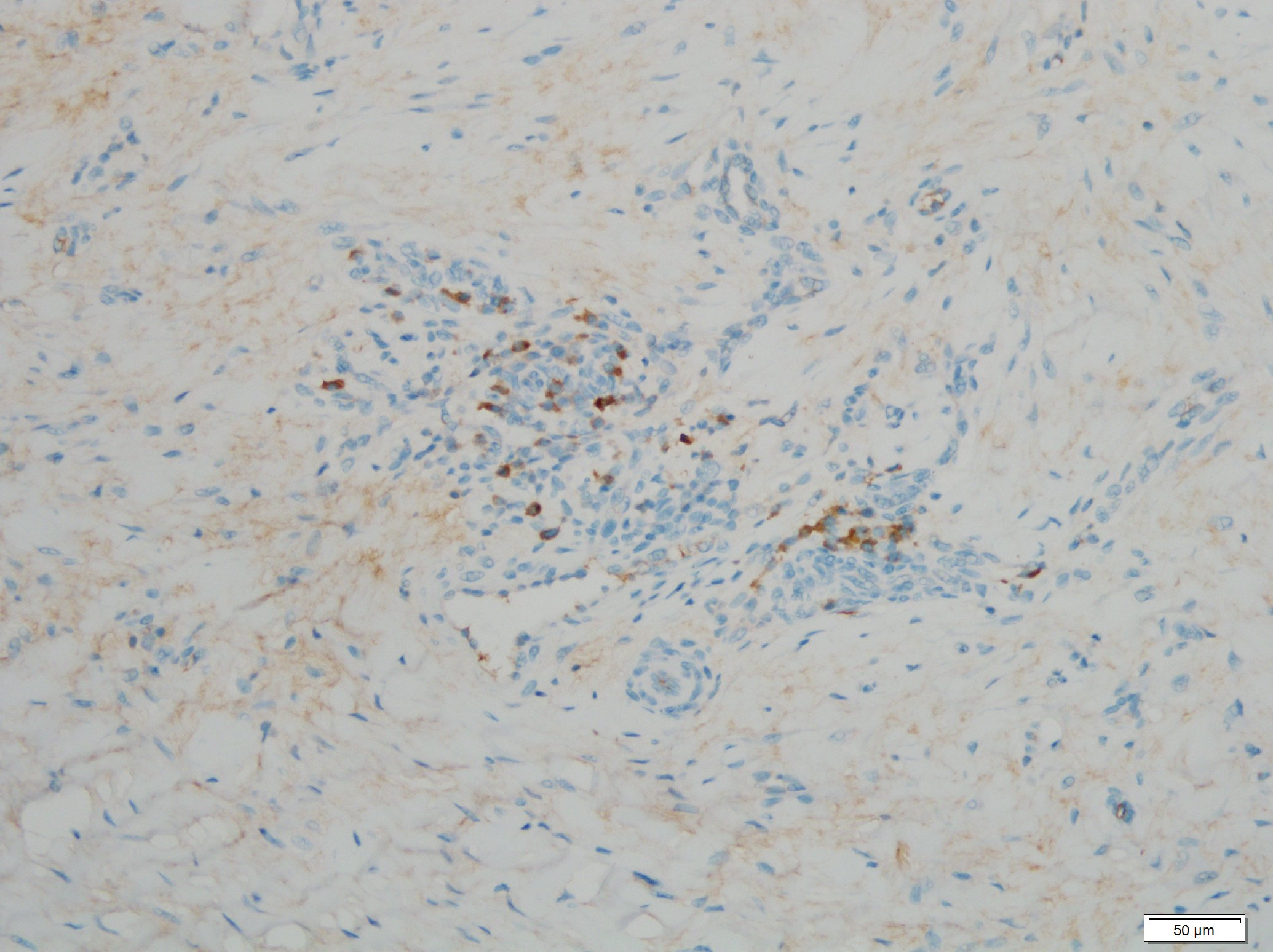

Supplement: S1 File — (ZIP) [file pone.0215499.s001.zip › CD68 and IHC stain/3 weeks/No. 3/3-1 20x-3.jpg]

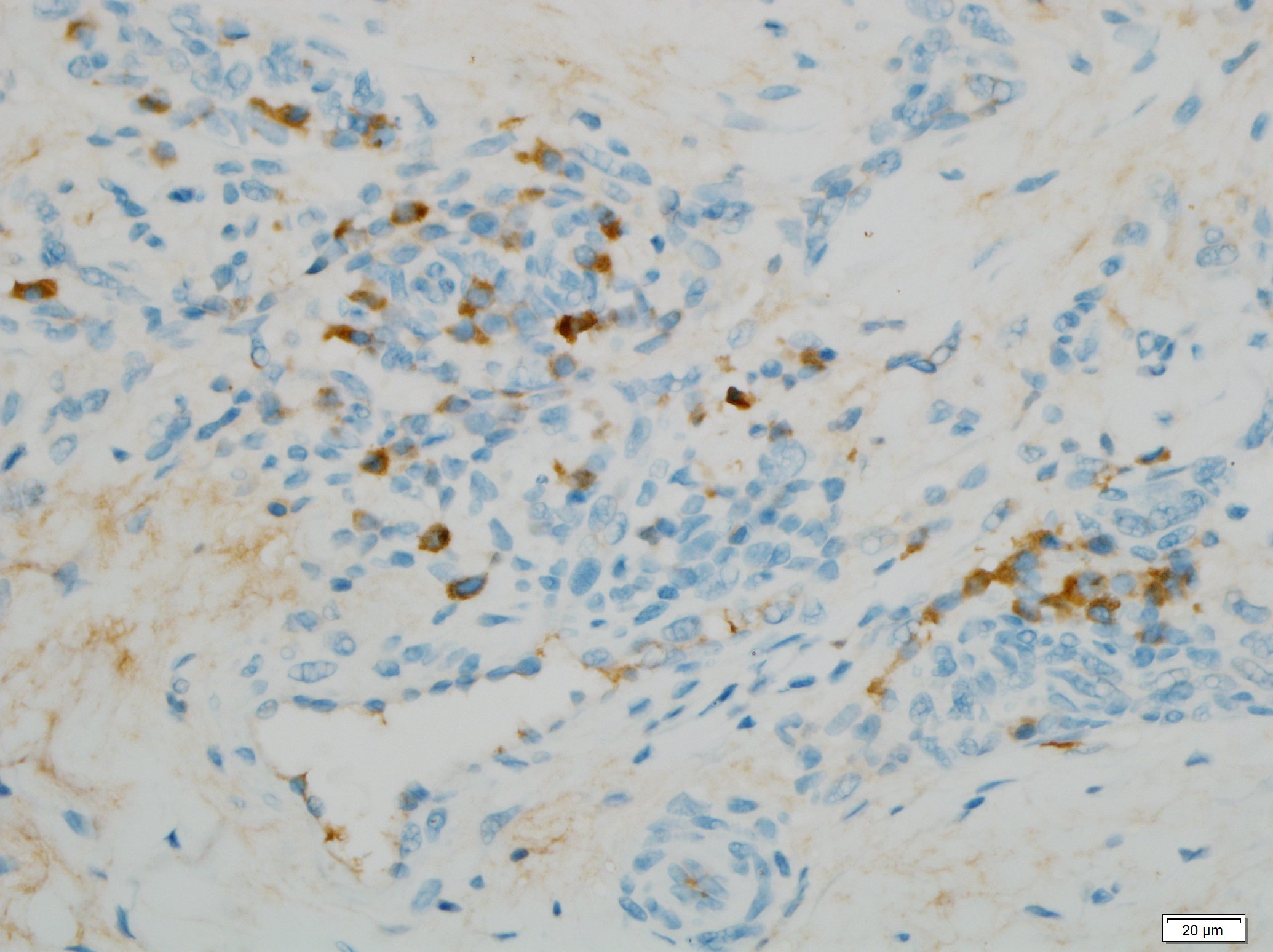

Supplement: S1 File — (ZIP) [file pone.0215499.s001.zip › CD68 and IHC stain/3 weeks/No. 3/3-1 40x-1.jpg]

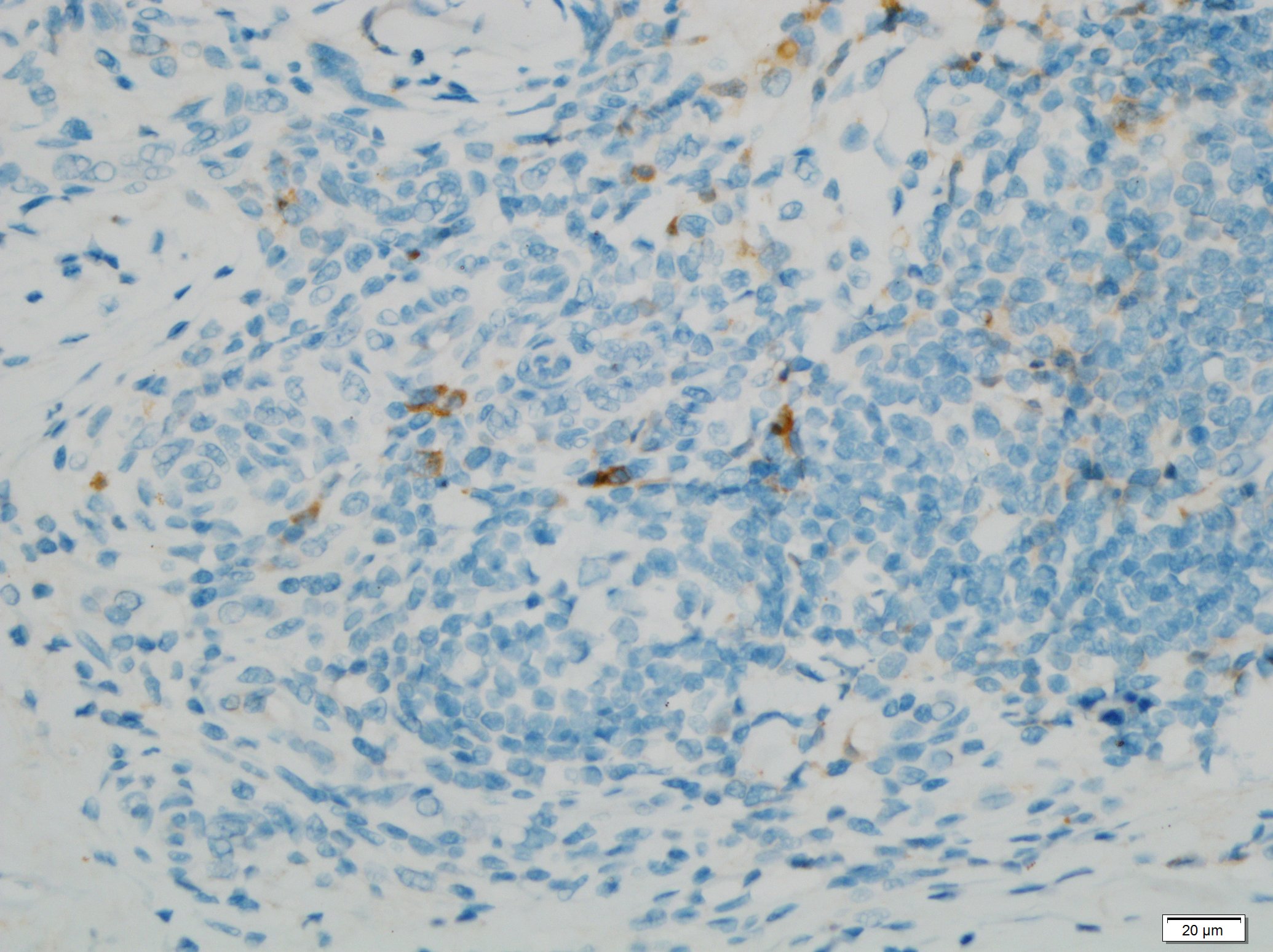

Supplement: S1 File — (ZIP) [file pone.0215499.s001.zip › CD68 and IHC stain/3 weeks/No. 3/3-1 40x-2.jpg]

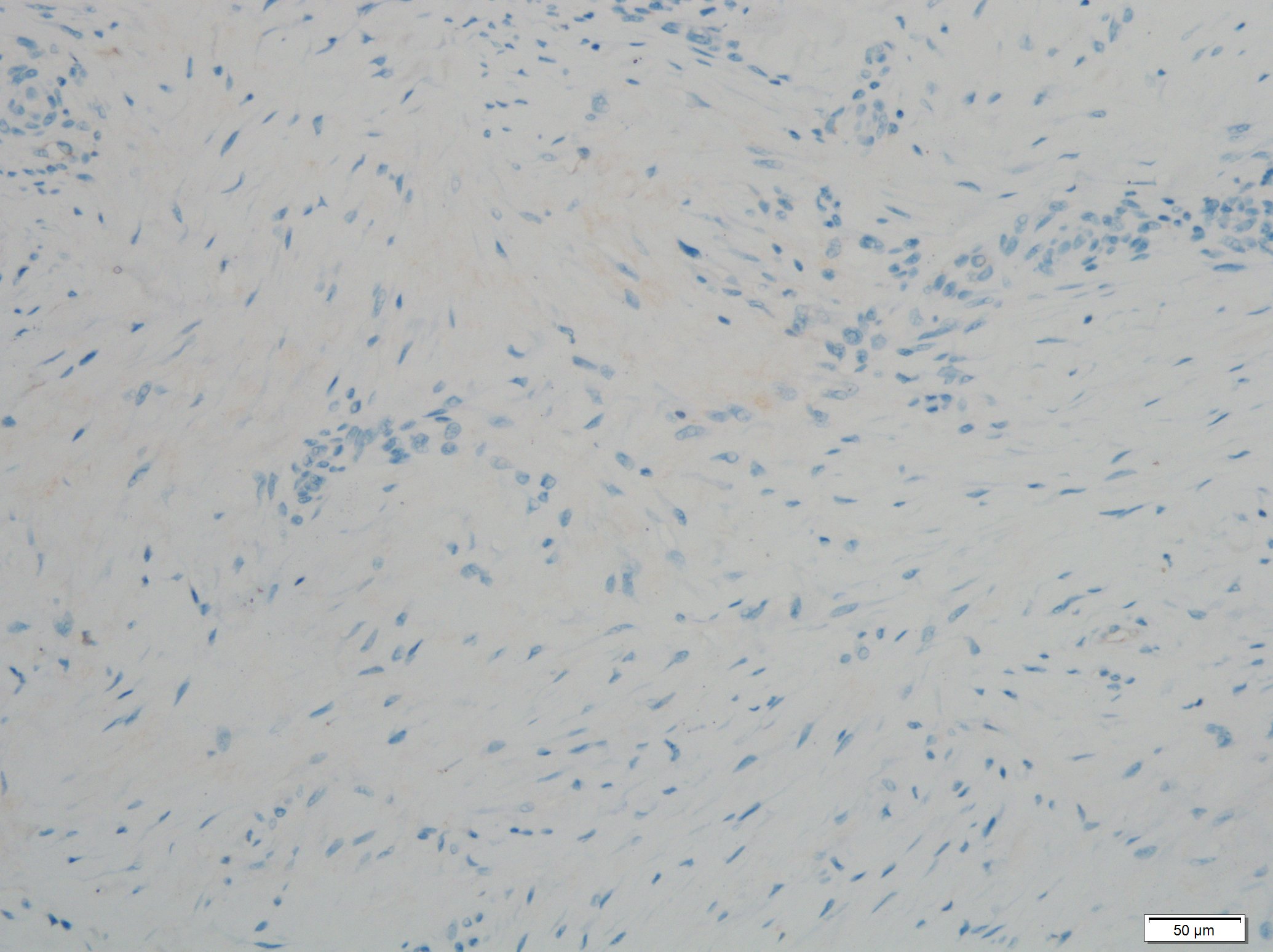

Supplement: S1 File — (ZIP) [file pone.0215499.s001.zip › CD68 and IHC stain/3 weeks/No. 3/3-2 20x-1.jpg]

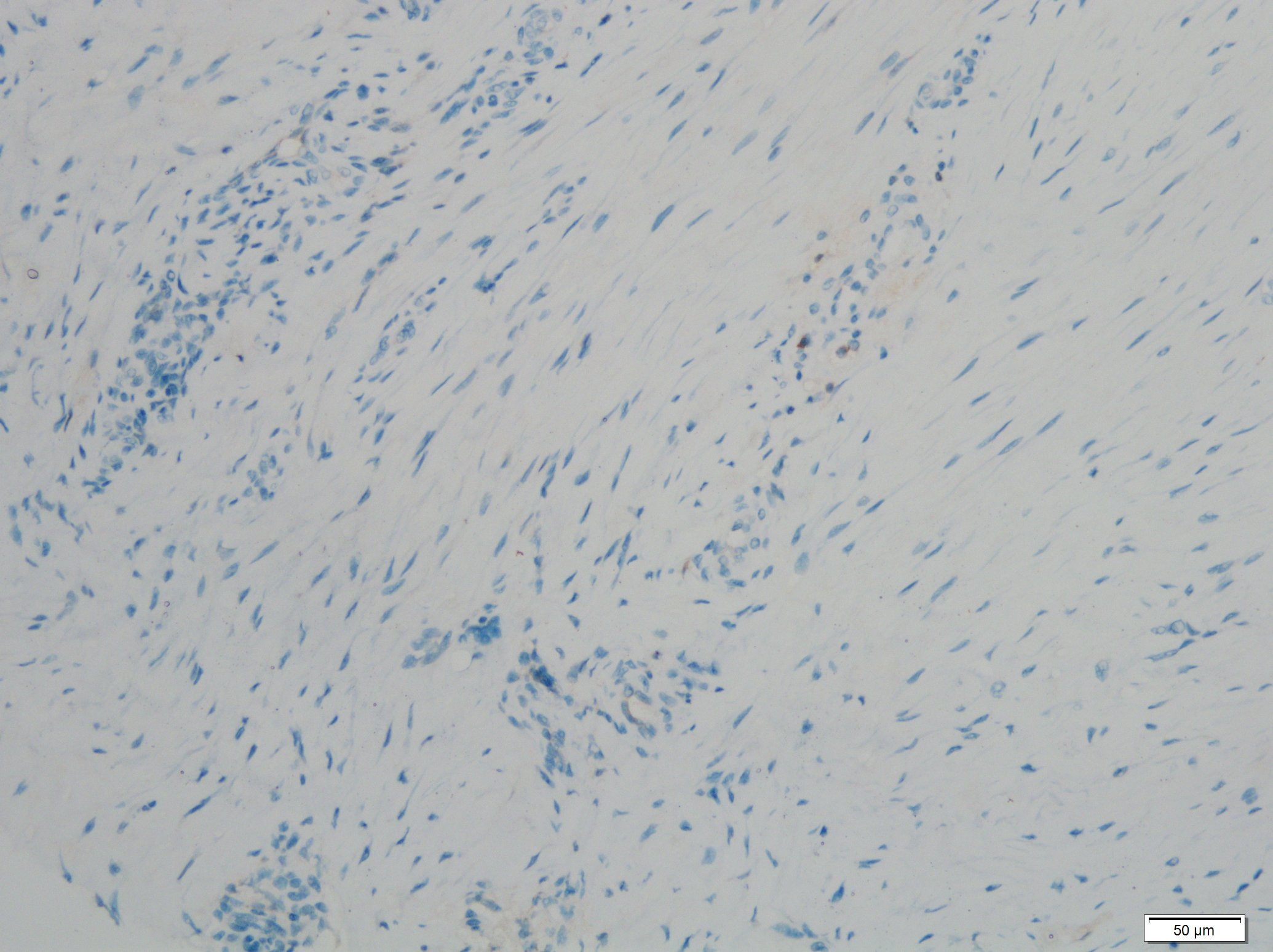

Supplement: S1 File — (ZIP) [file pone.0215499.s001.zip › CD68 and IHC stain/3 weeks/No. 3/3-2 20x-2.jpg]

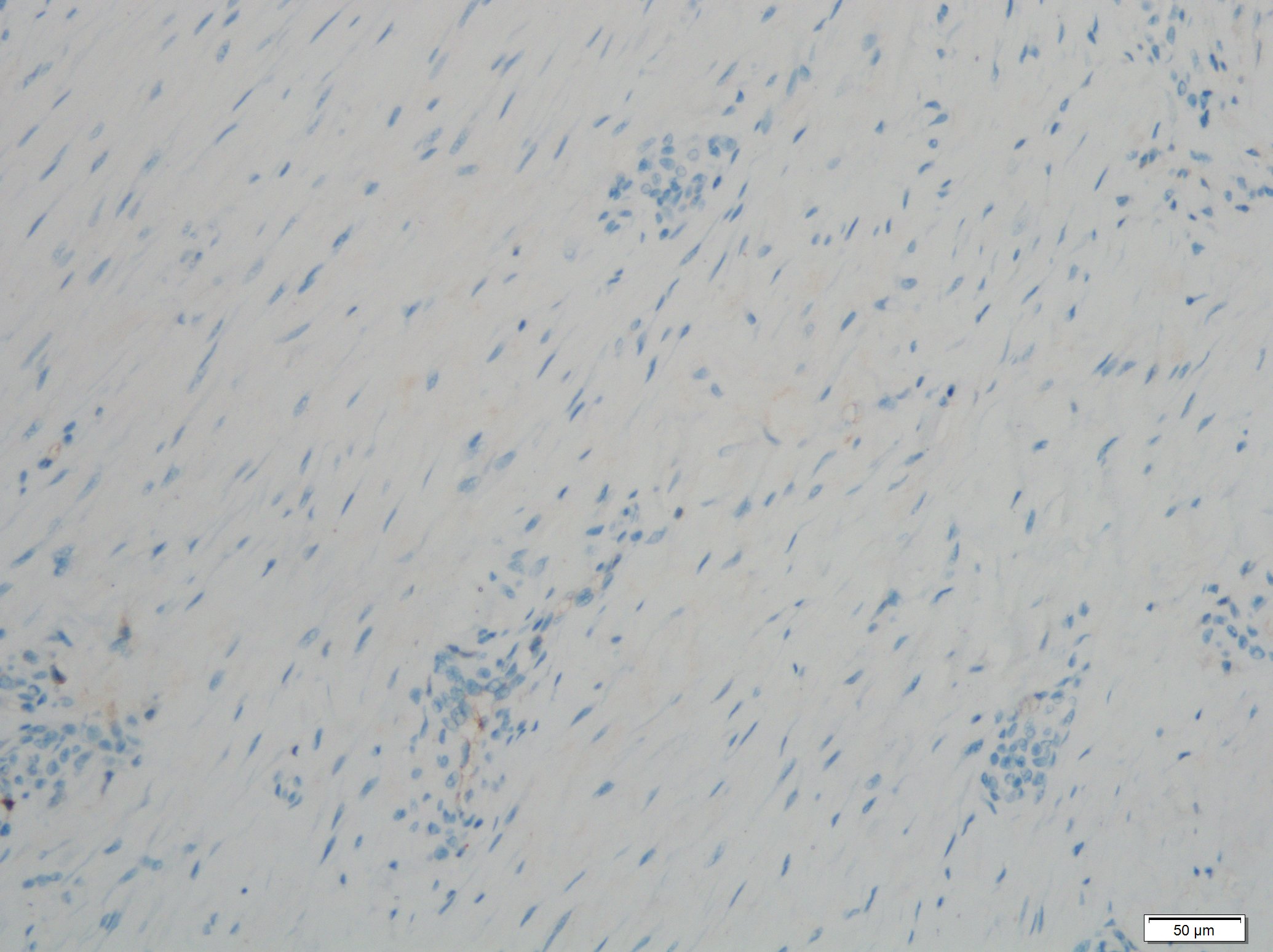

Supplement: S1 File — (ZIP) [file pone.0215499.s001.zip › CD68 and IHC stain/3 weeks/No. 3/3-2 20x-3.jpg]

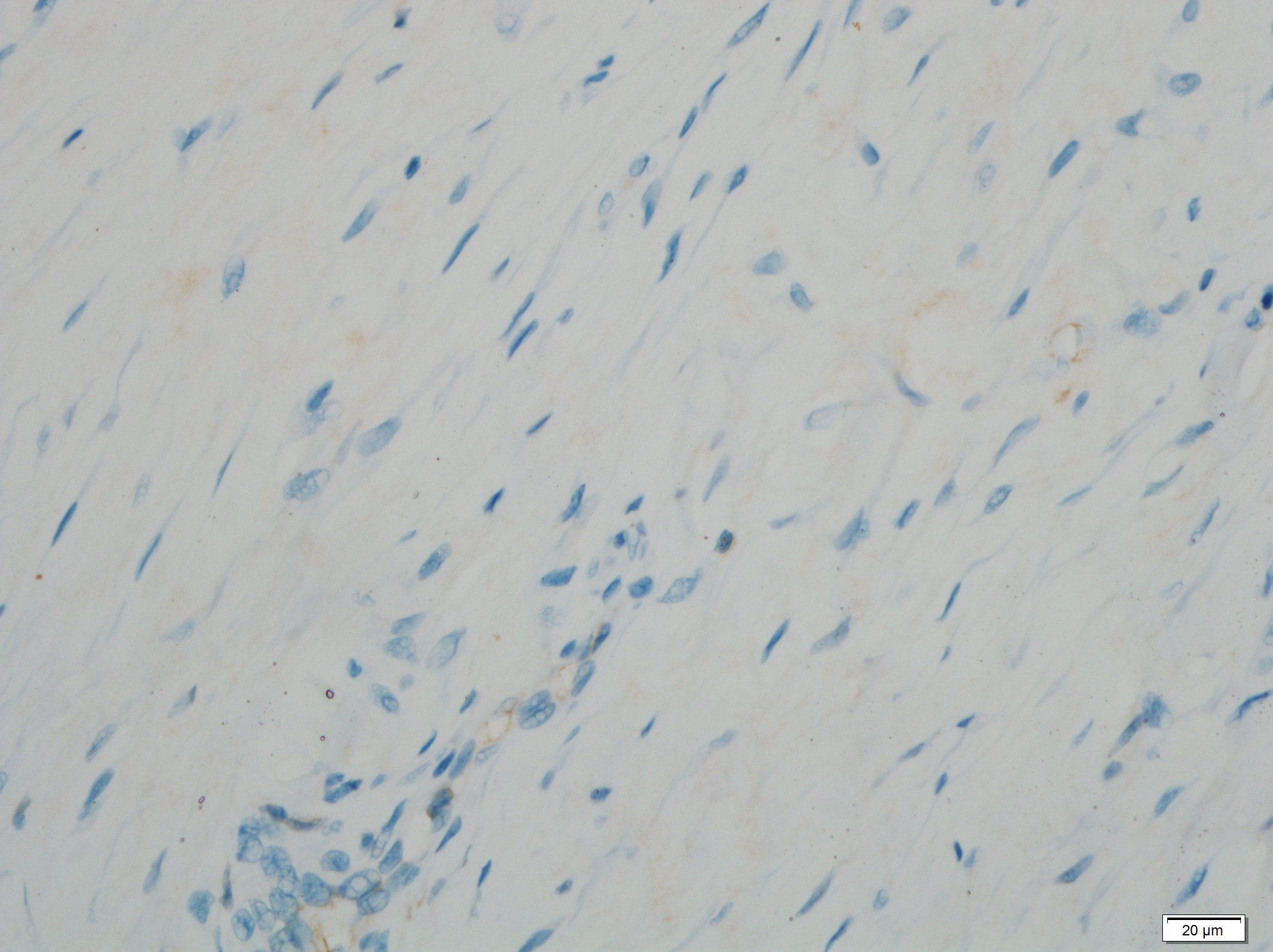

Supplement: S1 File — (ZIP) [file pone.0215499.s001.zip › CD68 and IHC stain/3 weeks/No. 3/3-2 40x-1.jpg]

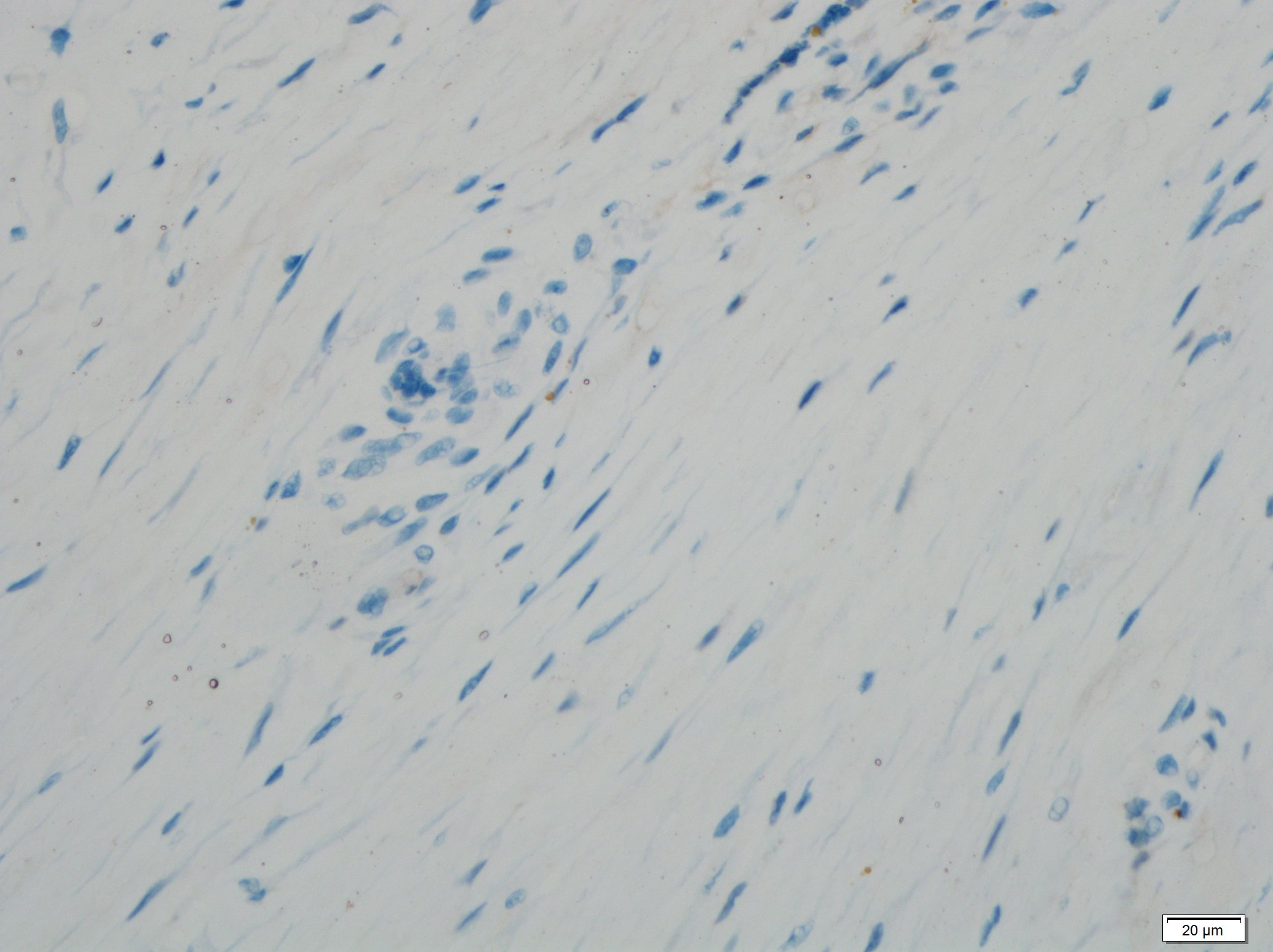

Supplement: S1 File — (ZIP) [file pone.0215499.s001.zip › CD68 and IHC stain/3 weeks/No. 3/3-2 40x-2.jpg]

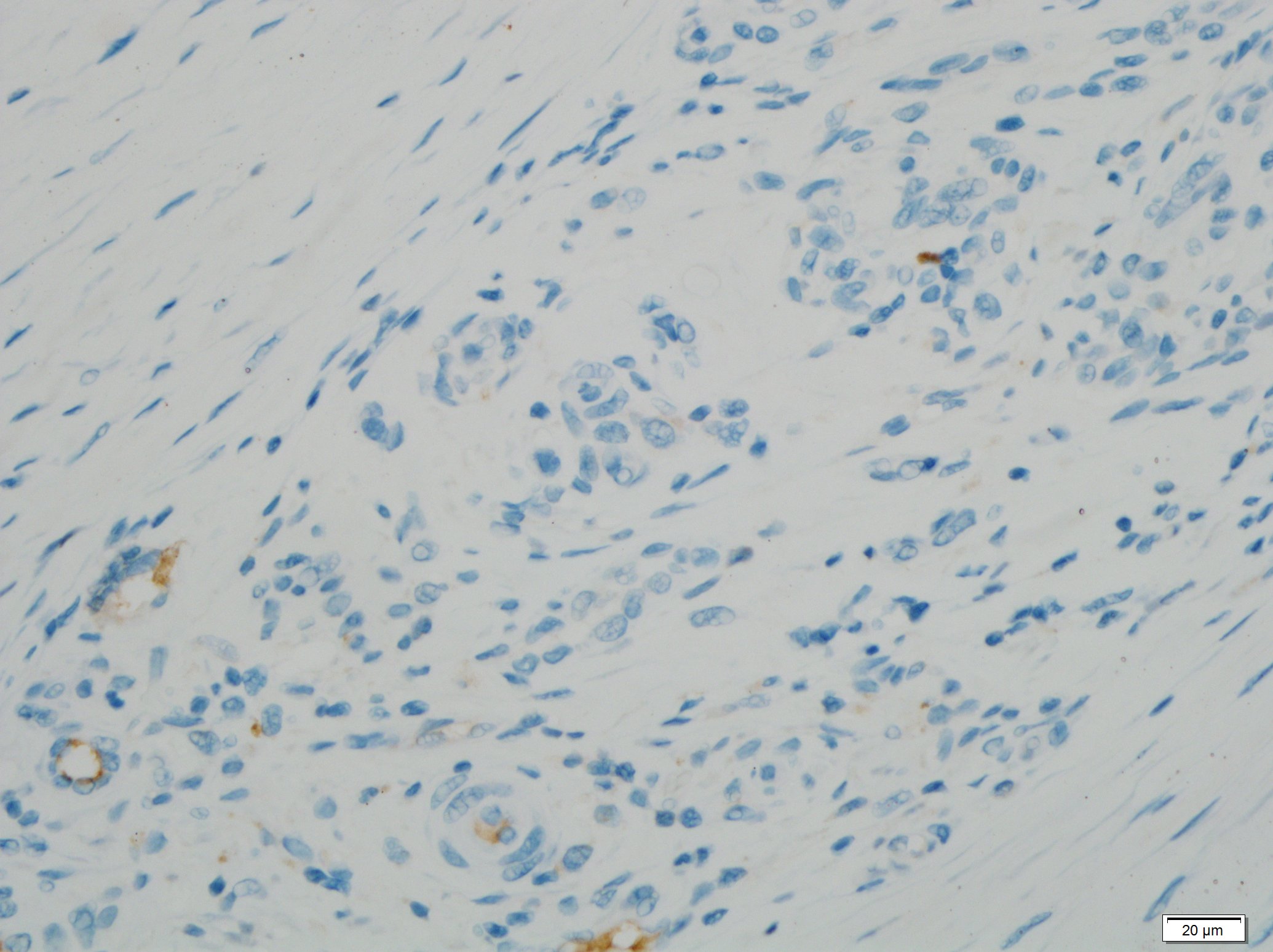

Supplement: S1 File — (ZIP) [file pone.0215499.s001.zip › CD68 and IHC stain/3 weeks/No. 3/3-2 40x-3.jpg]

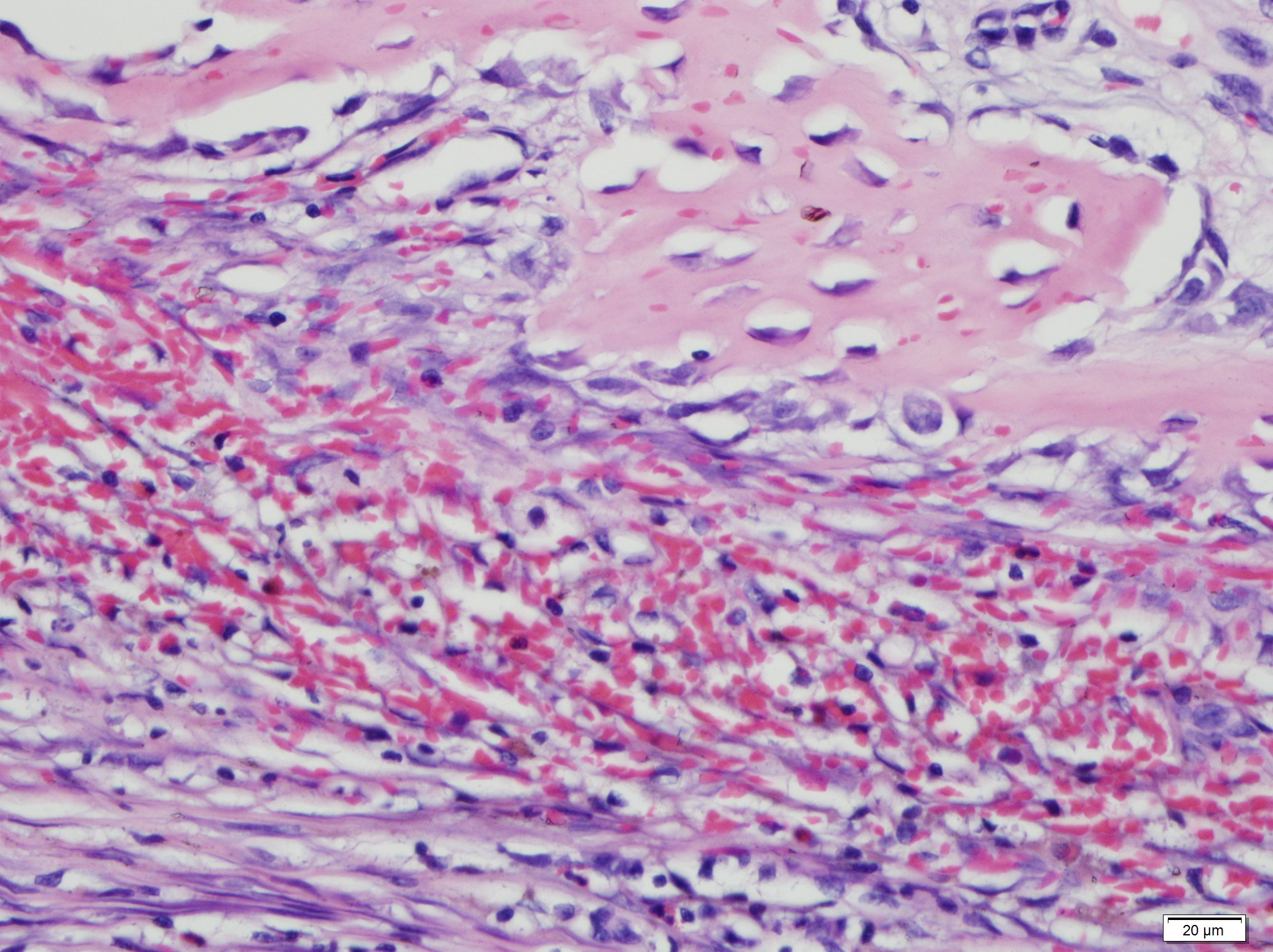

Supplement: S2 File — (ZIP) [file pone.0215499.s002.zip › h&e stain data/1 week/7-1 40x-3.jpg]

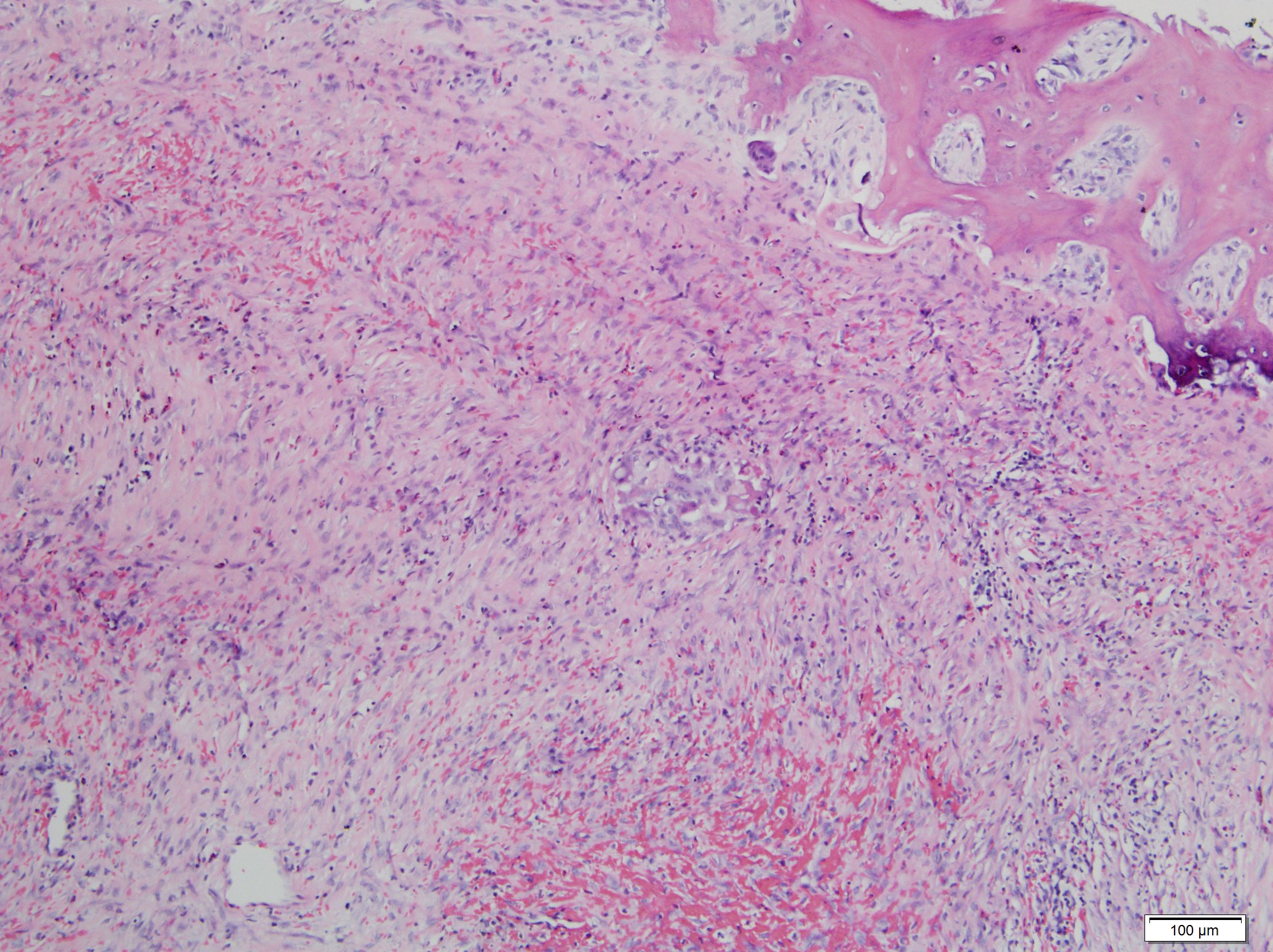

Supplement: S2 File — (ZIP) [file pone.0215499.s002.zip › h&e stain data/1 week/7-3 10x-2.jpg]

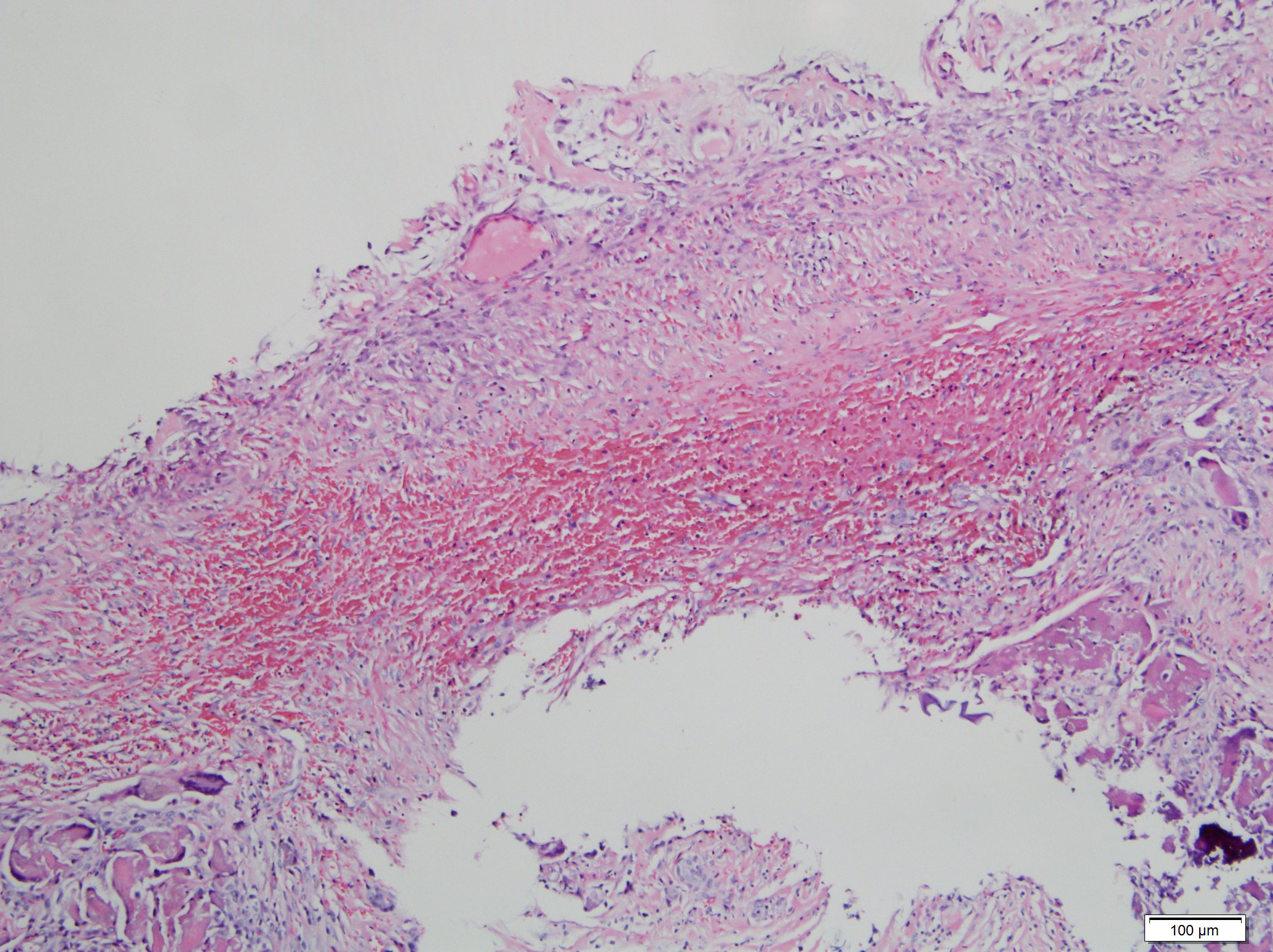

Supplement: S2 File — (ZIP) [file pone.0215499.s002.zip › h&e stain data/1 week/7-3 10x-3.jpg]

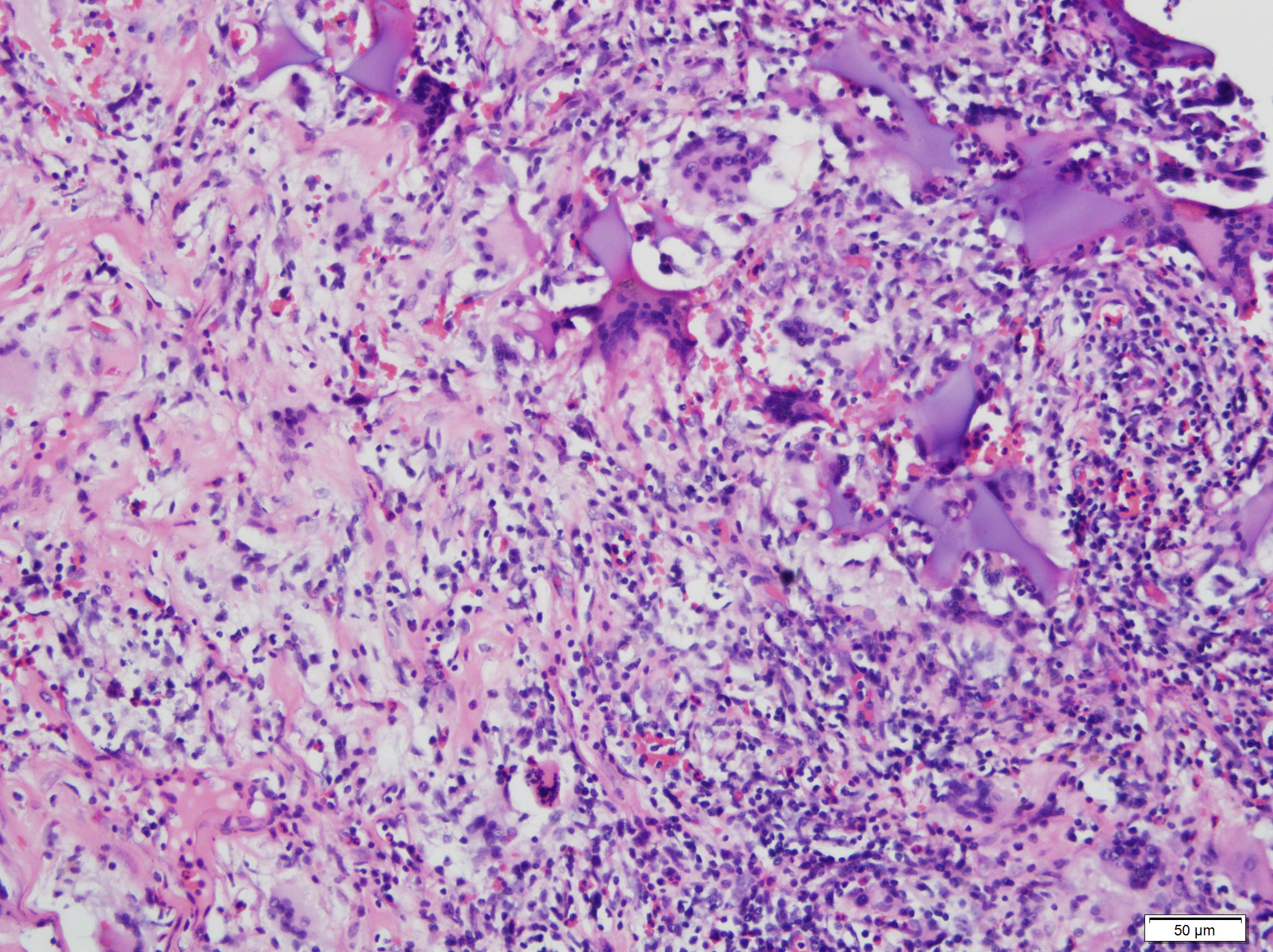

Supplement: S2 File — (ZIP) [file pone.0215499.s002.zip › h&e stain data/1 week/7-4 20x-3.jpg]

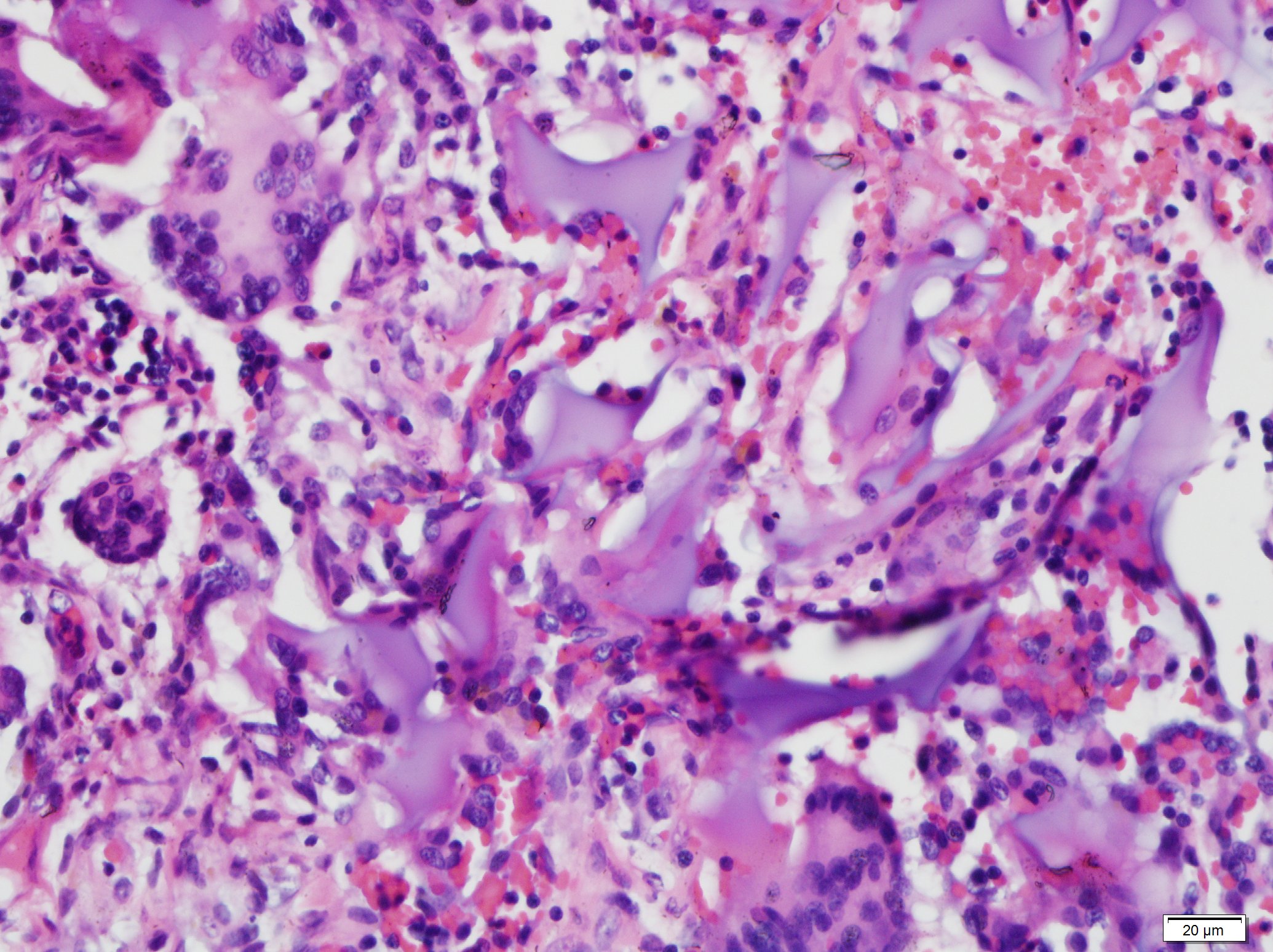

Supplement: S2 File — (ZIP) [file pone.0215499.s002.zip › h&e stain data/1 week/7-4 40x-2.jpg]

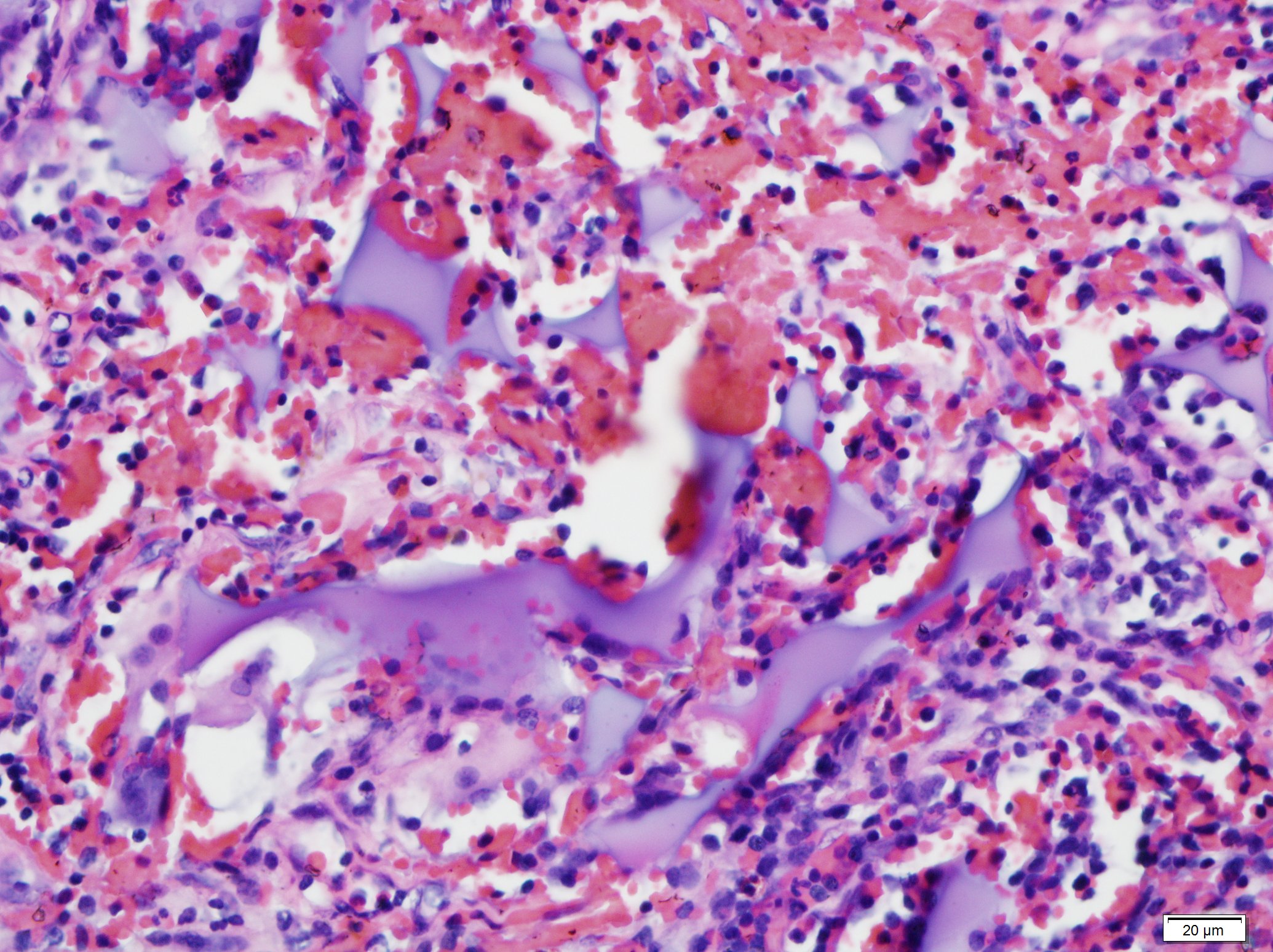

Supplement: S2 File — (ZIP) [file pone.0215499.s002.zip › h&e stain data/1 week/7-4 40x-3.jpg]

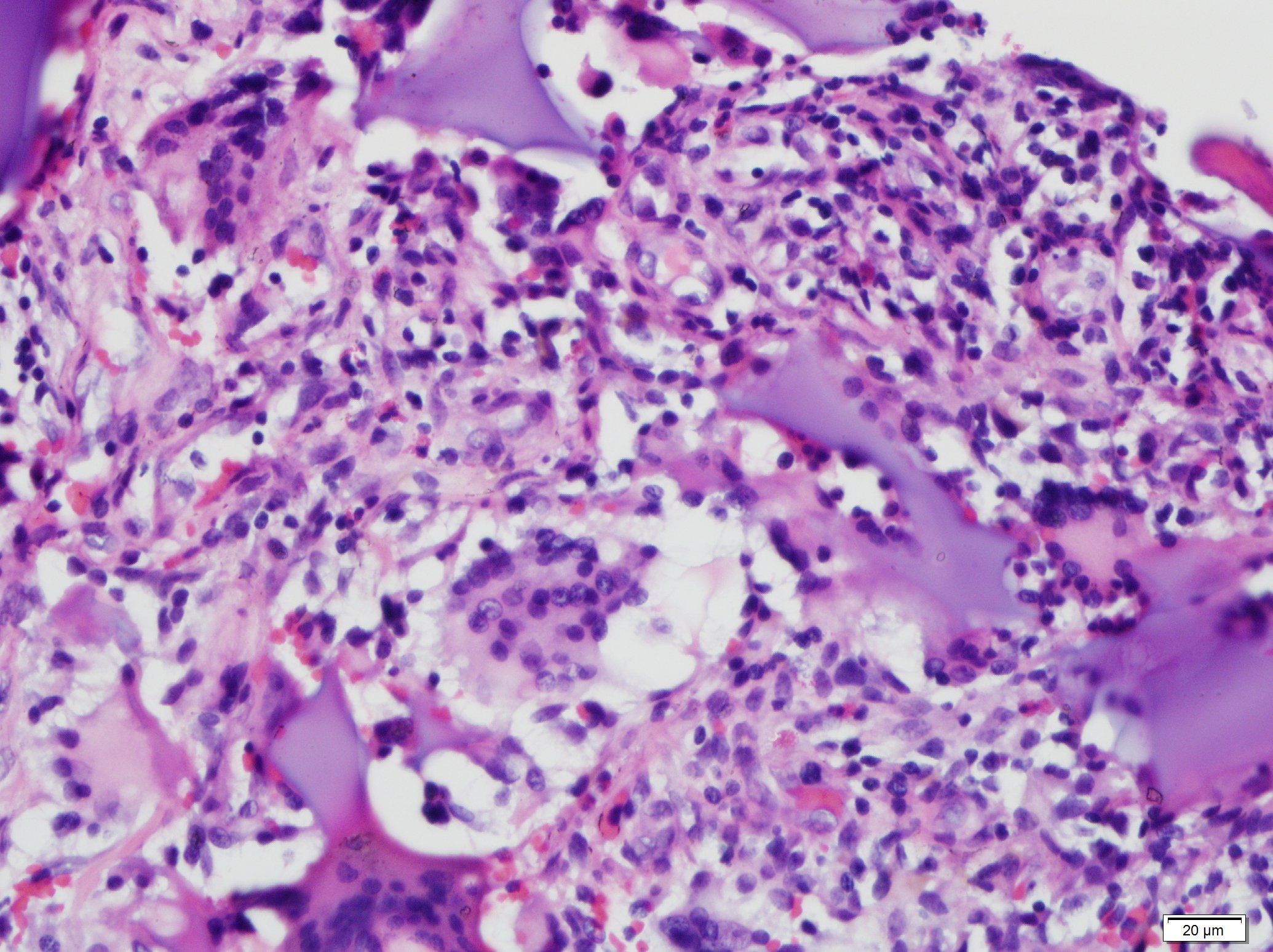

Supplement: S2 File — (ZIP) [file pone.0215499.s002.zip › h&e stain data/1 week/7-4 40x.jpg]

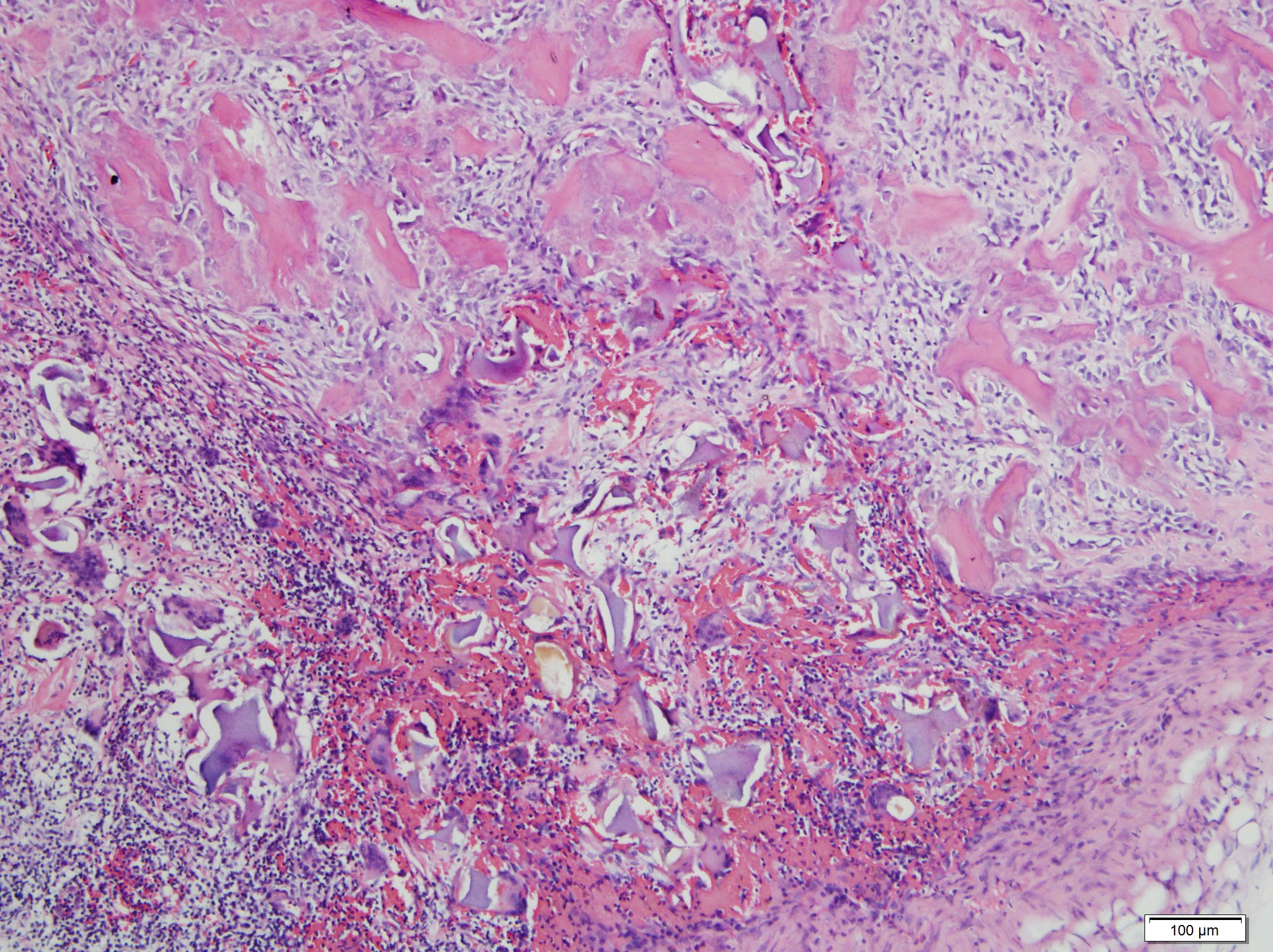

Supplement: S2 File — (ZIP) [file pone.0215499.s002.zip › h&e stain data/1 week/7-6 10x.jpg]

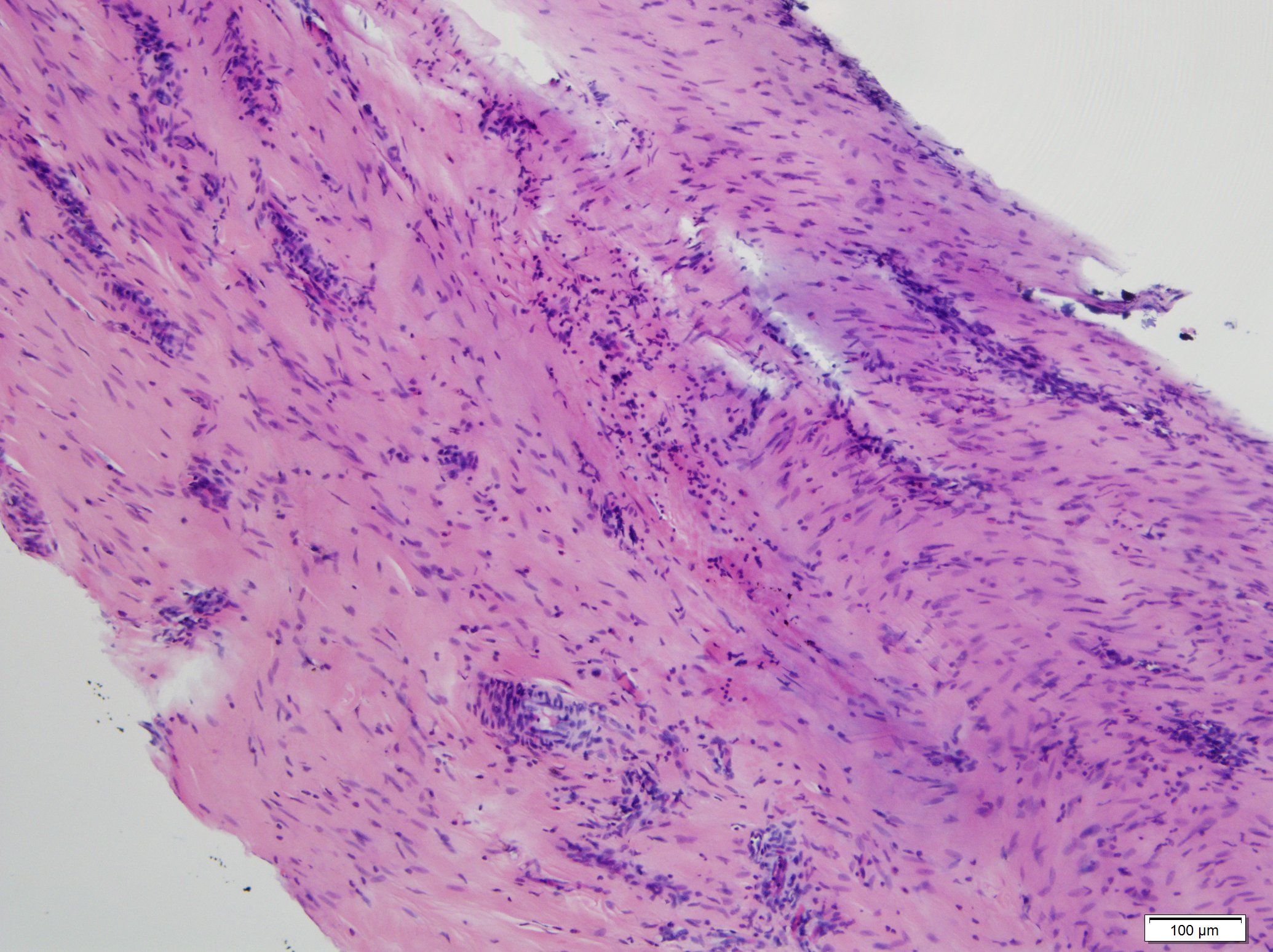

Supplement: S2 File — (ZIP) [file pone.0215499.s002.zip › h&e stain data/2 weeks/6-1 10x-2.jpg]

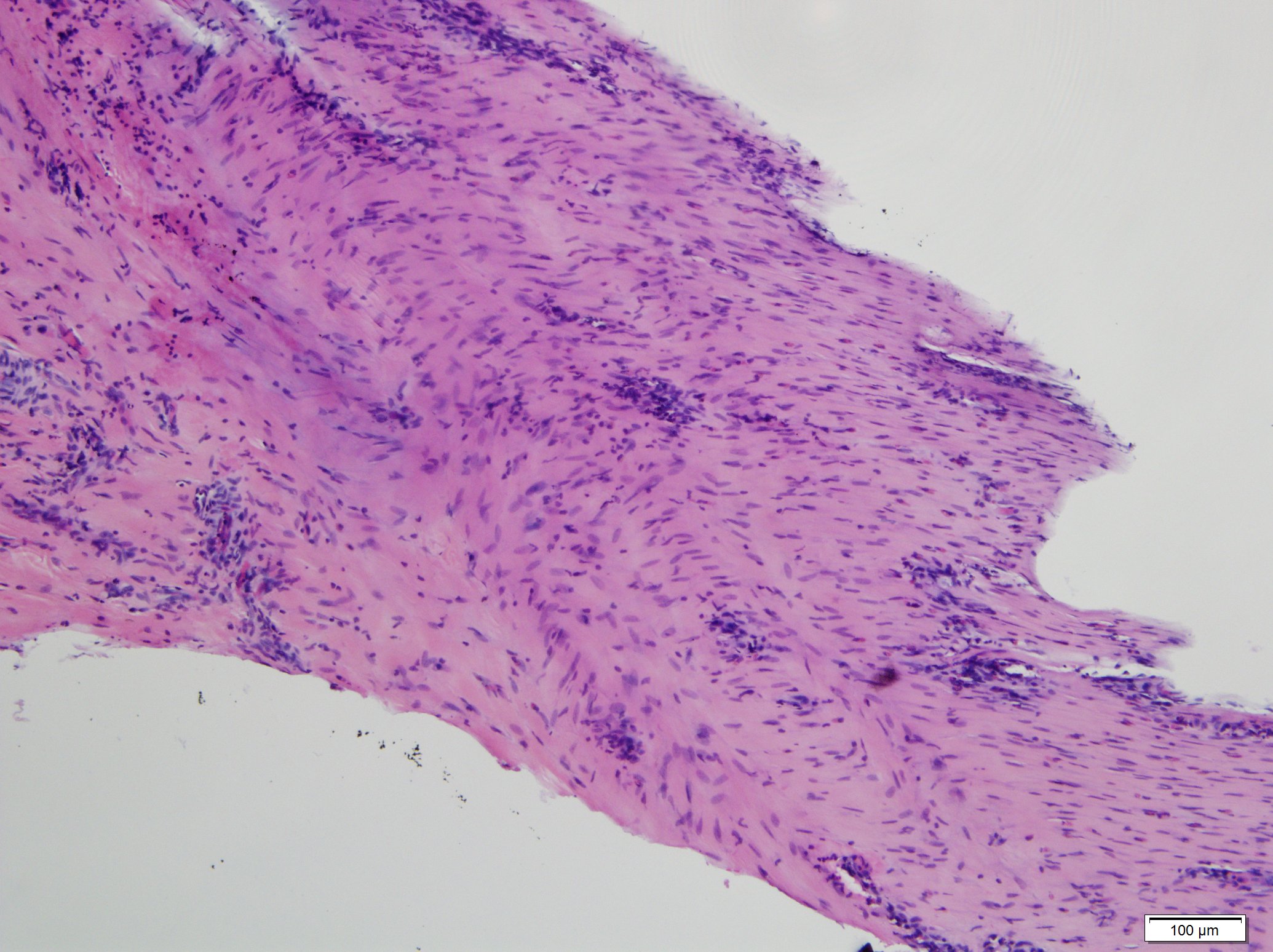

Supplement: S2 File — (ZIP) [file pone.0215499.s002.zip › h&e stain data/2 weeks/6-1 10x-3.jpg]

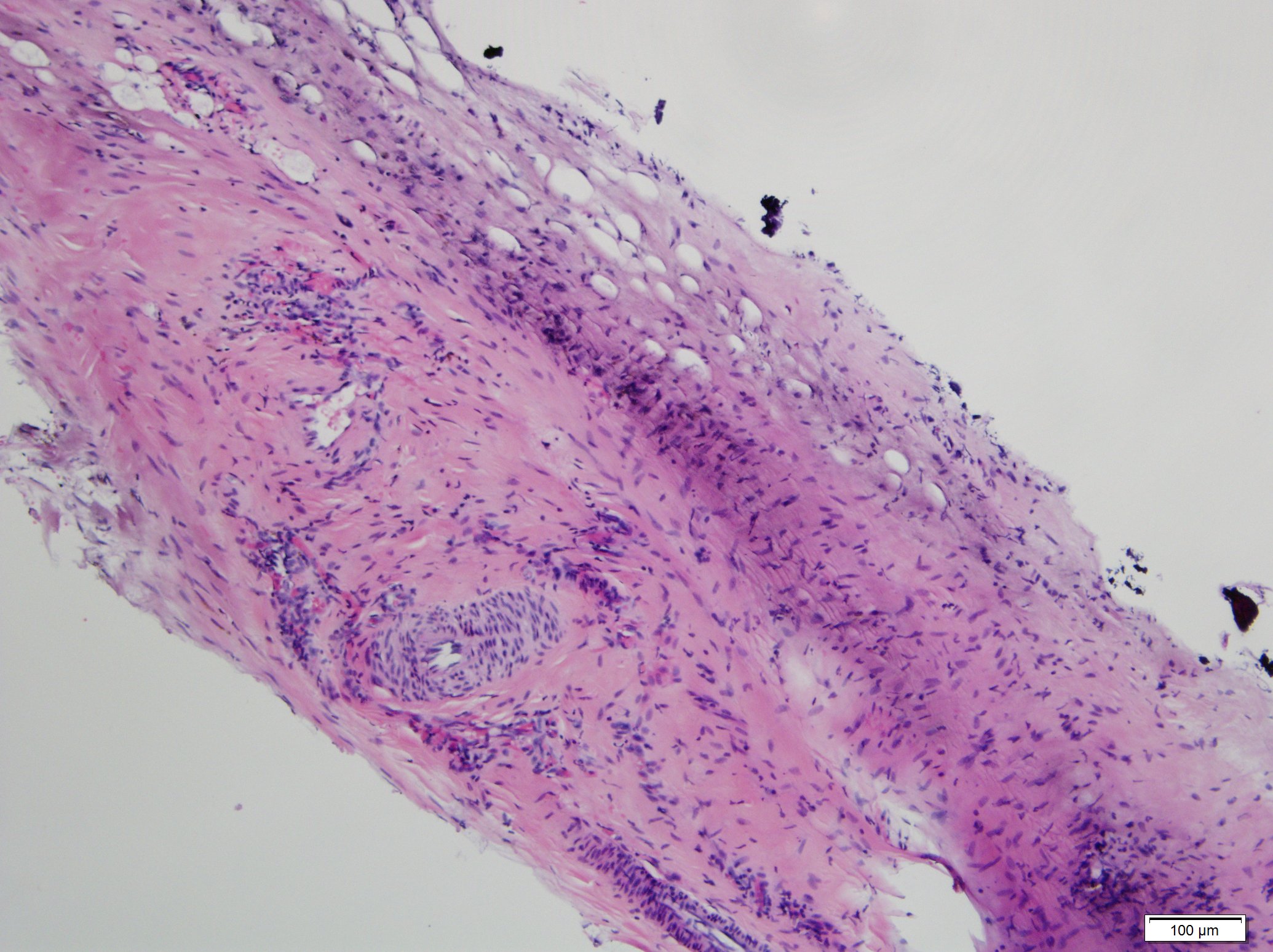

Supplement: S2 File — (ZIP) [file pone.0215499.s002.zip › h&e stain data/2 weeks/6-1 10x.jpg]

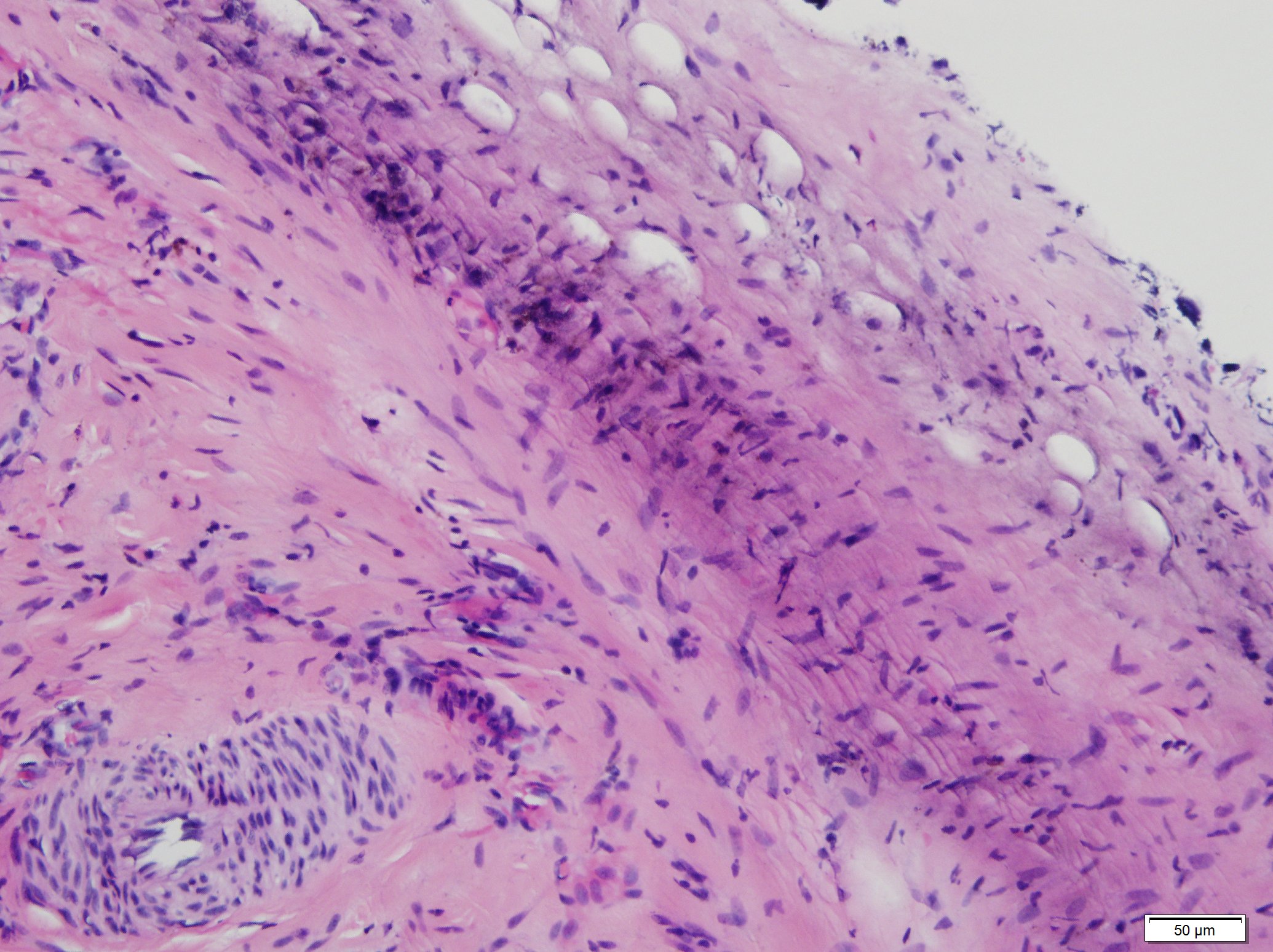

Supplement: S2 File — (ZIP) [file pone.0215499.s002.zip › h&e stain data/2 weeks/6-1 20x-2.jpg]

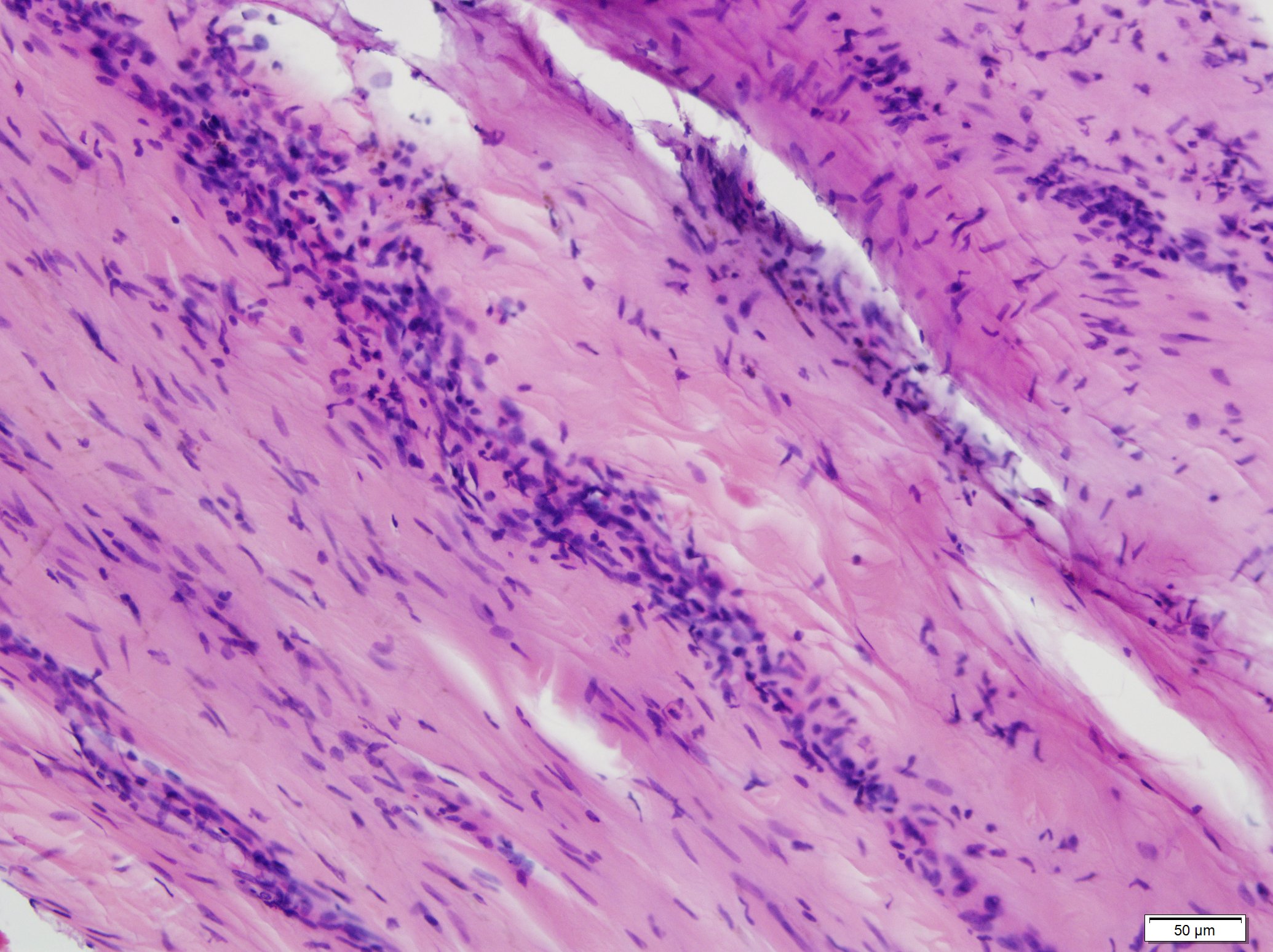

Supplement: S2 File — (ZIP) [file pone.0215499.s002.zip › h&e stain data/2 weeks/6-1 20x-3.jpg]

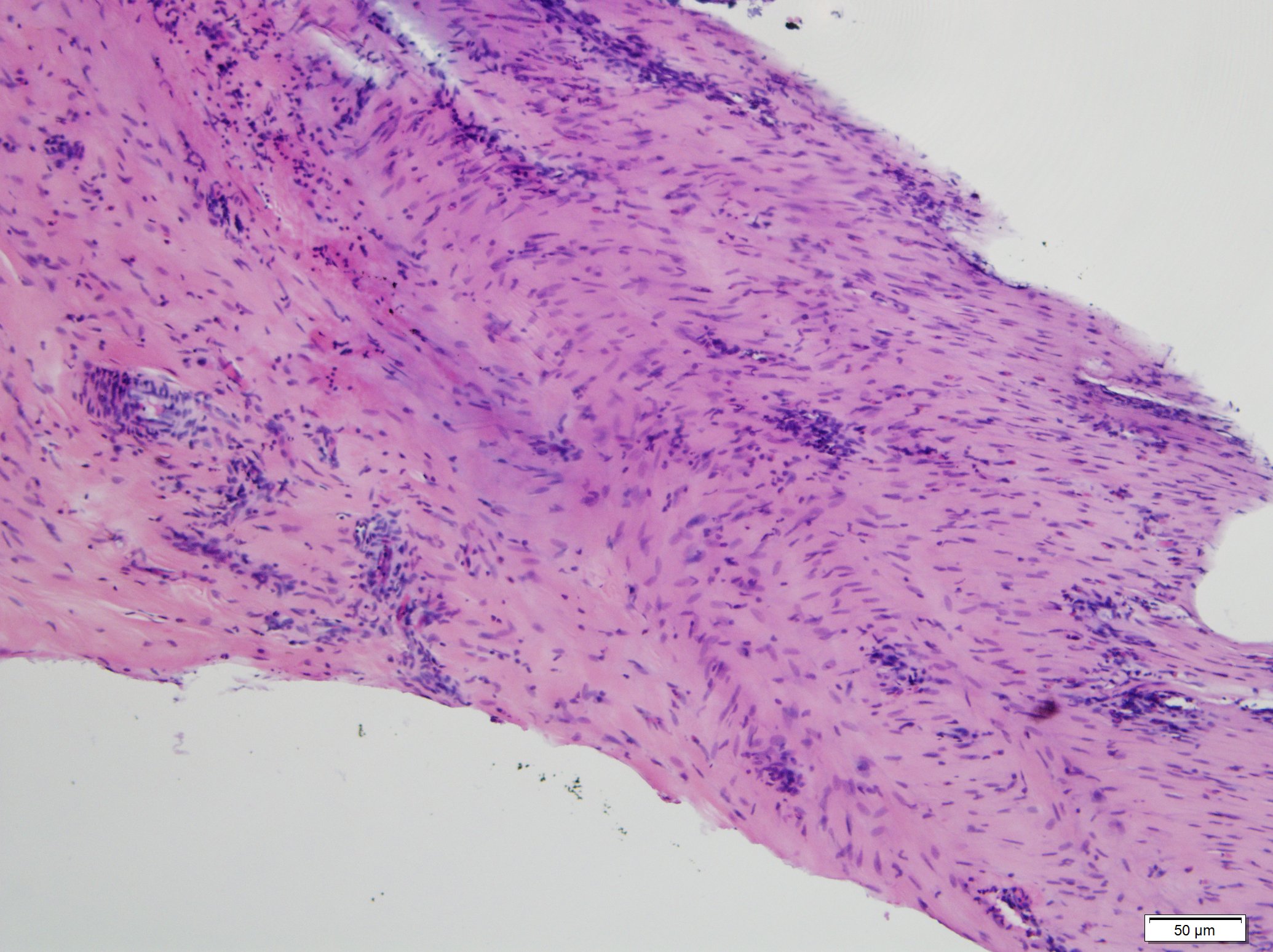

Supplement: S2 File — (ZIP) [file pone.0215499.s002.zip › h&e stain data/2 weeks/6-1 20x.jpg]

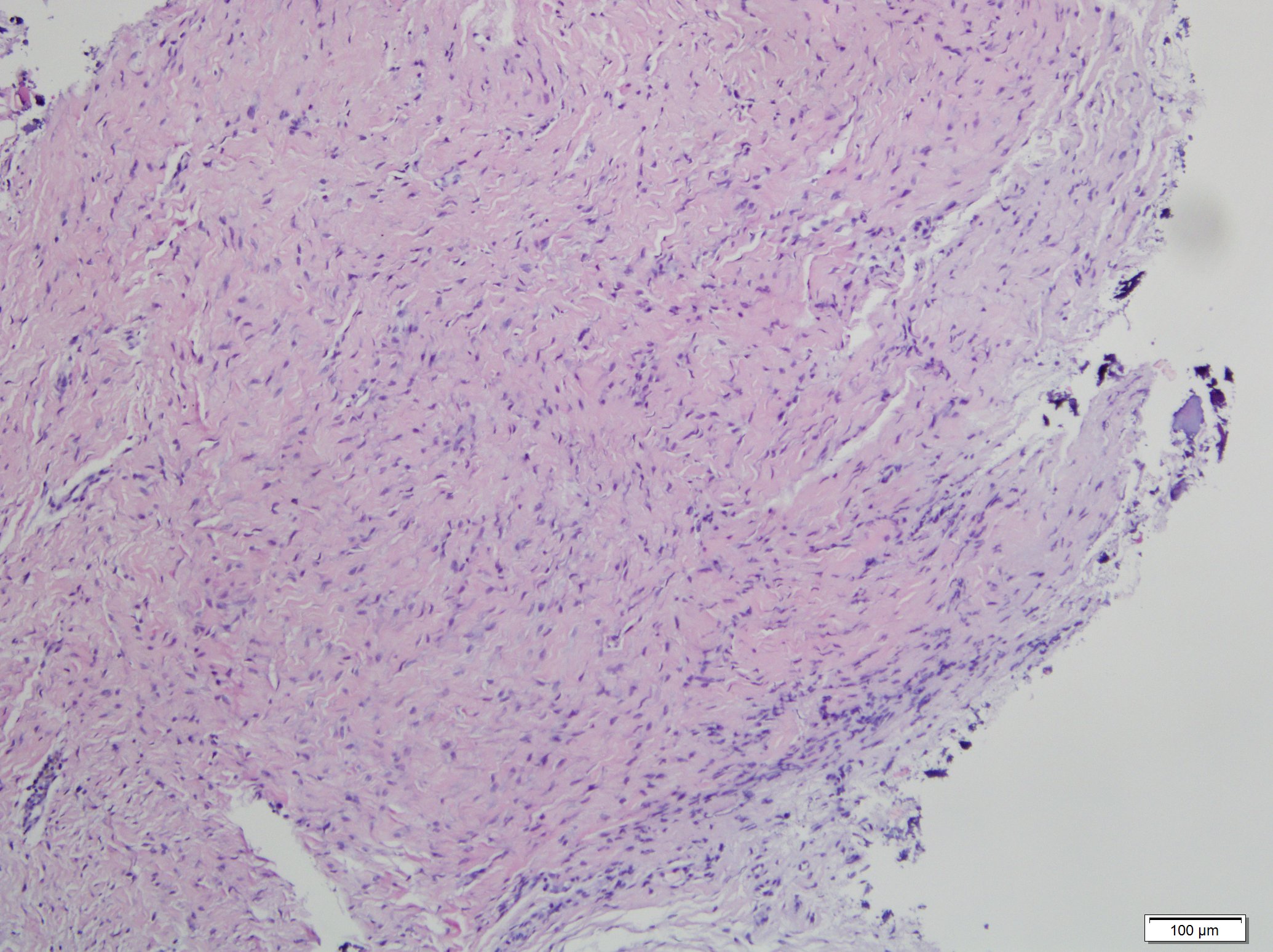

Supplement: S2 File — (ZIP) [file pone.0215499.s002.zip › h&e stain data/2 weeks/6-2 10x-2.jpg]

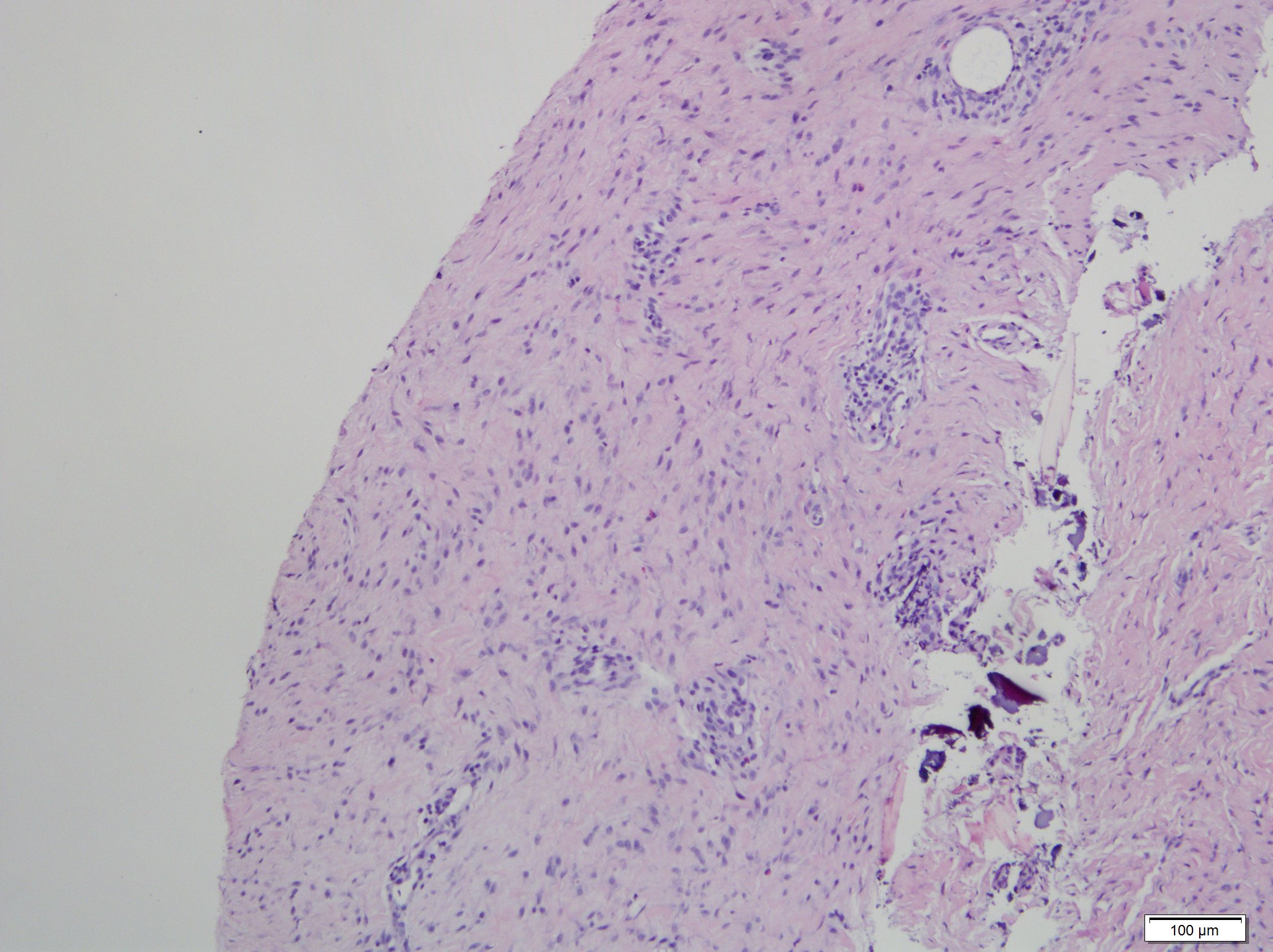

Supplement: S2 File — (ZIP) [file pone.0215499.s002.zip › h&e stain data/2 weeks/6-2 10x-3.jpg]

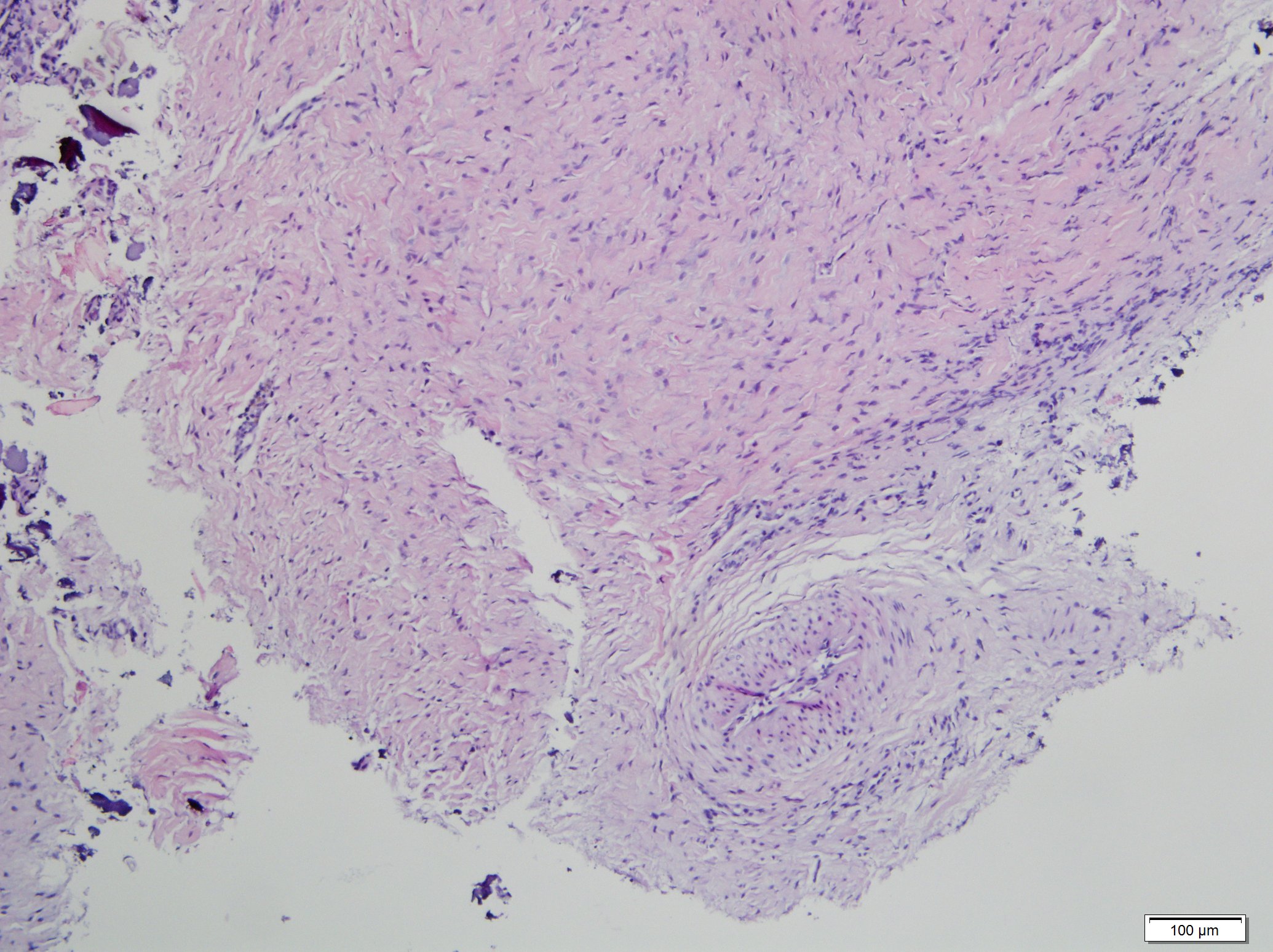

Supplement: S2 File — (ZIP) [file pone.0215499.s002.zip › h&e stain data/2 weeks/6-2 10x.jpg]

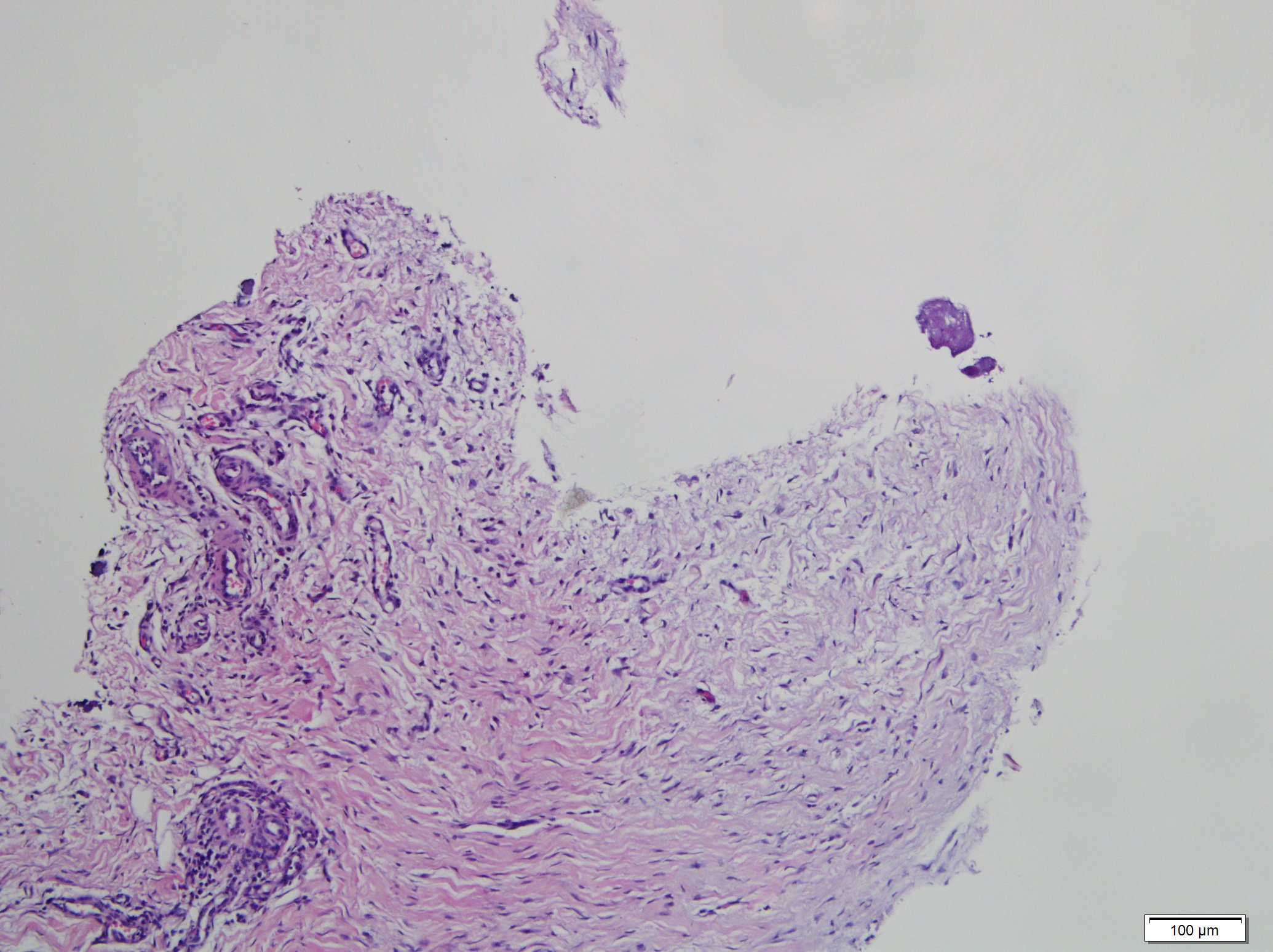

Supplement: S2 File — (ZIP) [file pone.0215499.s002.zip › h&e stain data/2 weeks/6-3 10x-3.jpg]

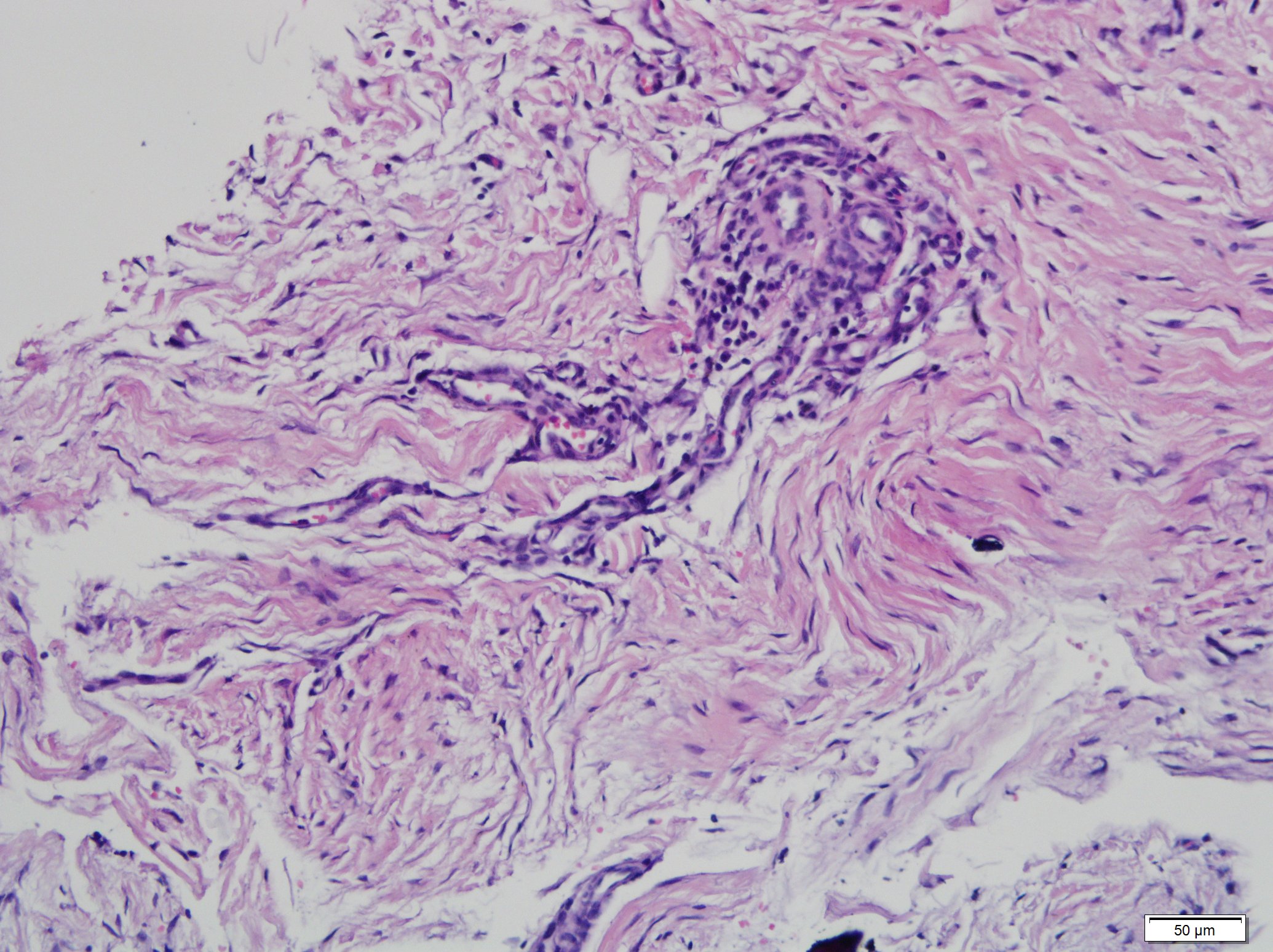

Supplement: S2 File — (ZIP) [file pone.0215499.s002.zip › h&e stain data/2 weeks/6-3 20x-2.jpg]

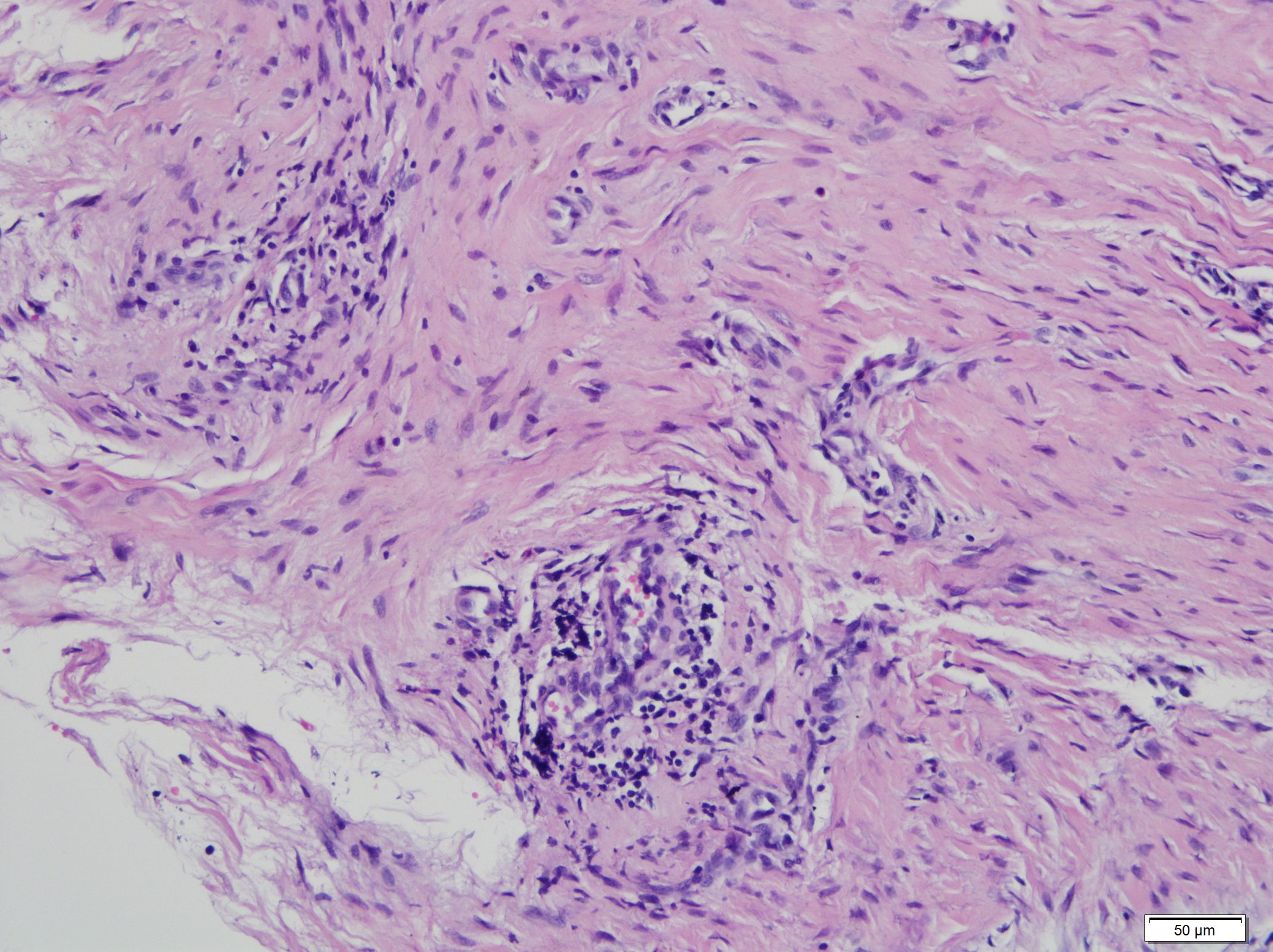

Supplement: S2 File — (ZIP) [file pone.0215499.s002.zip › h&e stain data/2 weeks/6-3 20x-3.jpg]

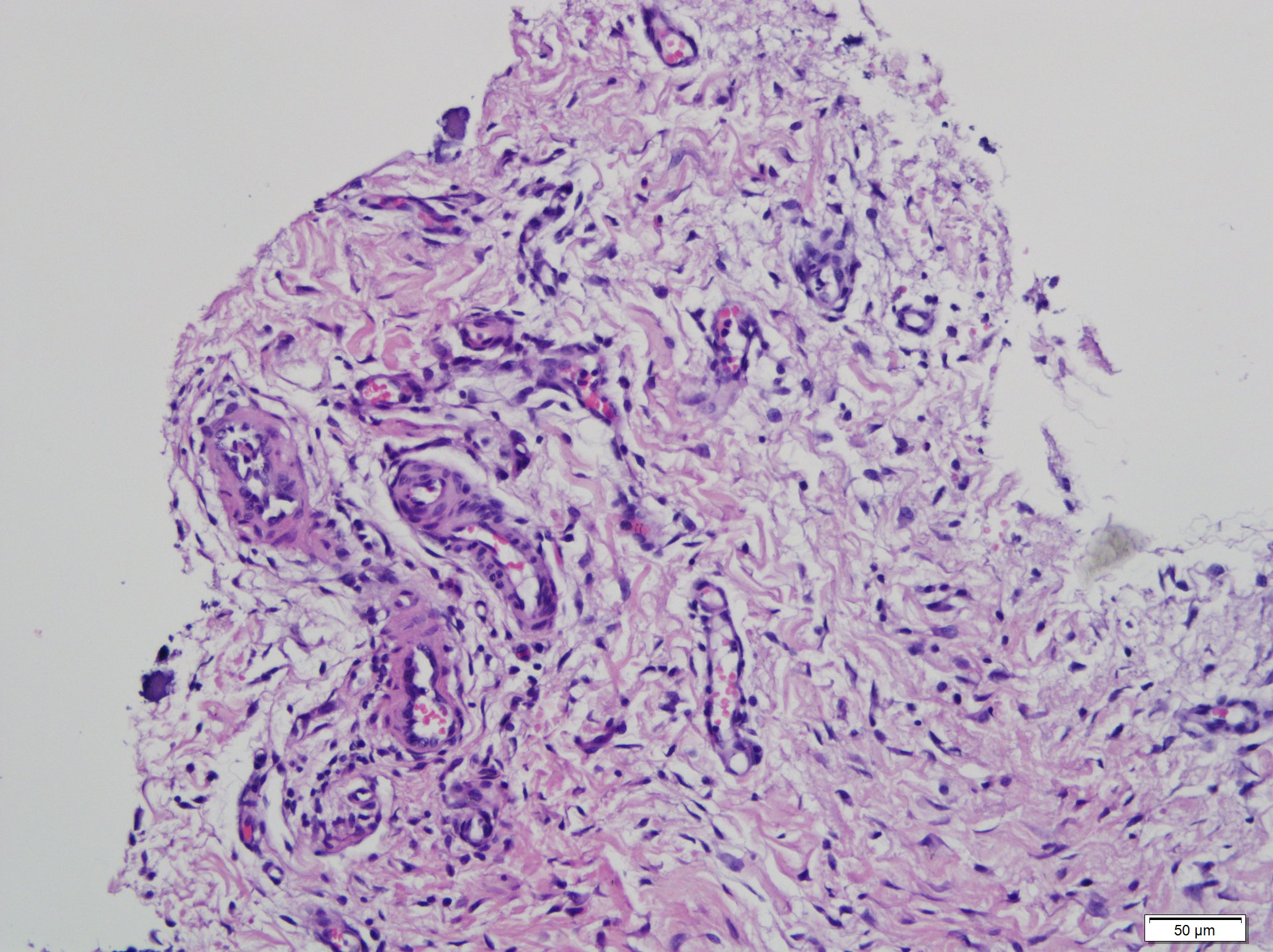

Supplement: S2 File — (ZIP) [file pone.0215499.s002.zip › h&e stain data/2 weeks/6-3 20x.jpg]

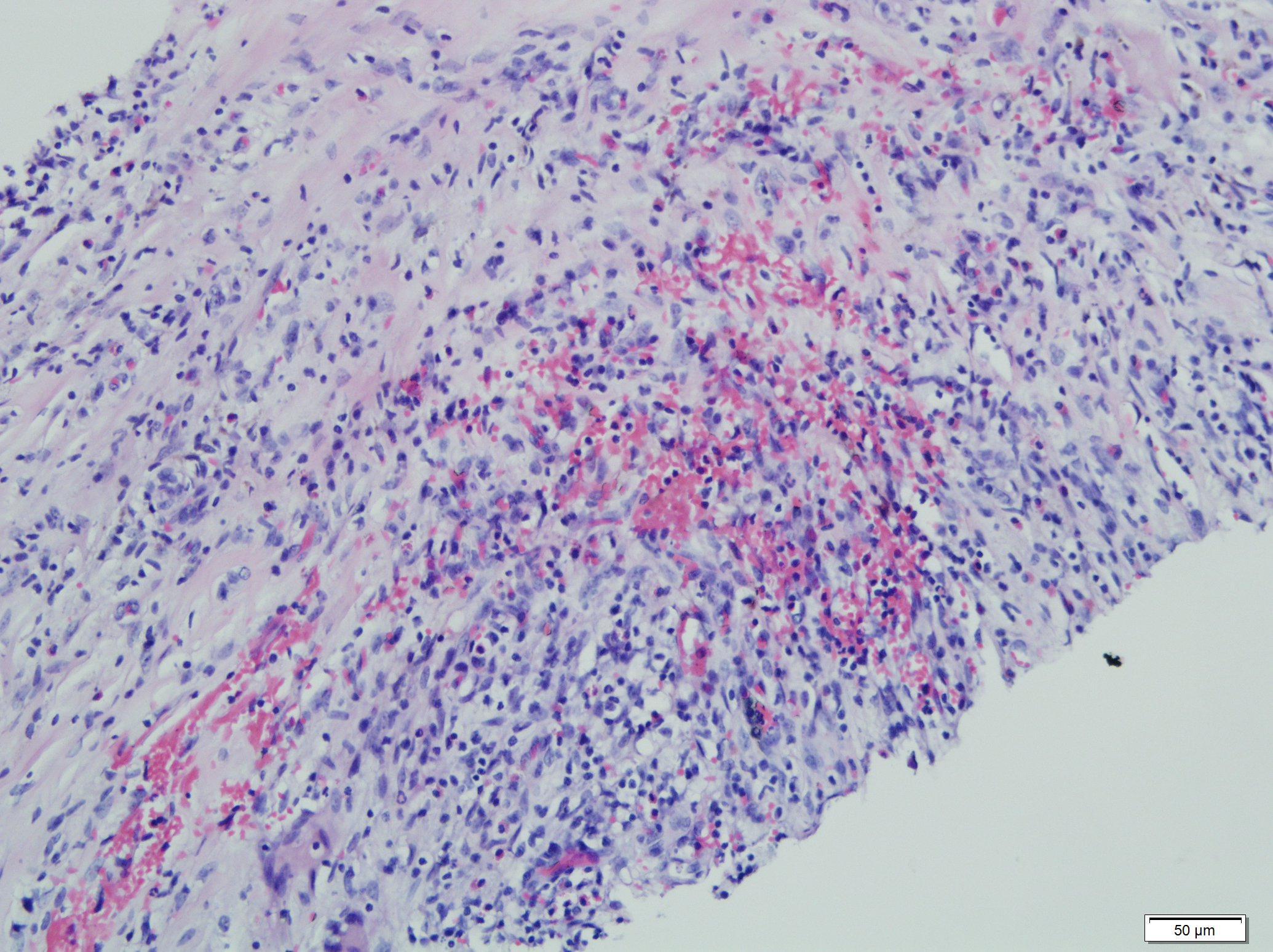

Supplement: S2 File — (ZIP) [file pone.0215499.s002.zip › h&e stain data/3 weeks/3-3 20X-2.jpg]

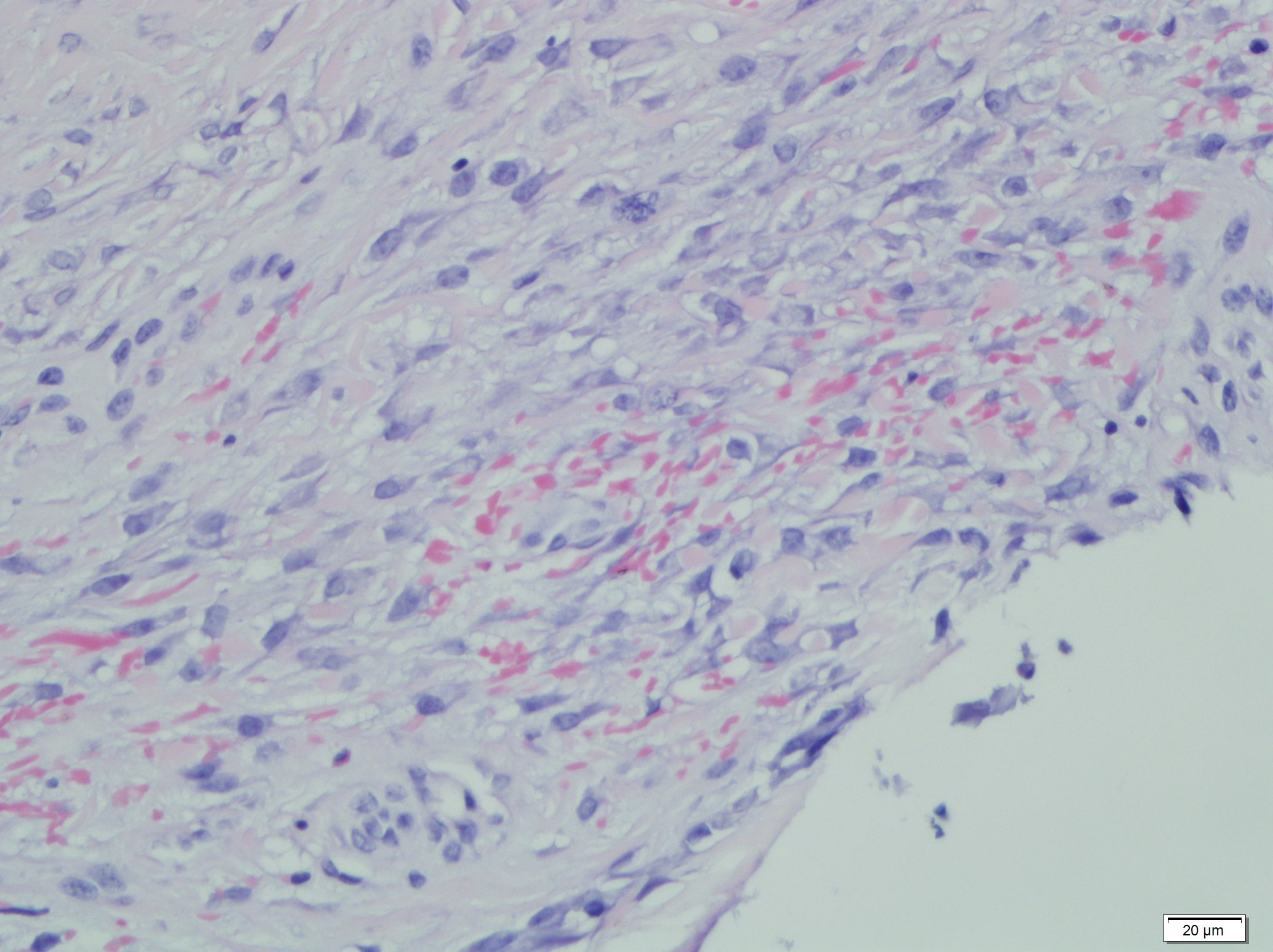

Supplement: S2 File — (ZIP) [file pone.0215499.s002.zip › h&e stain data/3 weeks/3-3 40X-2.jpg]

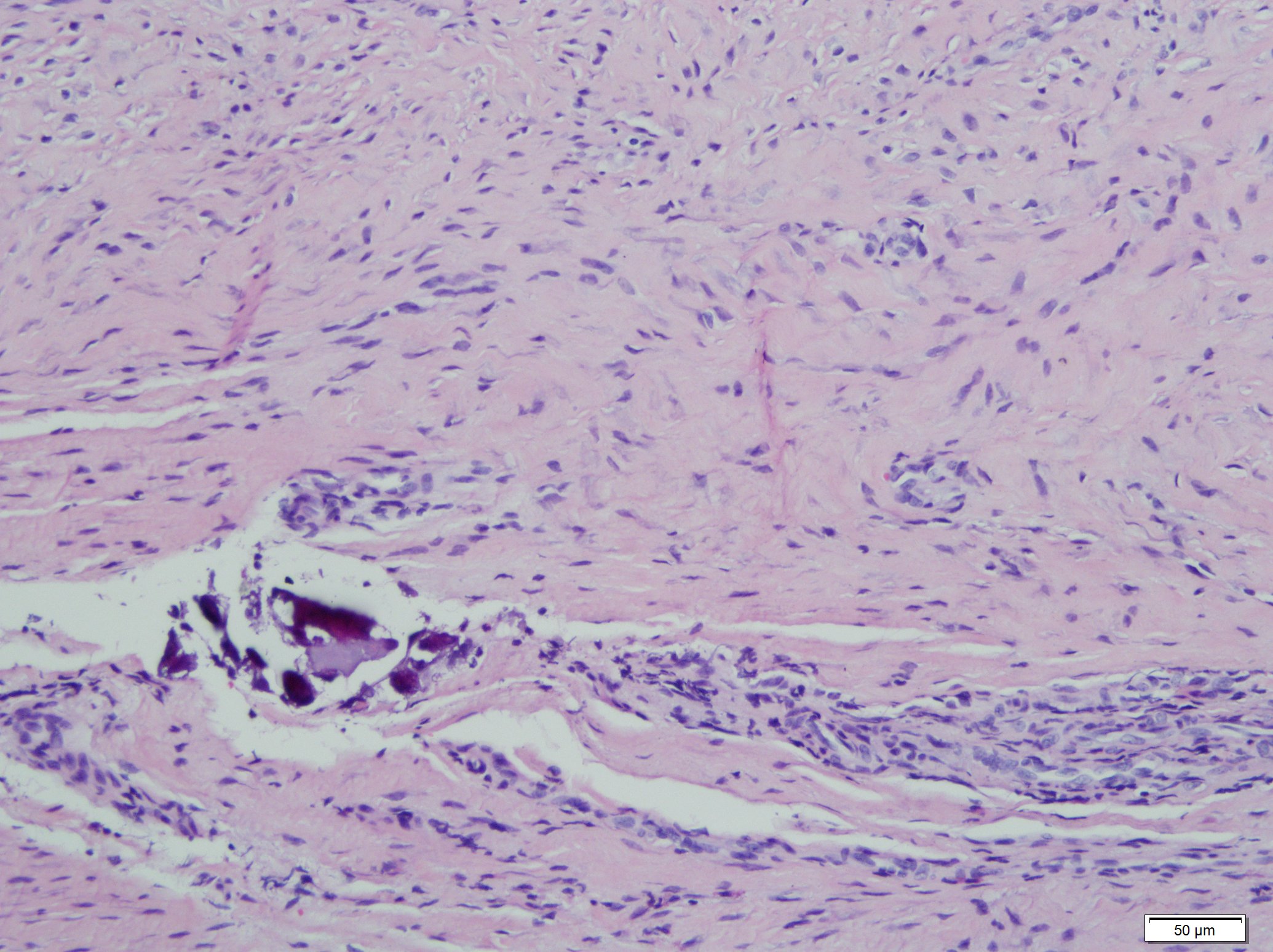

Supplement: S2 File — (ZIP) [file pone.0215499.s002.zip › h&e stain data/3 weeks/3-4 20X-3.jpg]

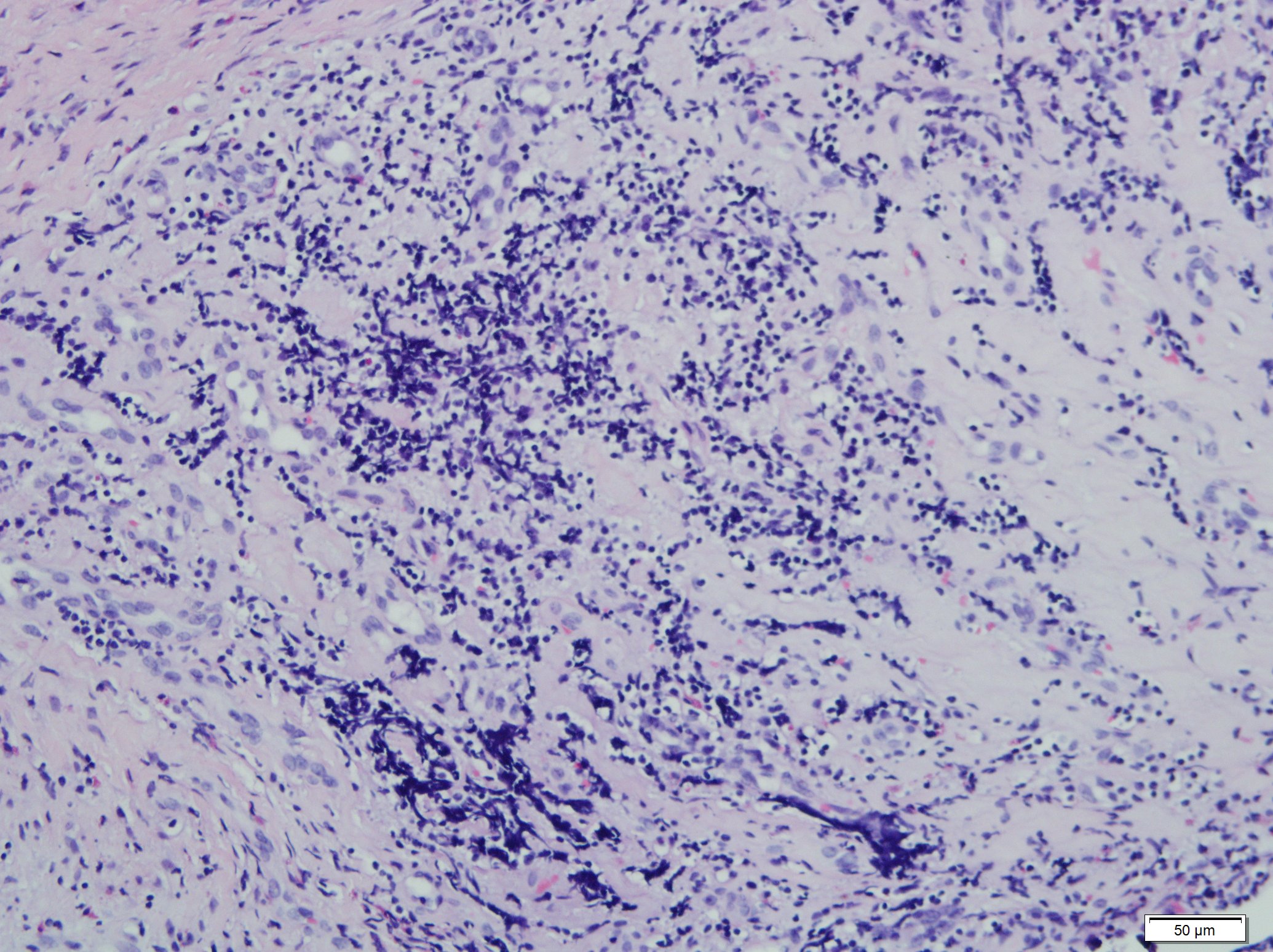

Supplement: S2 File — (ZIP) [file pone.0215499.s002.zip › h&e stain data/3 weeks/3-4 20X.jpg]

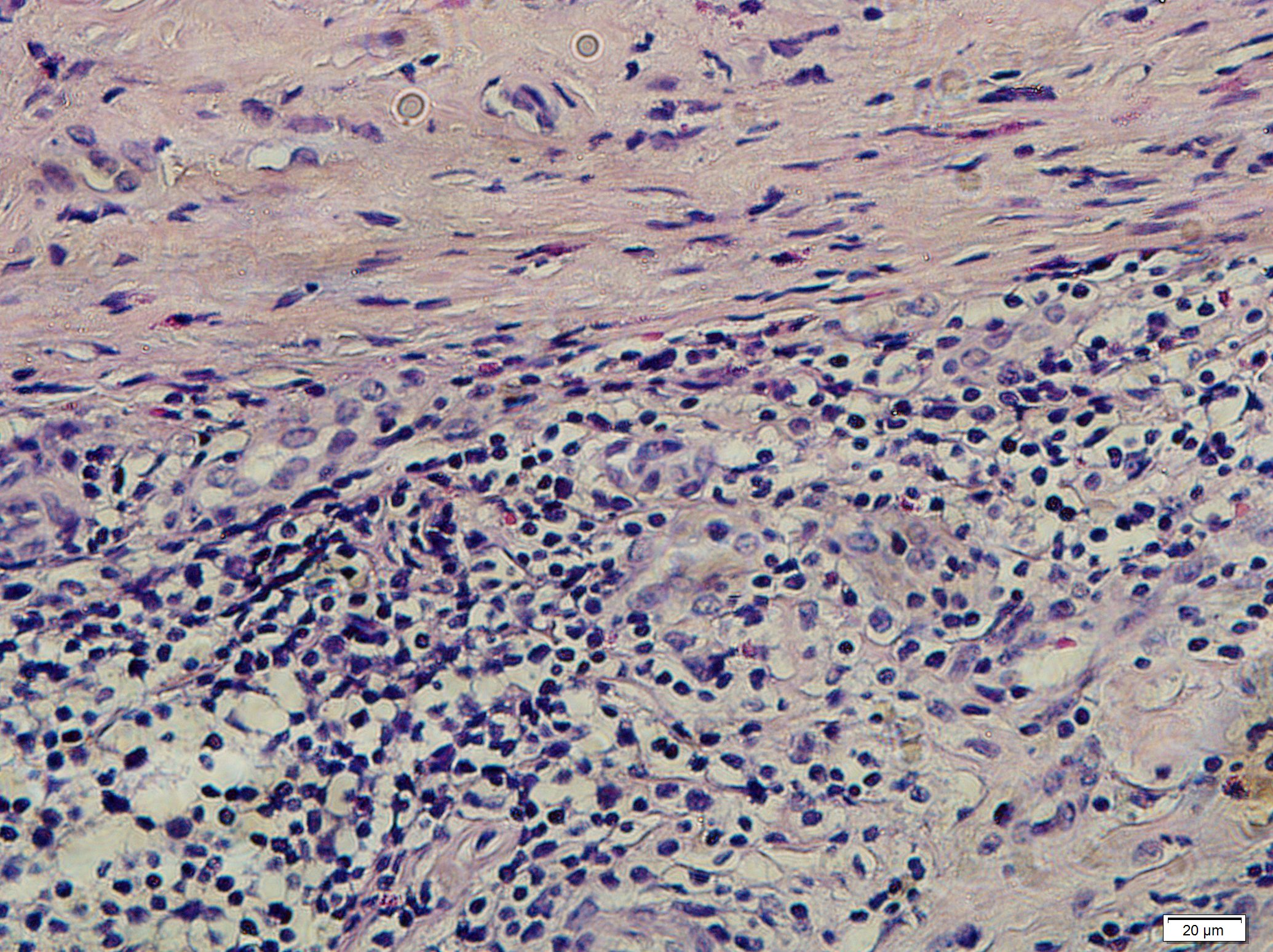

Supplement: S2 File — (ZIP) [file pone.0215499.s002.zip › h&e stain data/3 weeks/3-4 40x (2).jpg]

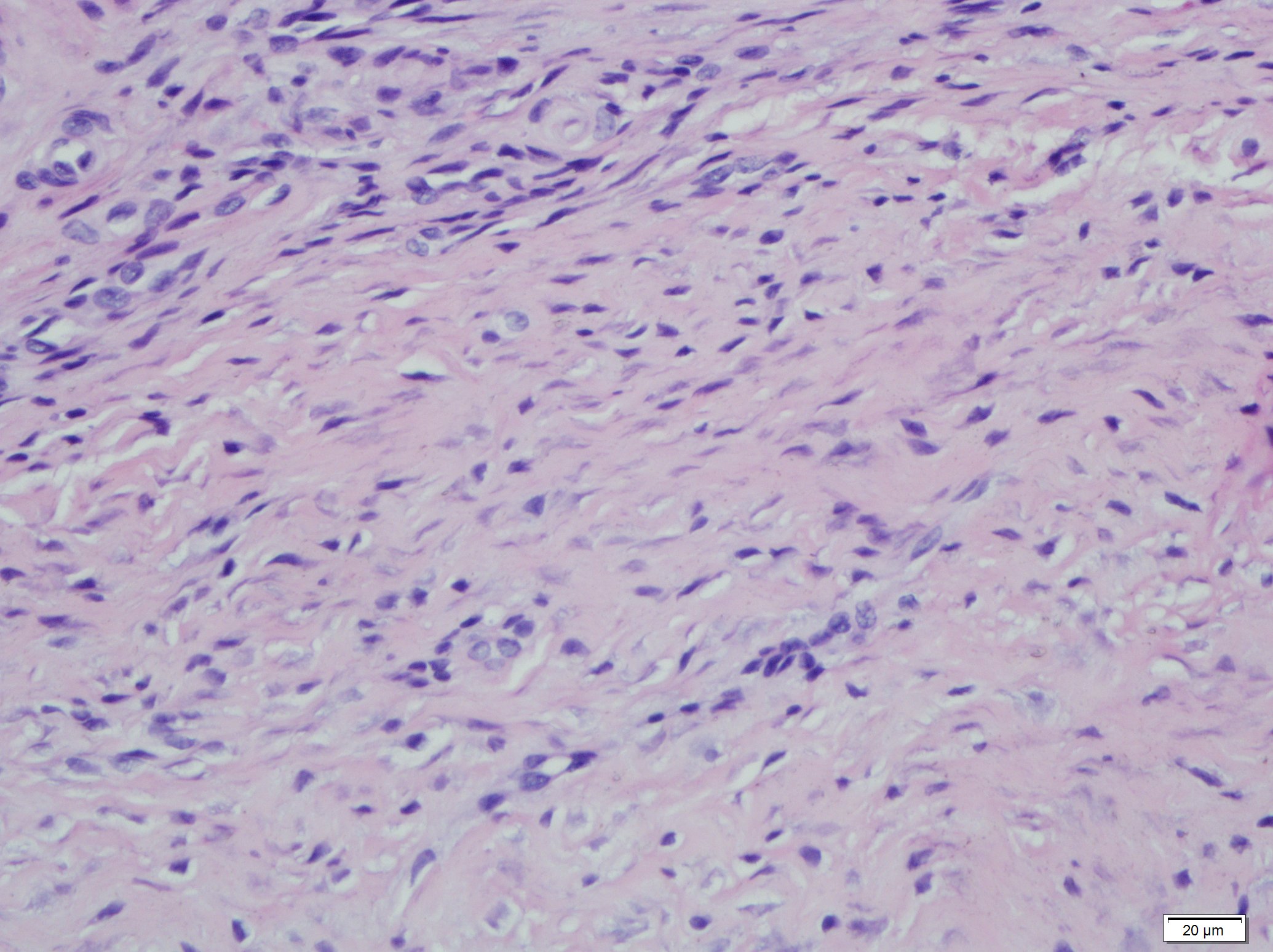

Supplement: S2 File — (ZIP) [file pone.0215499.s002.zip › h&e stain data/3 weeks/3-4 40X.jpg]

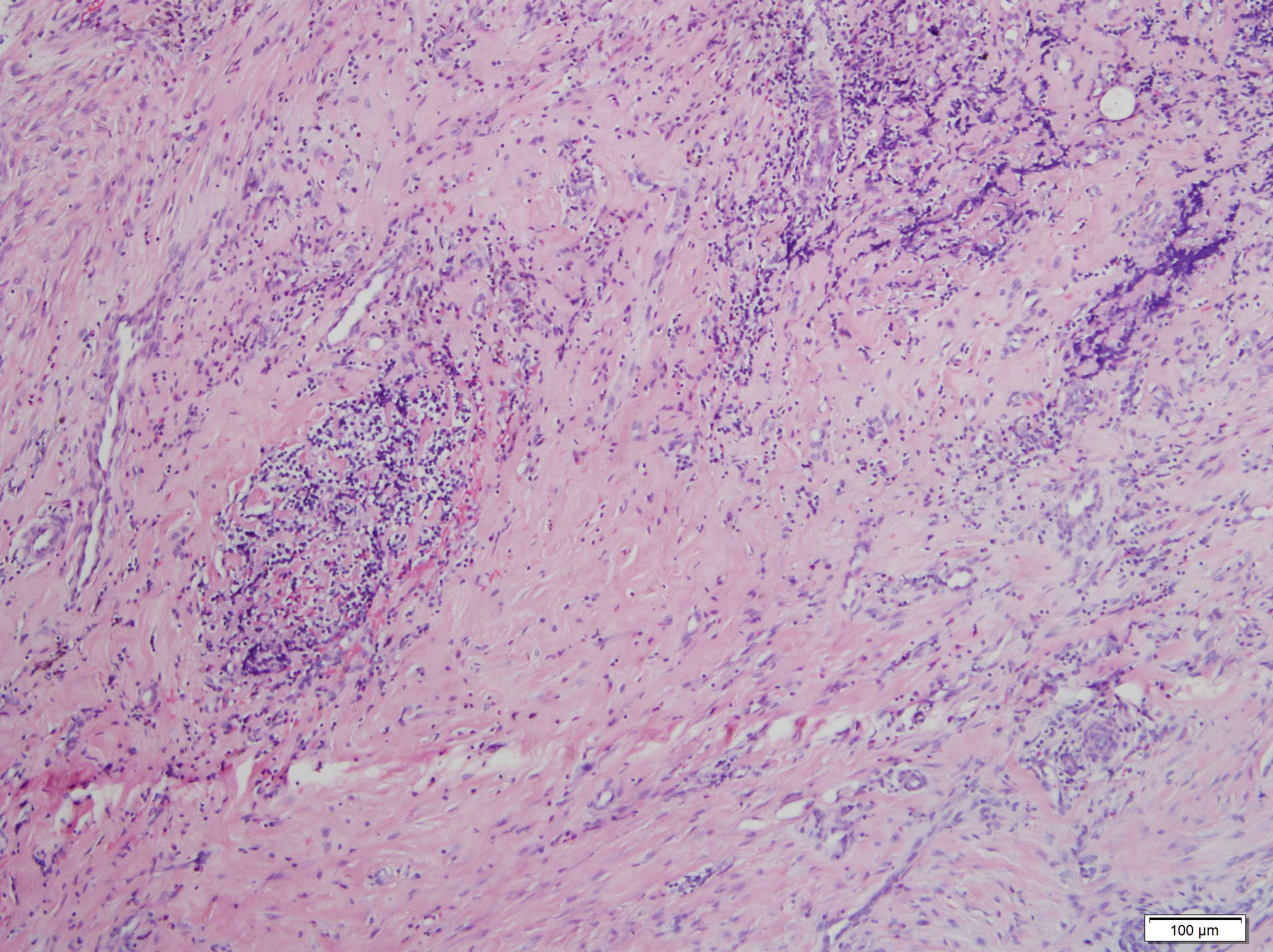

Supplement: S2 File — (ZIP) [file pone.0215499.s002.zip › h&e stain data/3 weeks/3-6 10X-2.jpg]

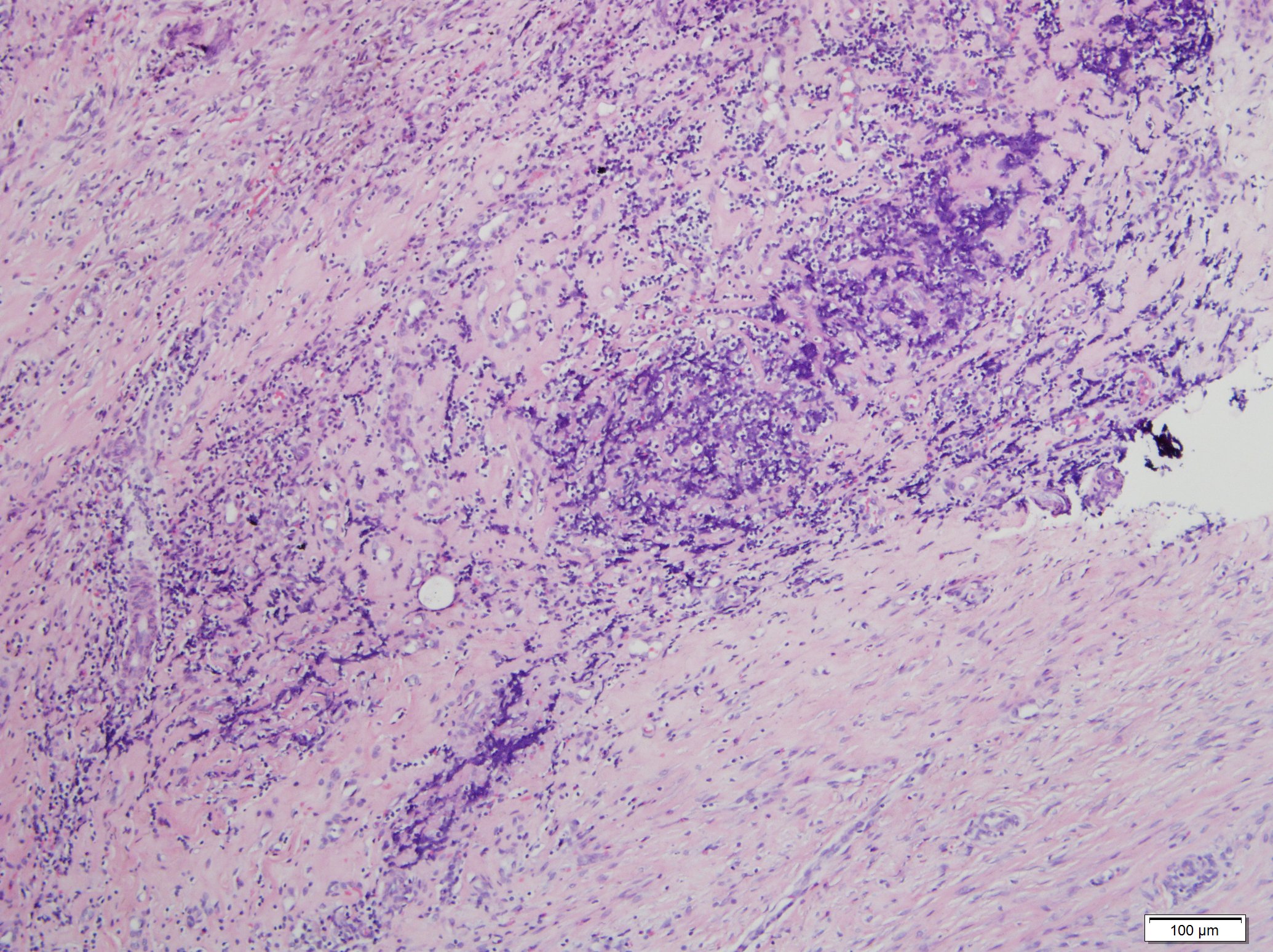

Supplement: S2 File — (ZIP) [file pone.0215499.s002.zip › h&e stain data/3 weeks/3-6 10X-3.jpg]

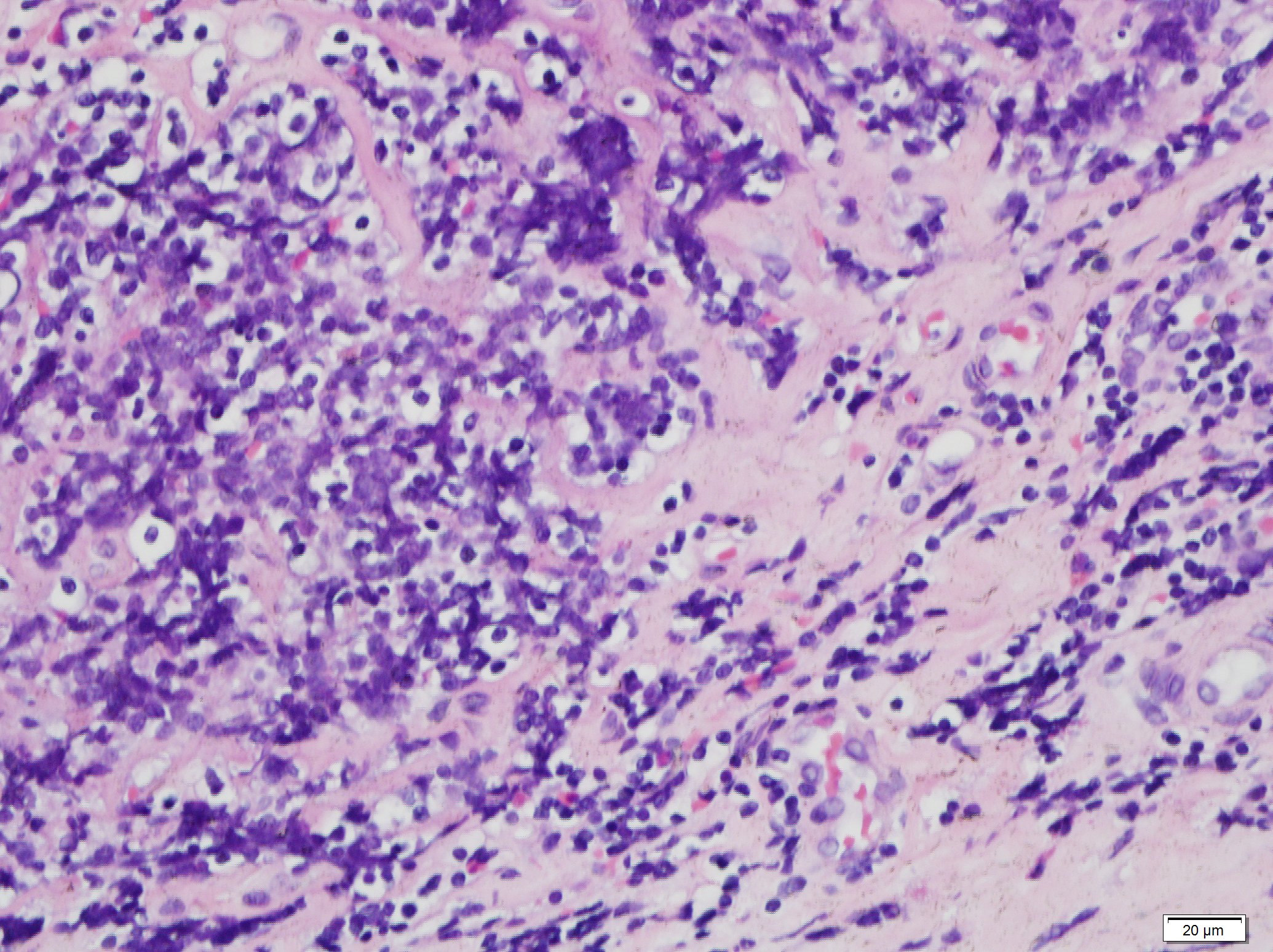

Supplement: S2 File — (ZIP) [file pone.0215499.s002.zip › h&e stain data/3 weeks/3-6 40X-2.jpg]

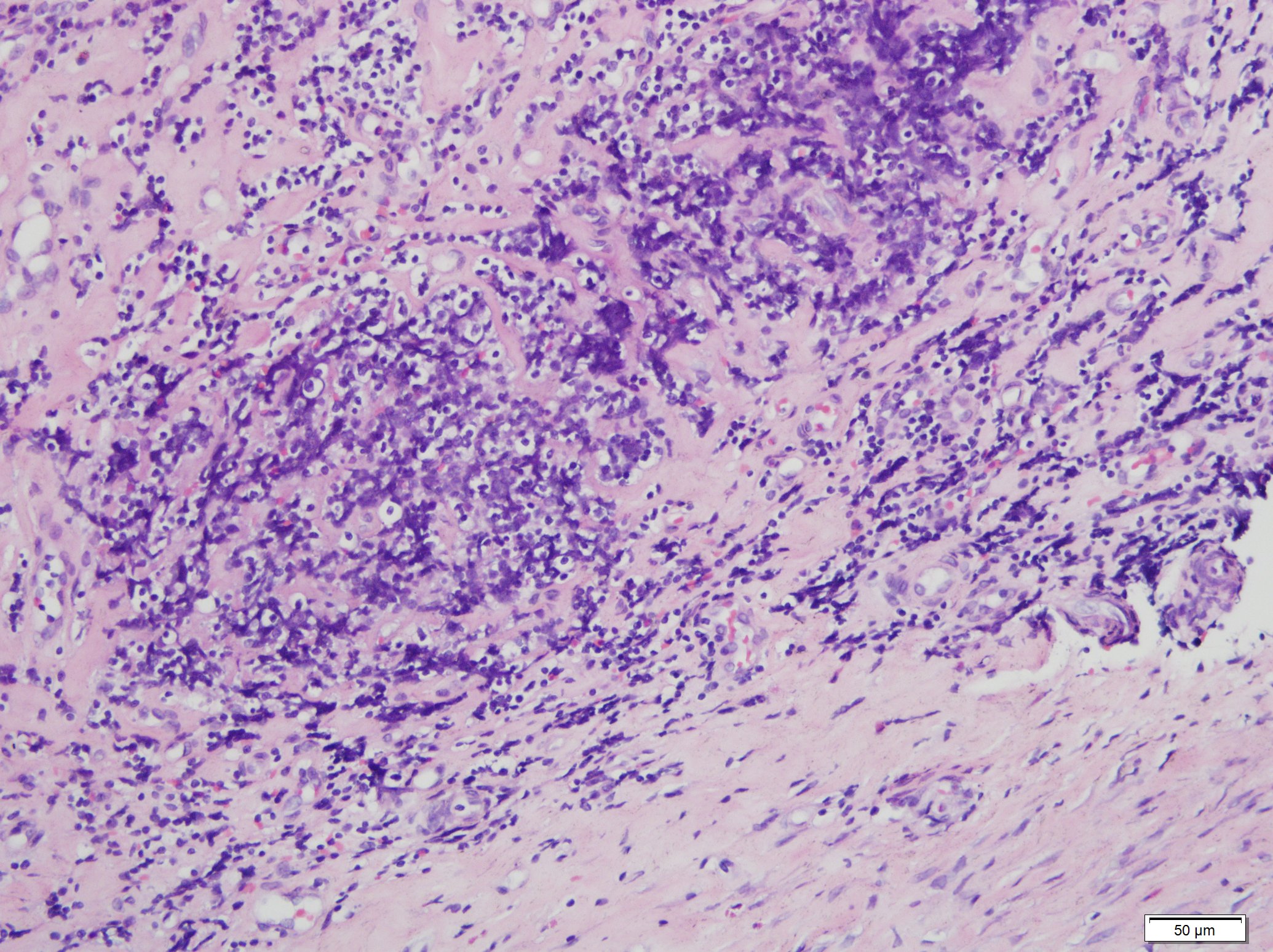

Supplement: S2 File — (ZIP) [file pone.0215499.s002.zip › h&e stain data/3 weeks/3-6 40X.jpg]

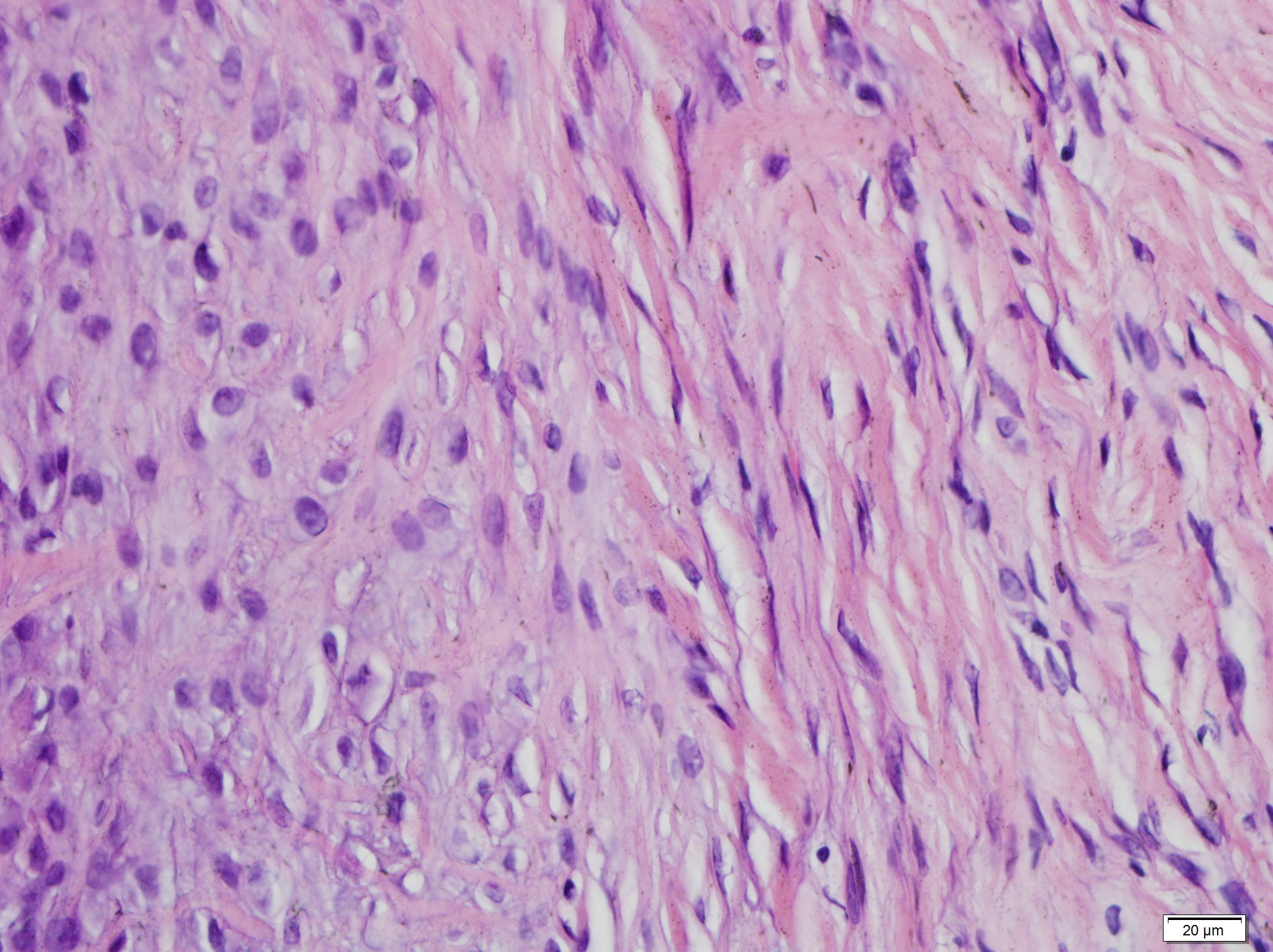

Supplement: S2 File — (ZIP) [file pone.0215499.s002.zip › h&e stain data/3 weeks/3-7 40X-3.jpg]

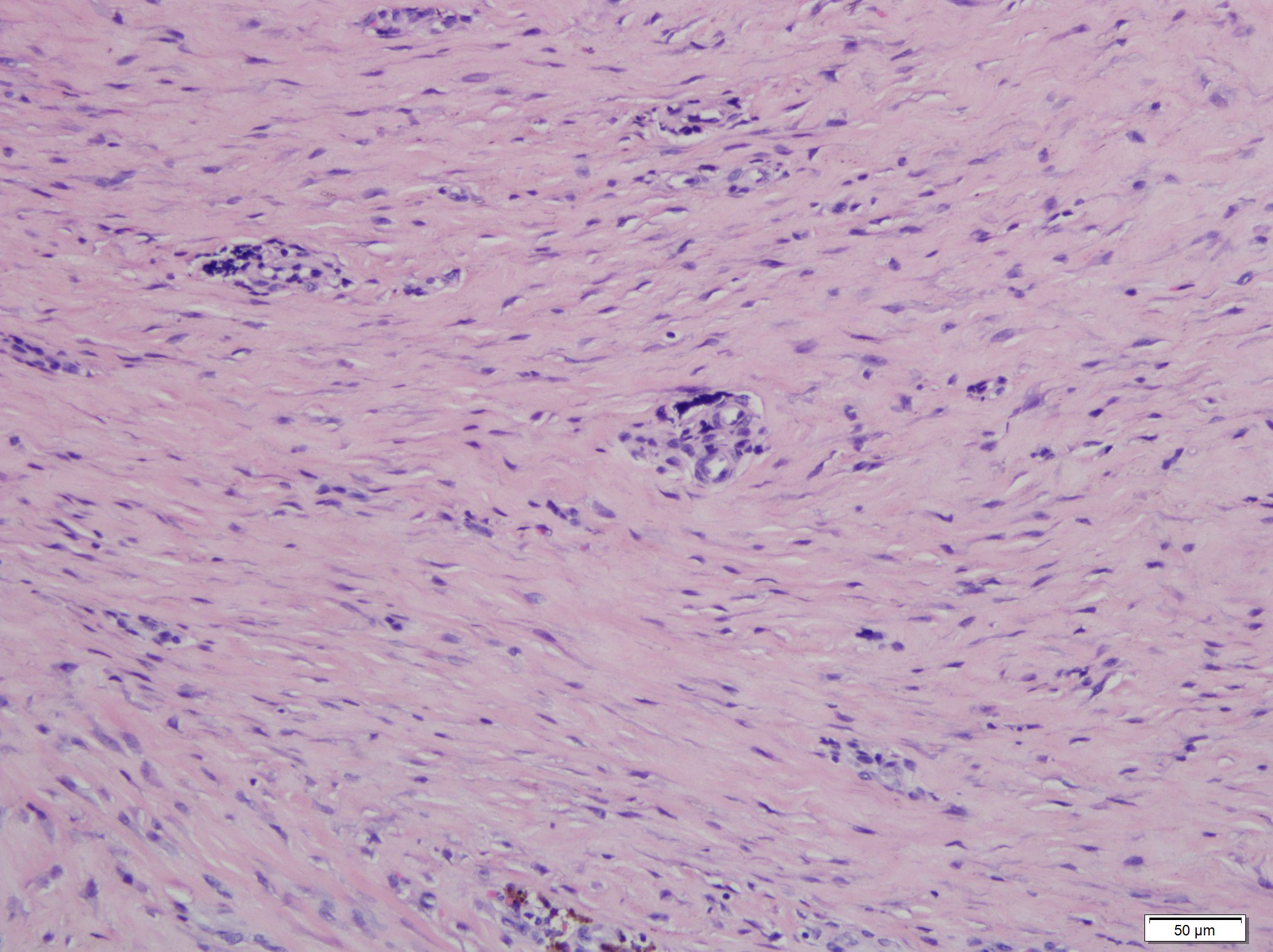

Supplement: S2 File — (ZIP) [file pone.0215499.s002.zip › h&e stain data/4 weeks/4-4 20X.jpg]

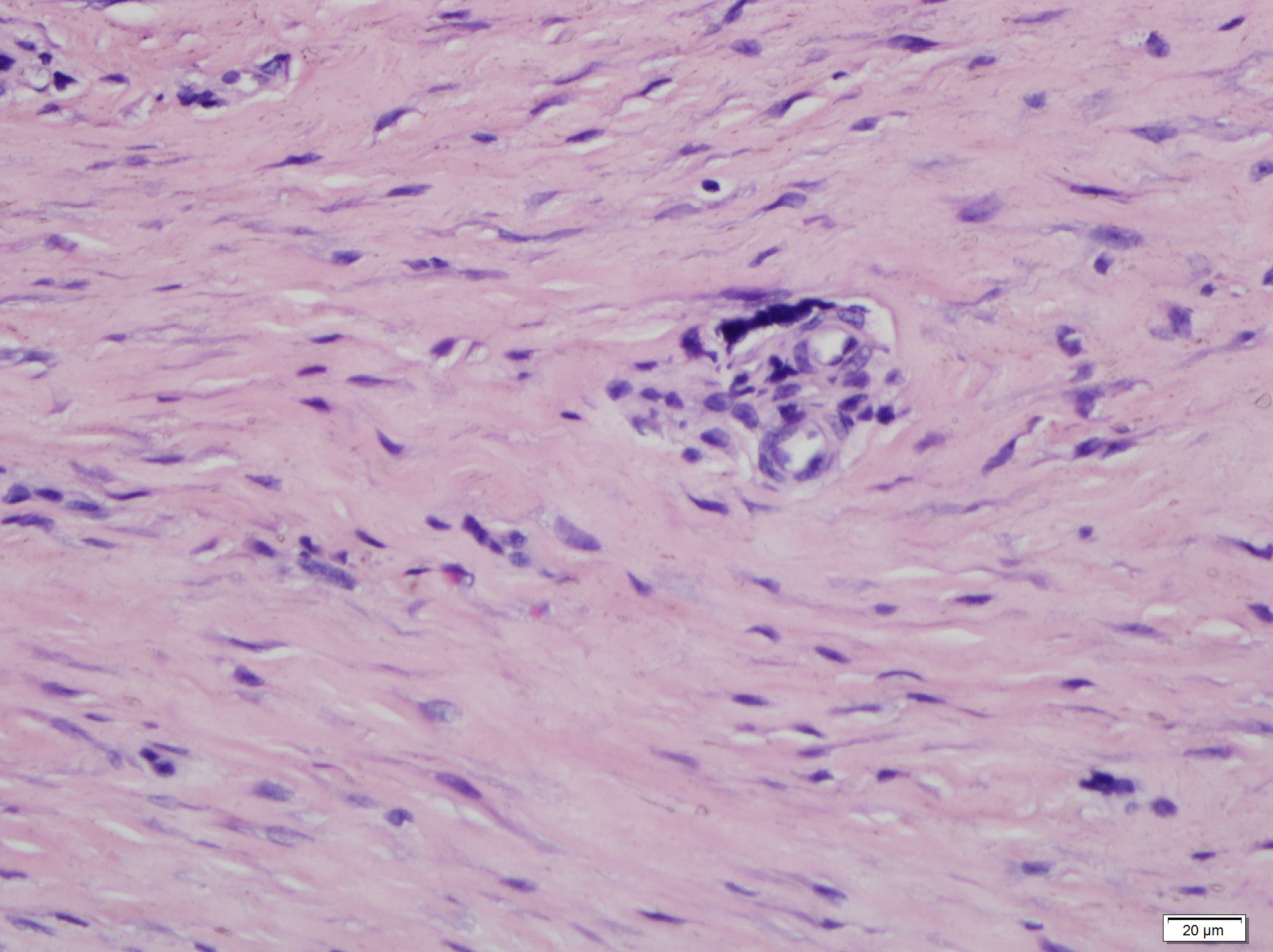

Supplement: S2 File — (ZIP) [file pone.0215499.s002.zip › h&e stain data/4 weeks/4-4 40X.jpg]

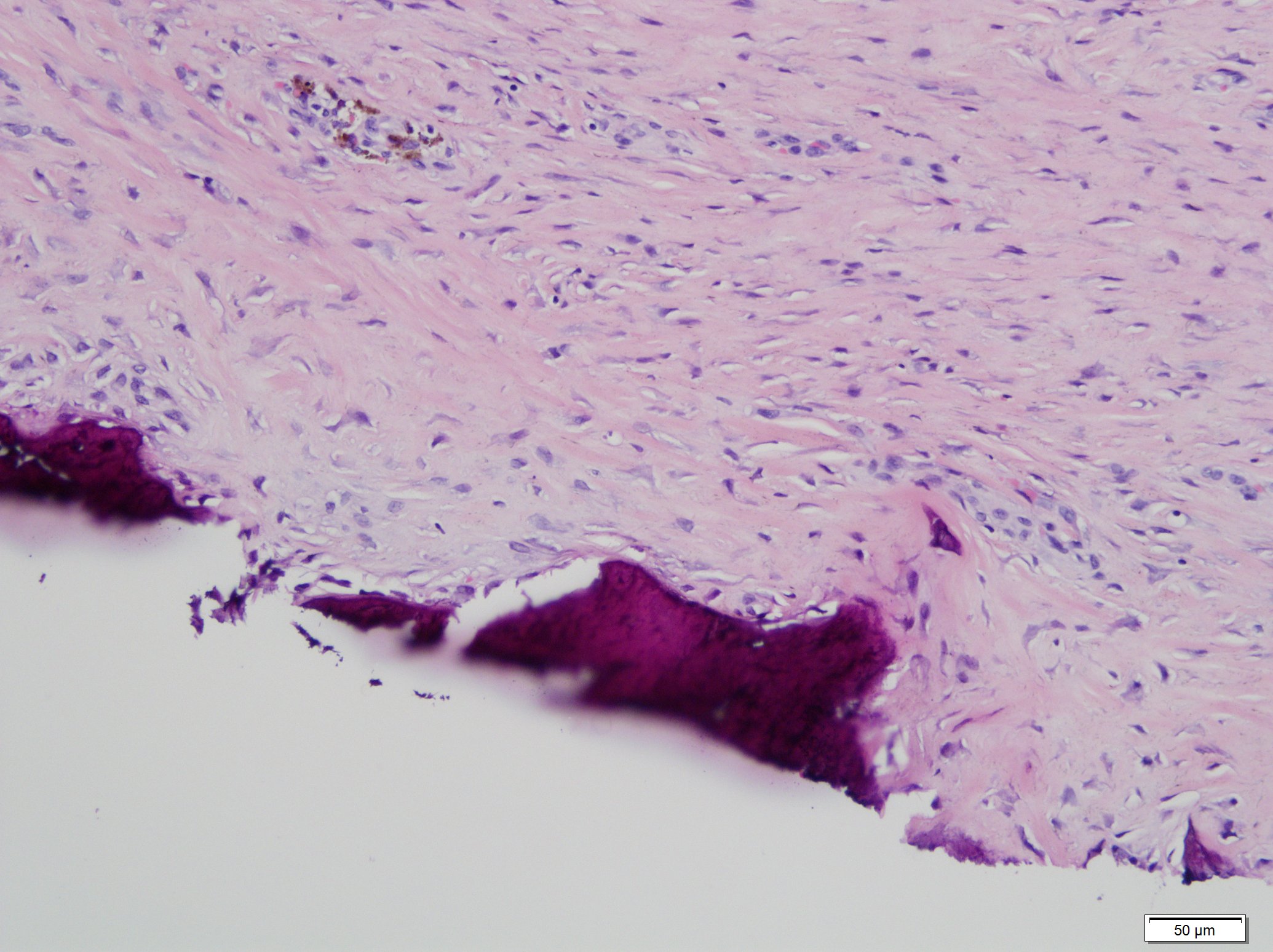

Supplement: S2 File — (ZIP) [file pone.0215499.s002.zip › h&e stain data/4 weeks/4-5x 20X-3.jpg]

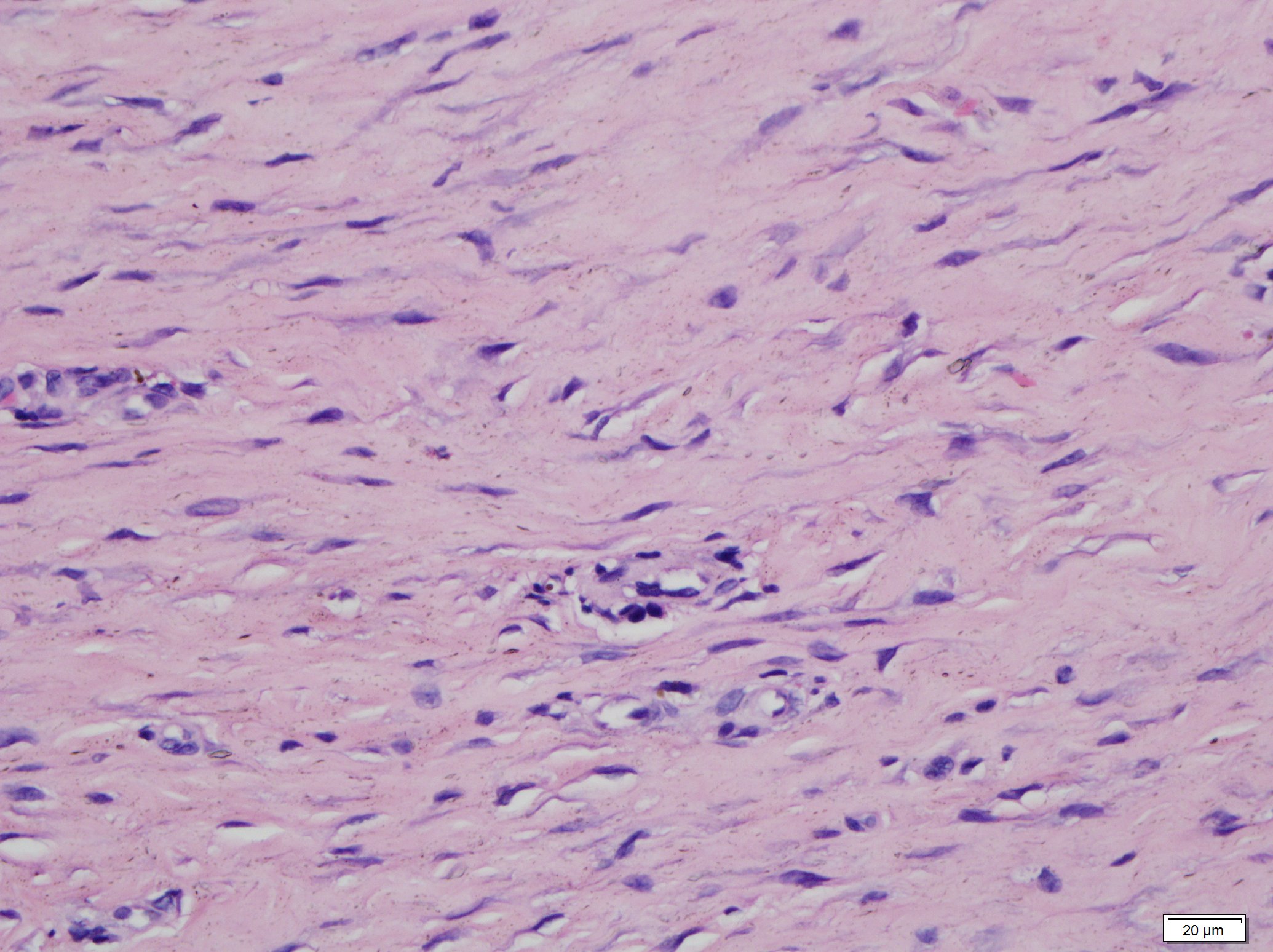

Supplement: S2 File — (ZIP) [file pone.0215499.s002.zip › h&e stain data/4 weeks/4-5x 40X-3.jpg]

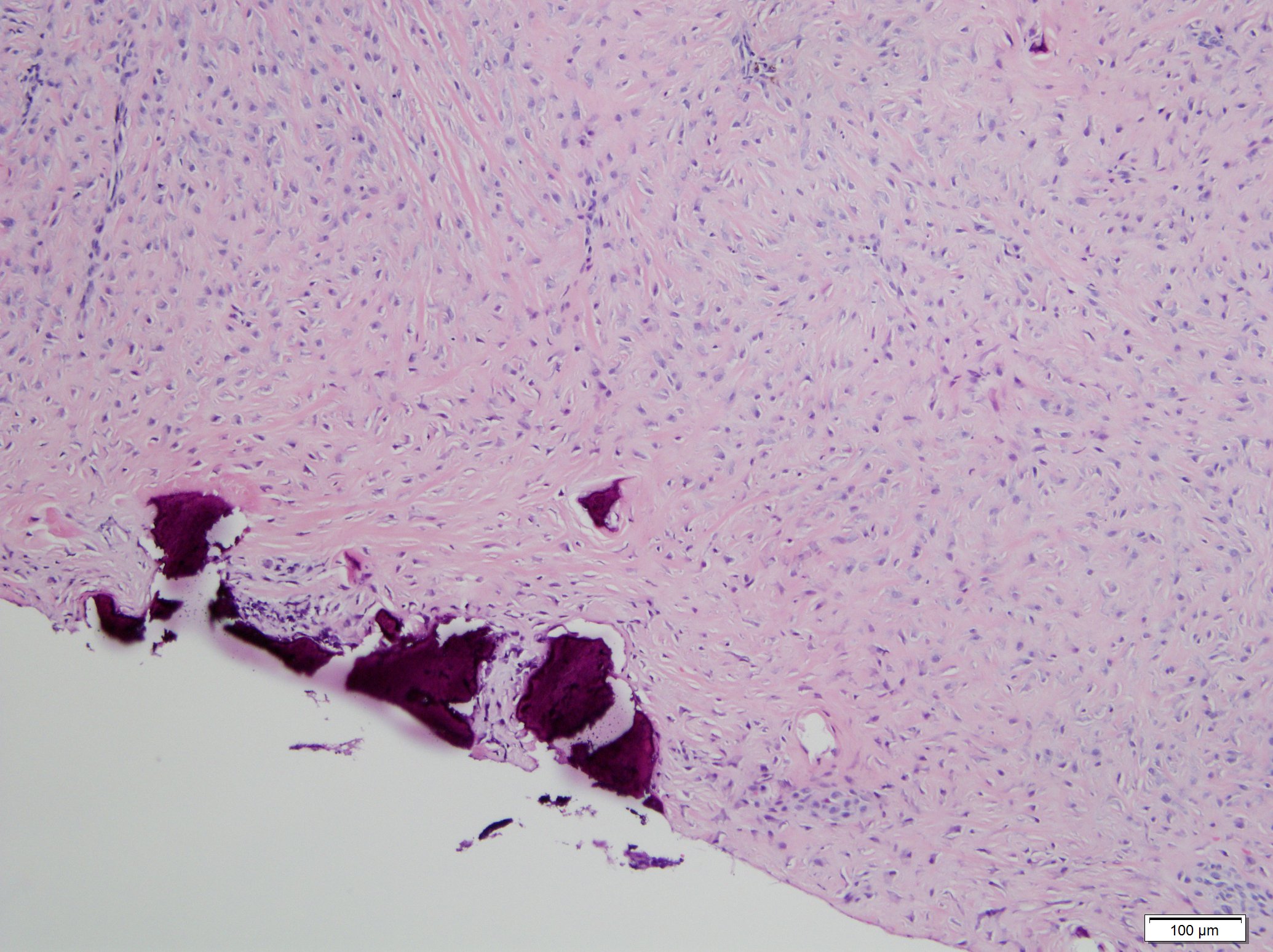

Supplement: S2 File — (ZIP) [file pone.0215499.s002.zip › h&e stain data/4 weeks/4-5x 10X-2.jpg]

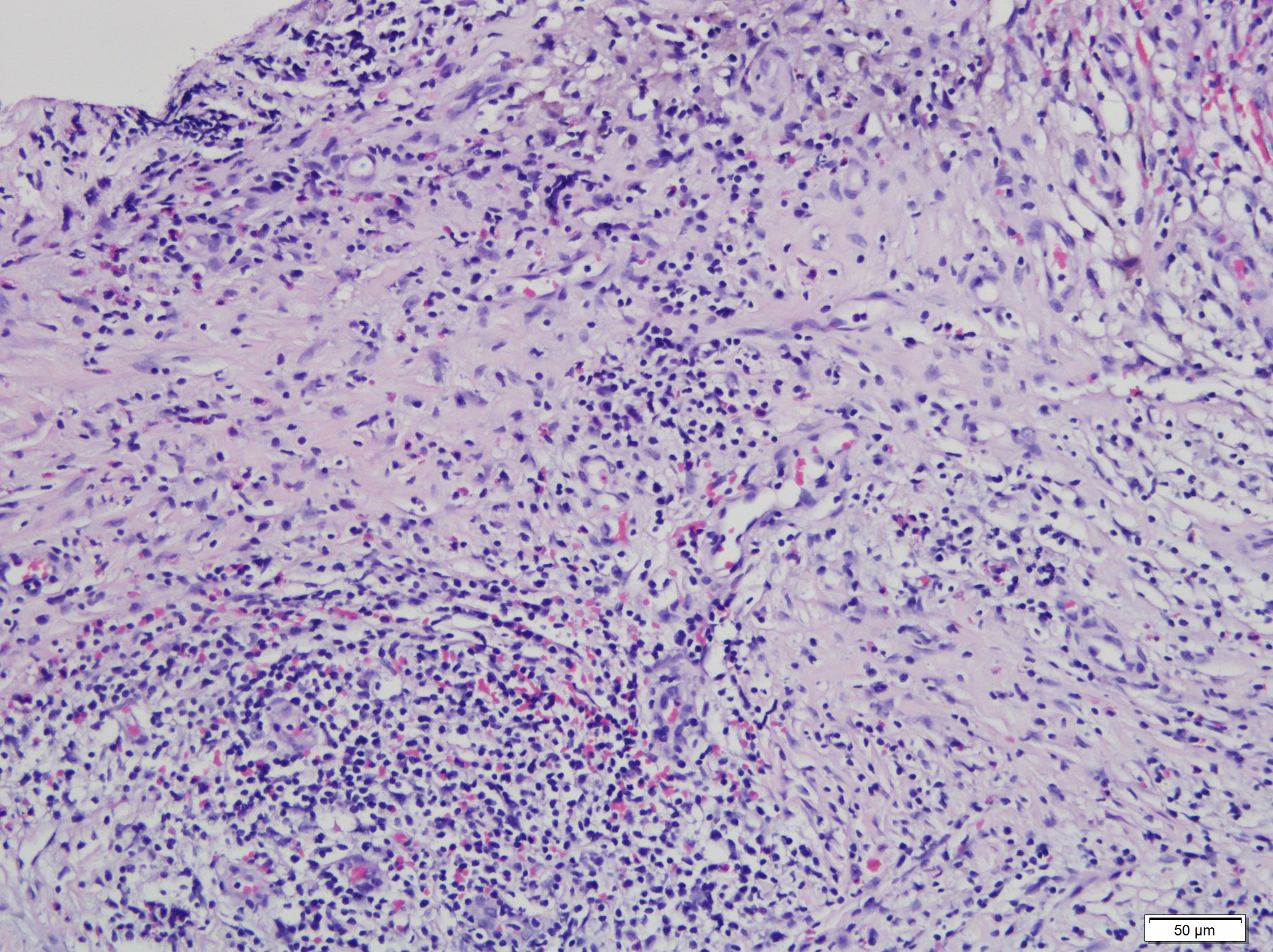

Supplement: S2 File — (ZIP) [file pone.0215499.s002.zip › h&e stain data/4 weeks/4-6x 20X-2.jpg]

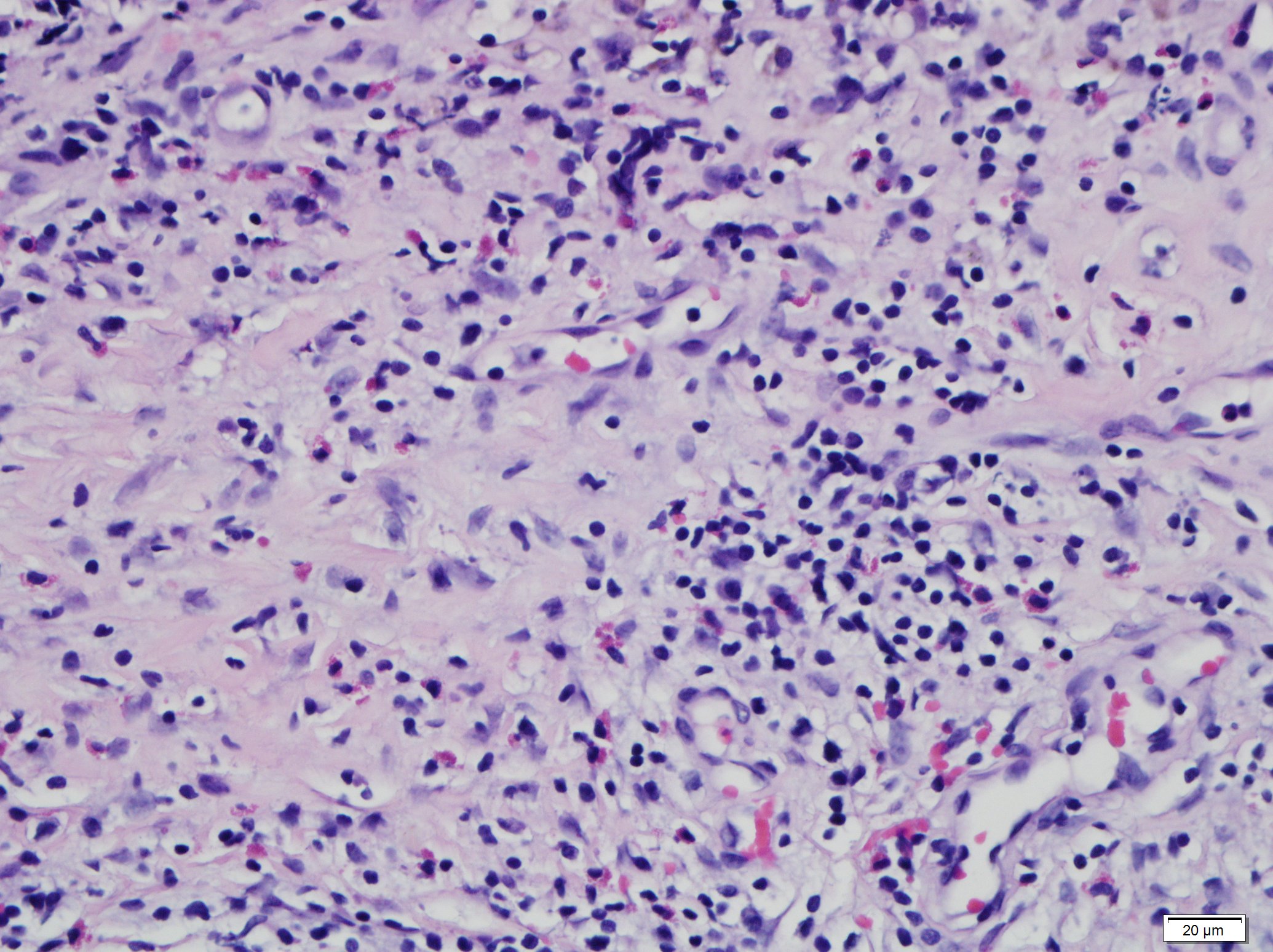

Supplement: S2 File — (ZIP) [file pone.0215499.s002.zip › h&e stain data/4 weeks/4-6x 40X-3.jpg]

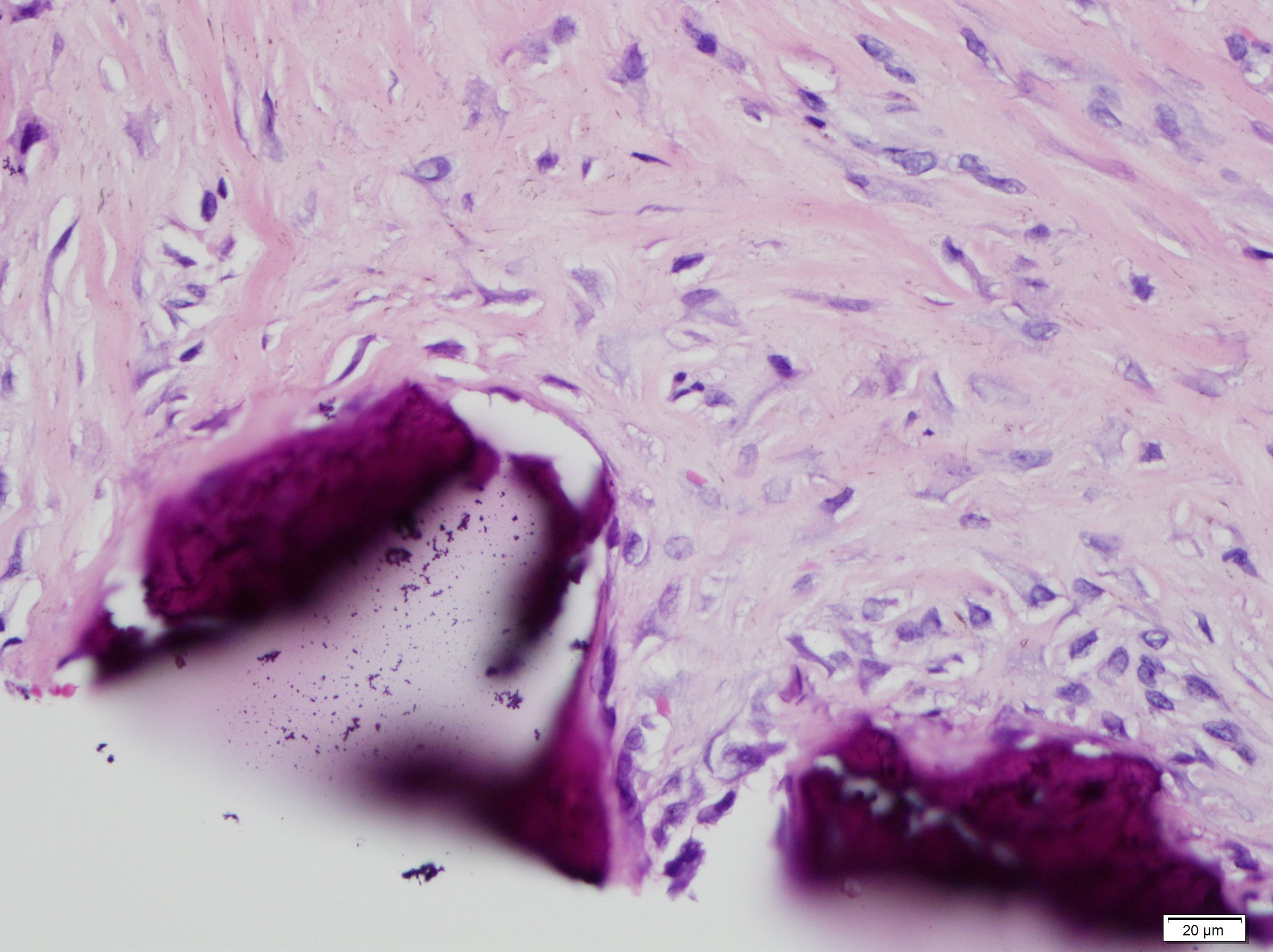

Supplement: S2 File — (ZIP) [file pone.0215499.s002.zip › h&e stain data/4 weeks/4-7 x 40X-2.jpg]

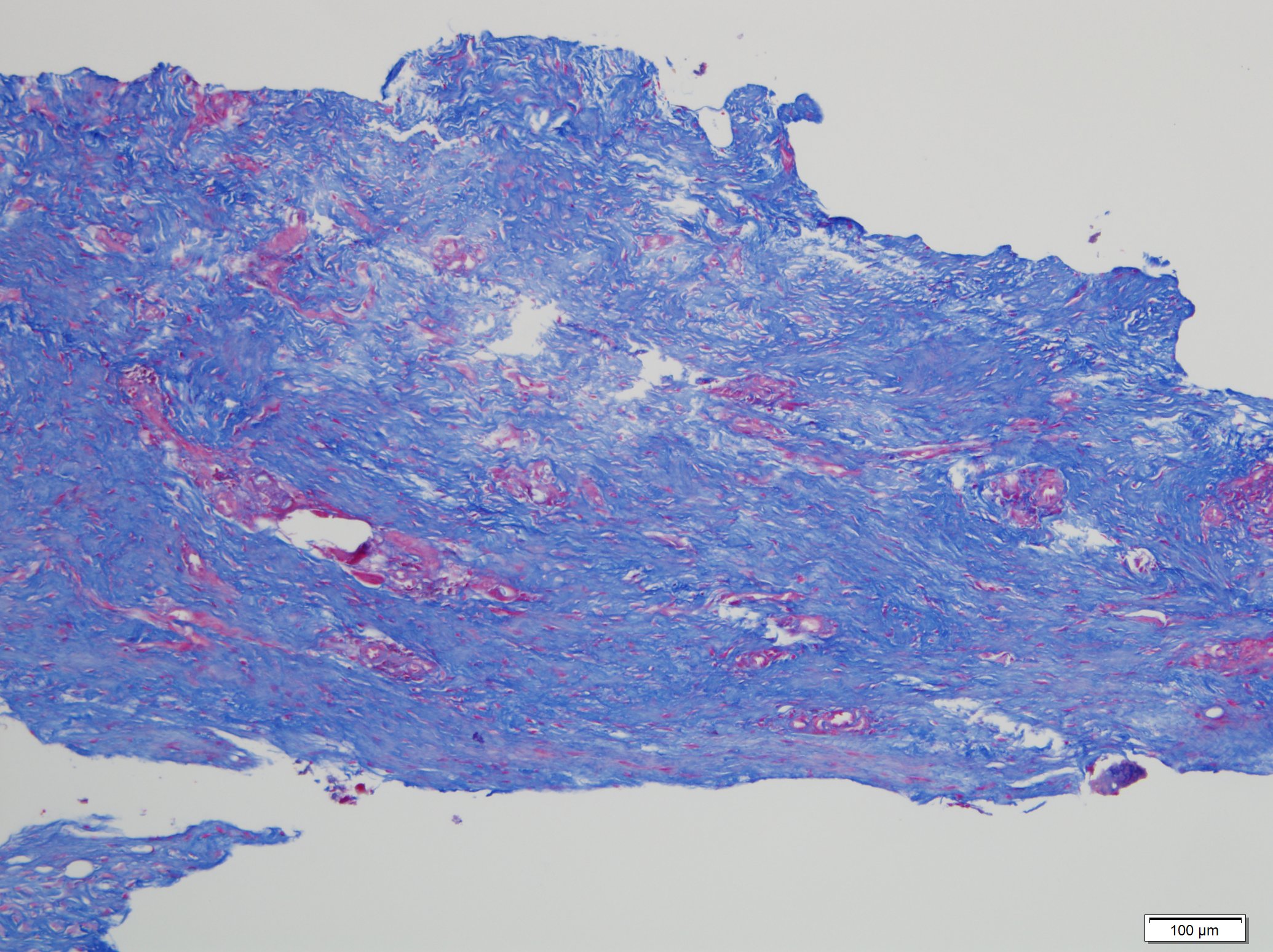

Supplement: S3 File — (ZIP) [file pone.0215499.s003.zip › masson's trichrome/1 week/2-1 10x-2.jpg]

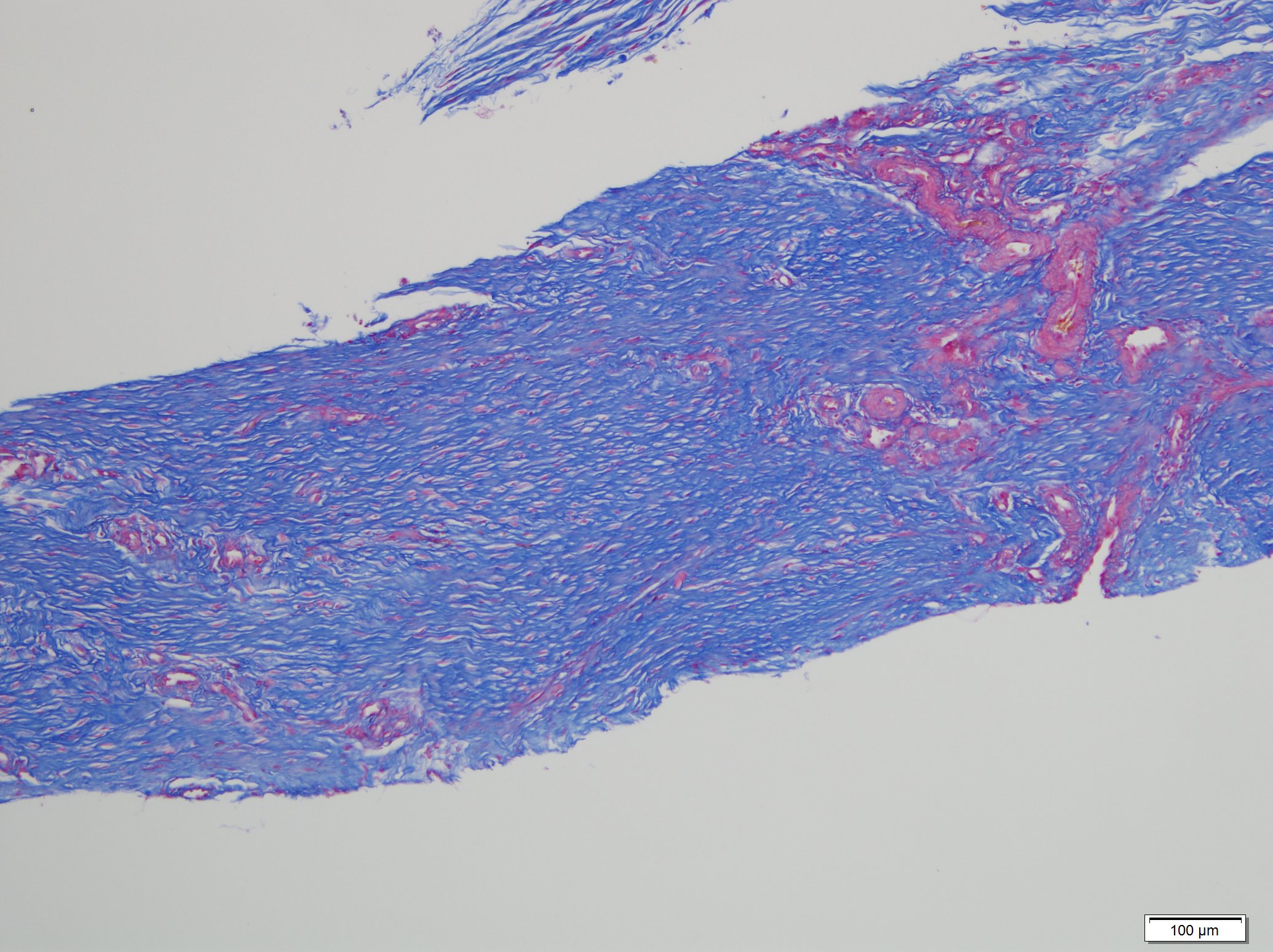

Supplement: S3 File — (ZIP) [file pone.0215499.s003.zip › masson's trichrome/1 week/2-1 10x-3.jpg]

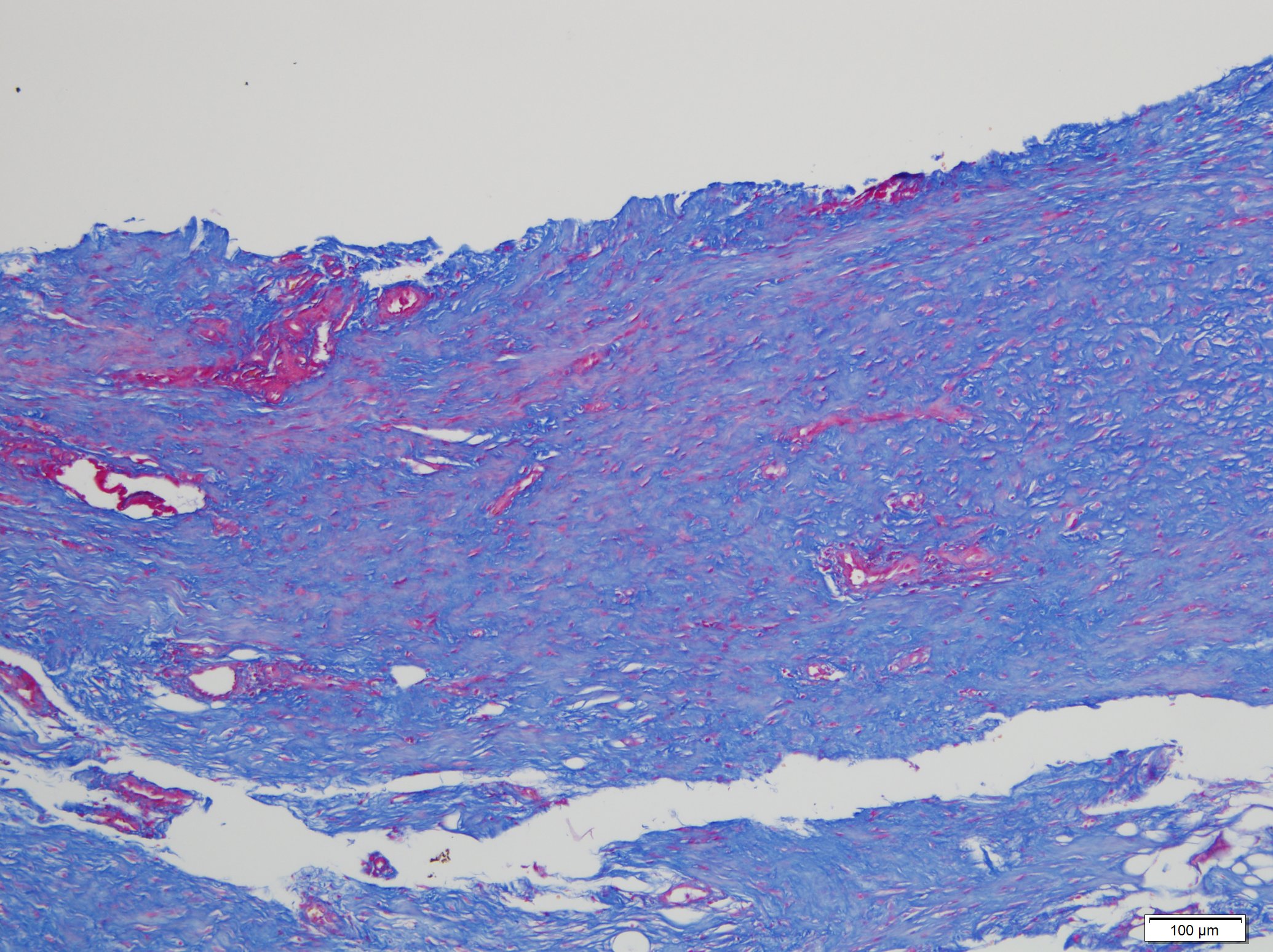

Supplement: S3 File — (ZIP) [file pone.0215499.s003.zip › masson's trichrome/1 week/2-1 10x.jpg]

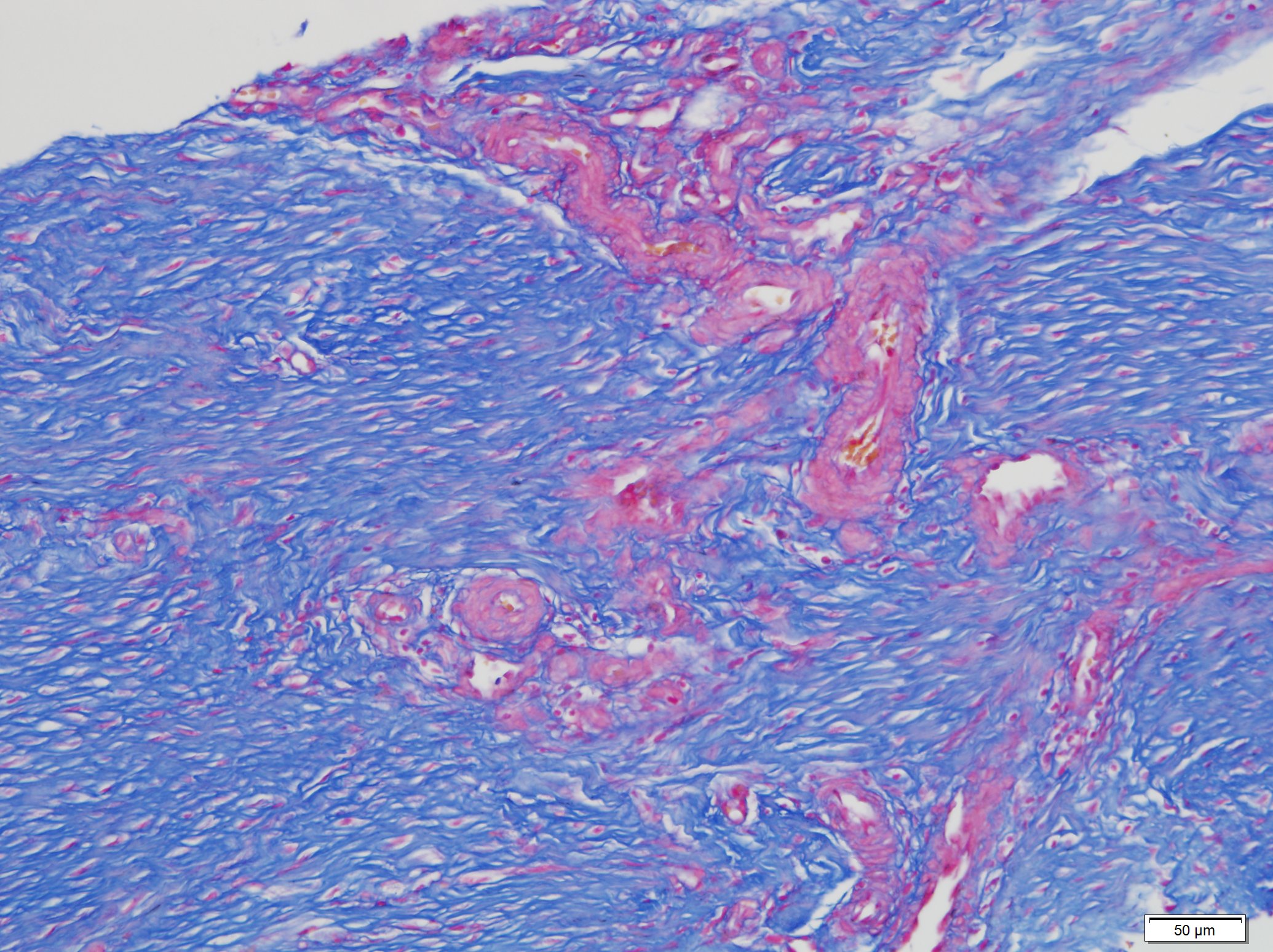

Supplement: S3 File — (ZIP) [file pone.0215499.s003.zip › masson's trichrome/1 week/2-1 20x.jpg]

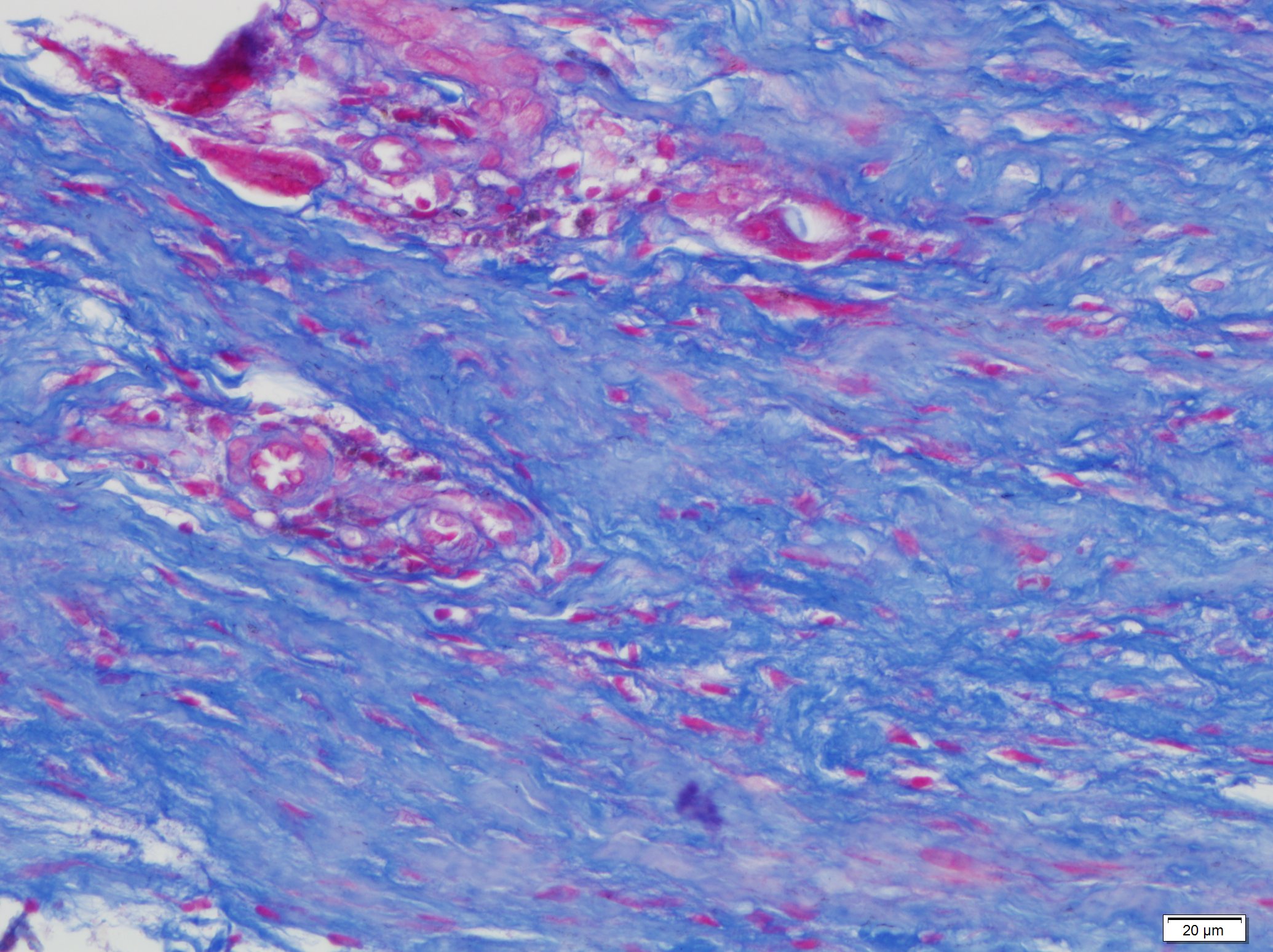

Supplement: S3 File — (ZIP) [file pone.0215499.s003.zip › masson's trichrome/1 week/2-1 40x-2.jpg]

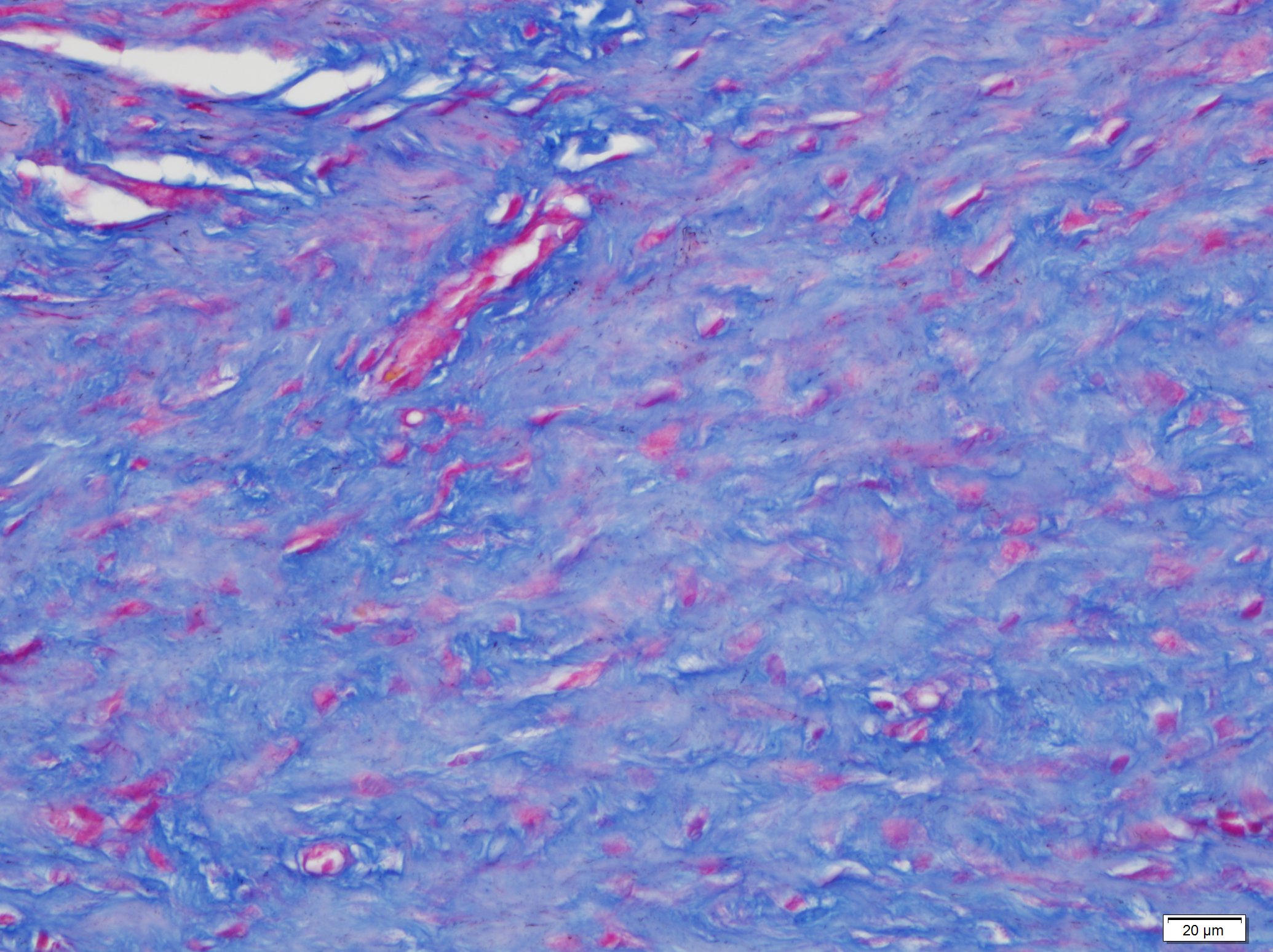

Supplement: S3 File — (ZIP) [file pone.0215499.s003.zip › masson's trichrome/1 week/2-1 40x-3.jpg]

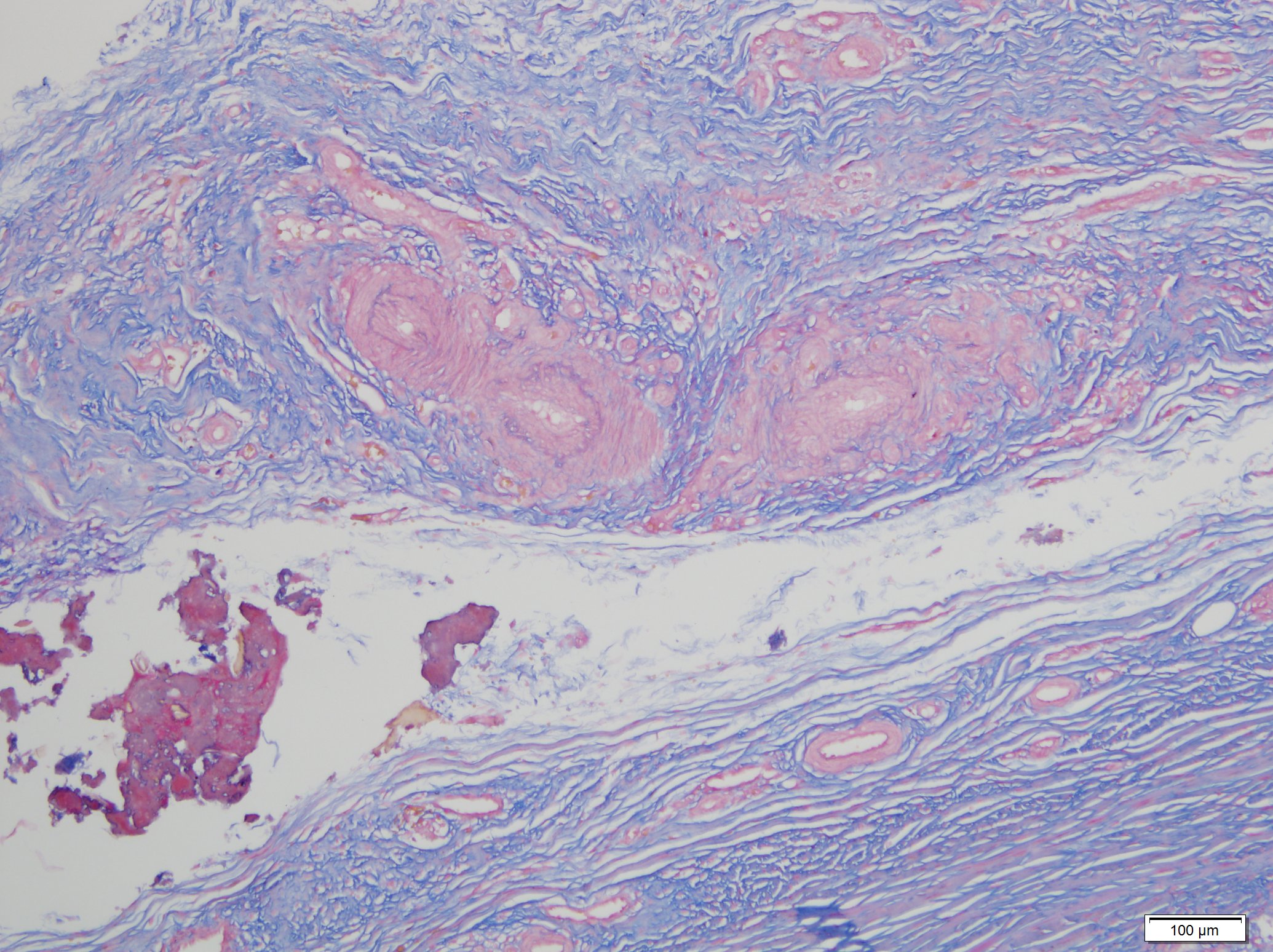

Supplement: S3 File — (ZIP) [file pone.0215499.s003.zip › masson's trichrome/1 week/2-2 10x-2.jpg]

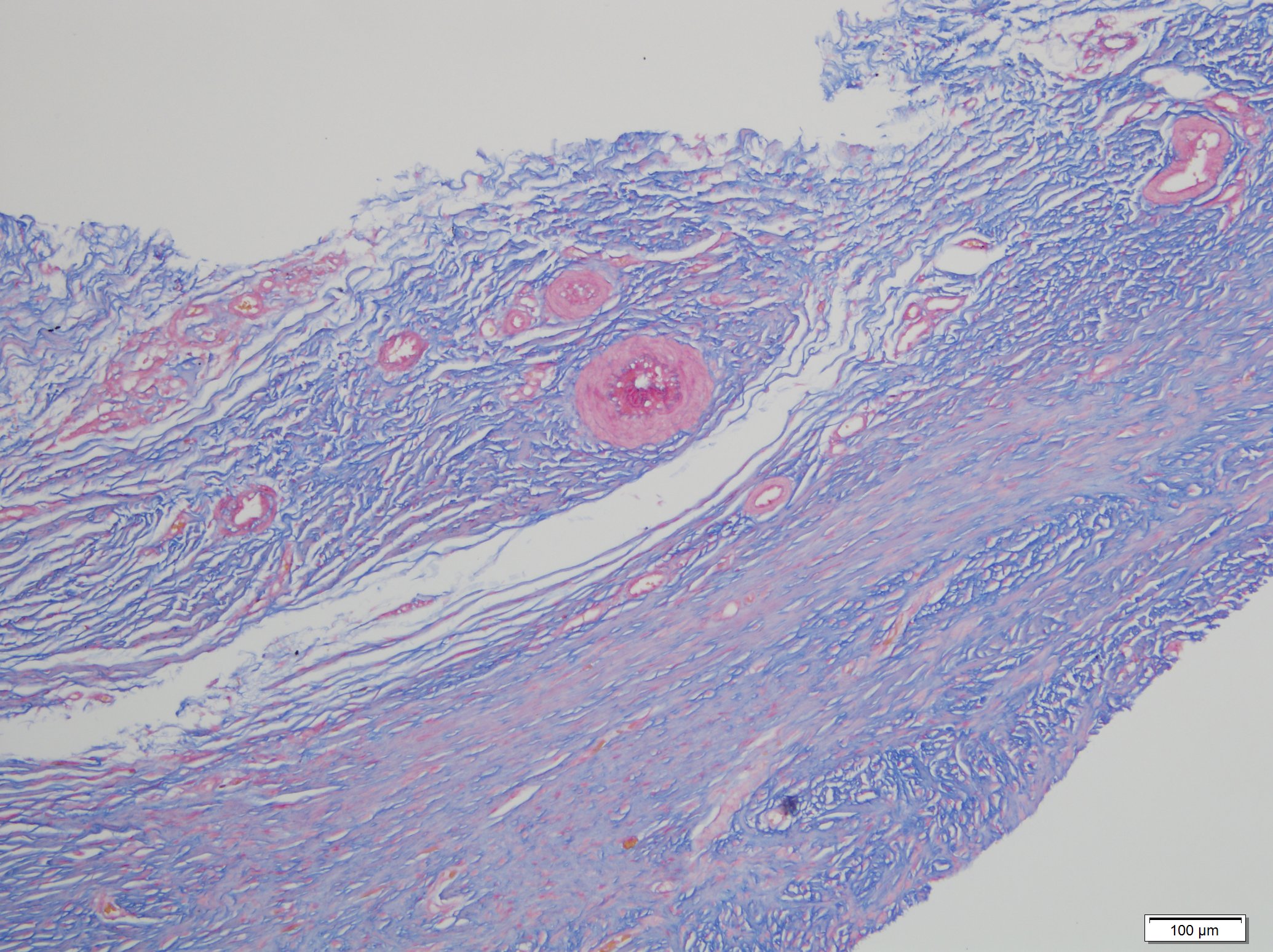

Supplement: S3 File — (ZIP) [file pone.0215499.s003.zip › masson's trichrome/1 week/2-2 10x-3.jpg]

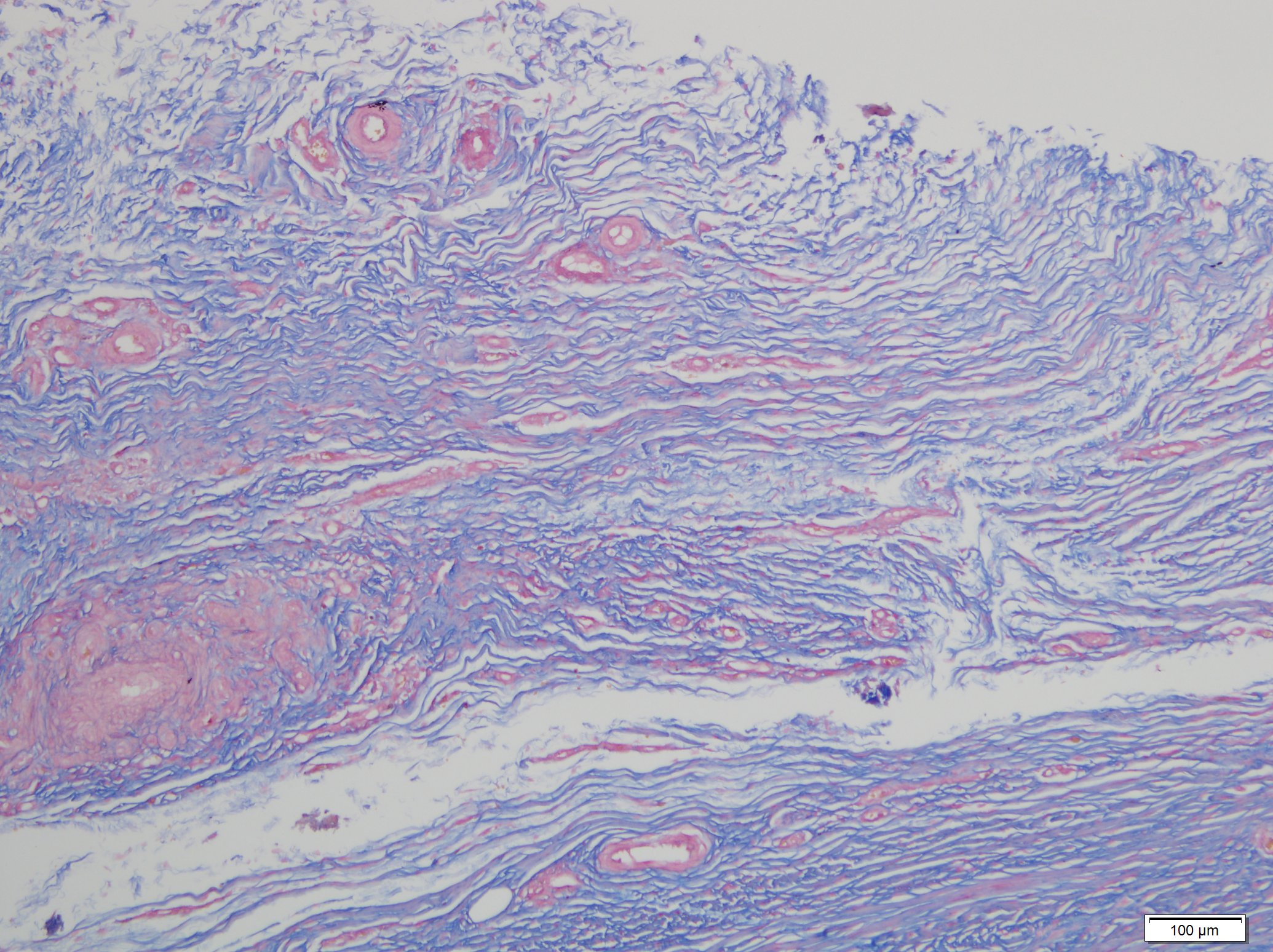

Supplement: S3 File — (ZIP) [file pone.0215499.s003.zip › masson's trichrome/1 week/2-2 10x.jpg]

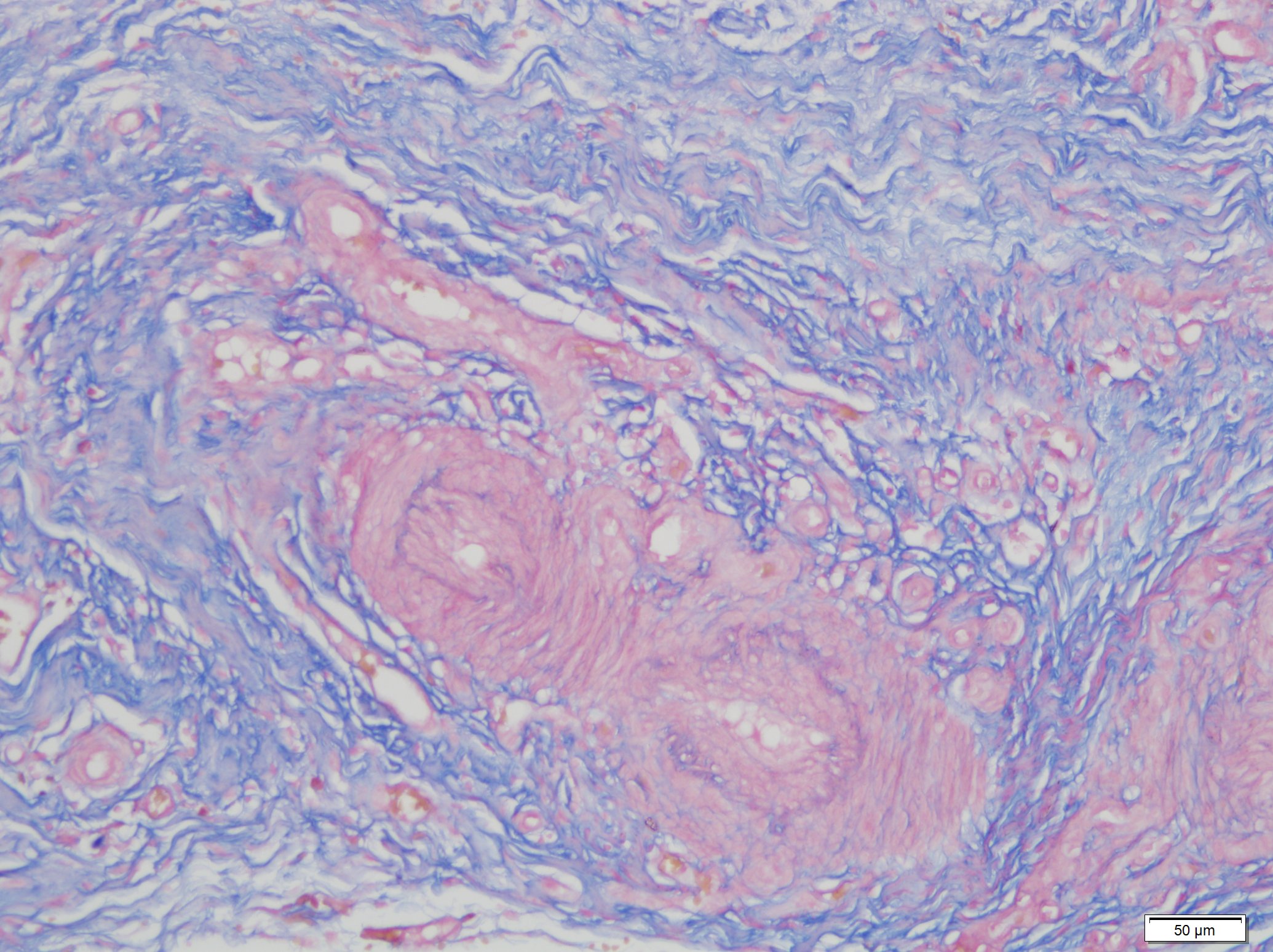

Supplement: S3 File — (ZIP) [file pone.0215499.s003.zip › masson's trichrome/1 week/2-2 20x-2.jpg]

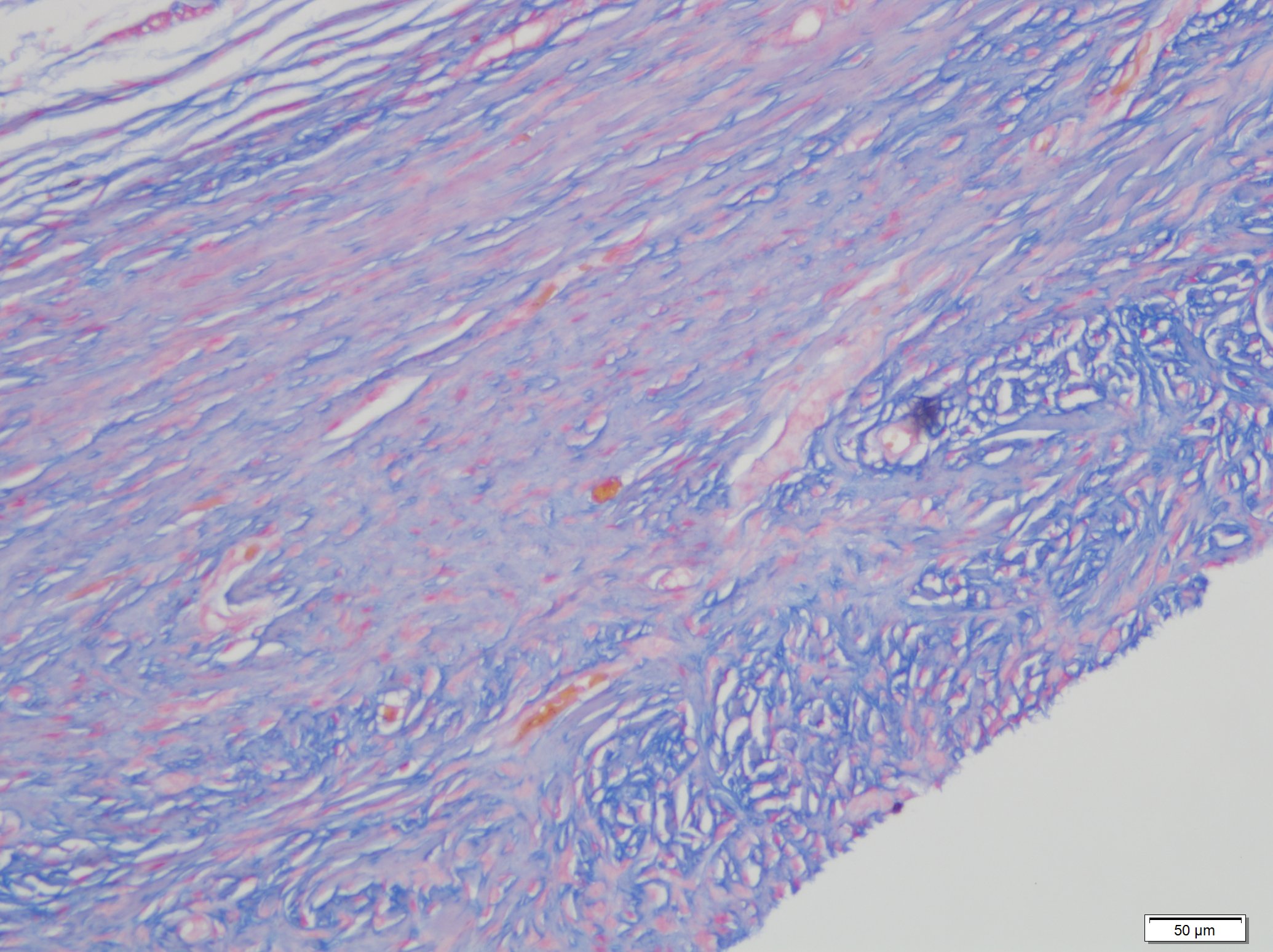

Supplement: S3 File — (ZIP) [file pone.0215499.s003.zip › masson's trichrome/1 week/2-2 20x.jpg]

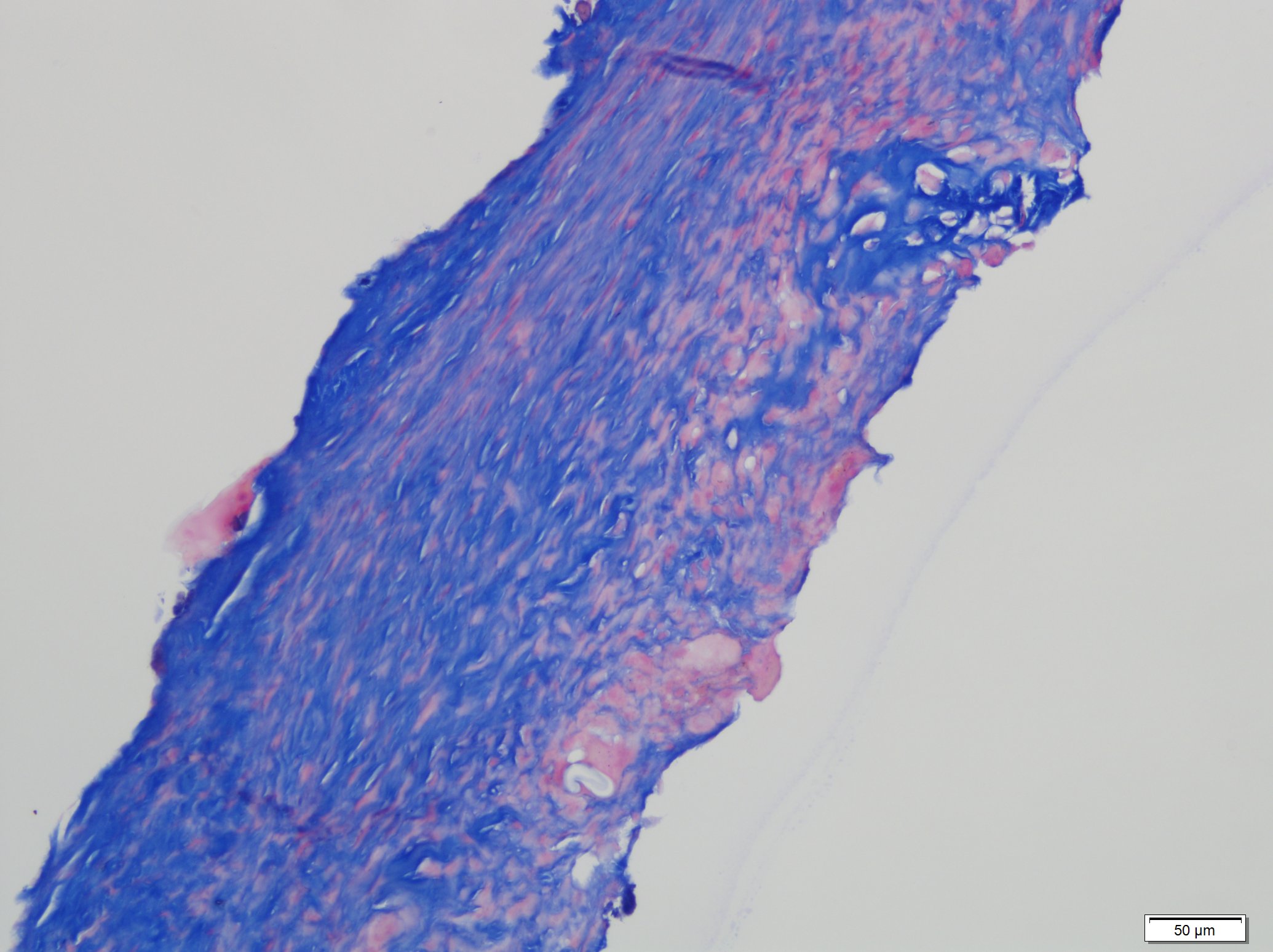

Supplement: S3 File — (ZIP) [file pone.0215499.s003.zip › masson's trichrome/2 weeks/6-4 20x-1.jpg]

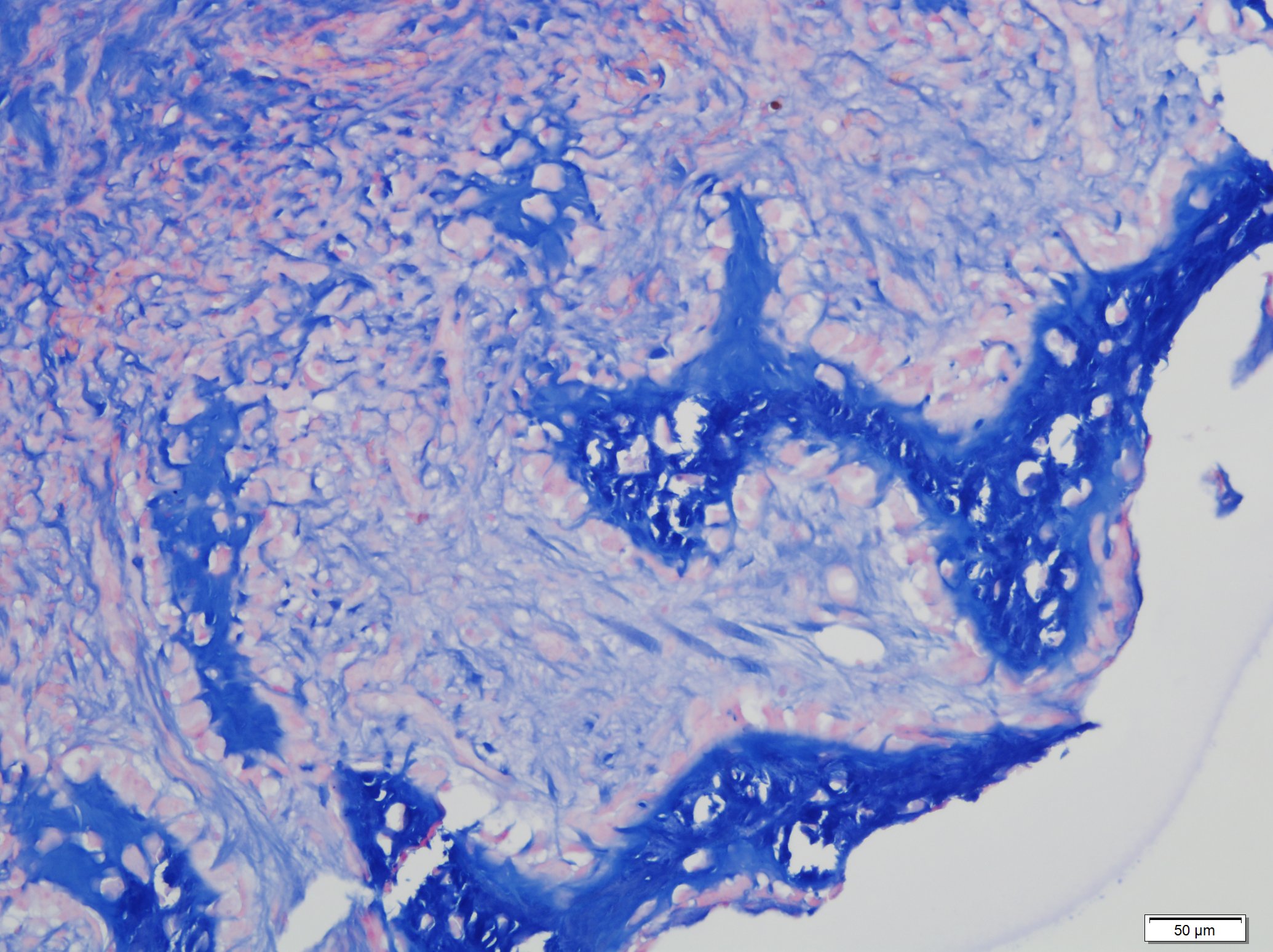

Supplement: S3 File — (ZIP) [file pone.0215499.s003.zip › masson's trichrome/2 weeks/6-4 20x-2.jpg]

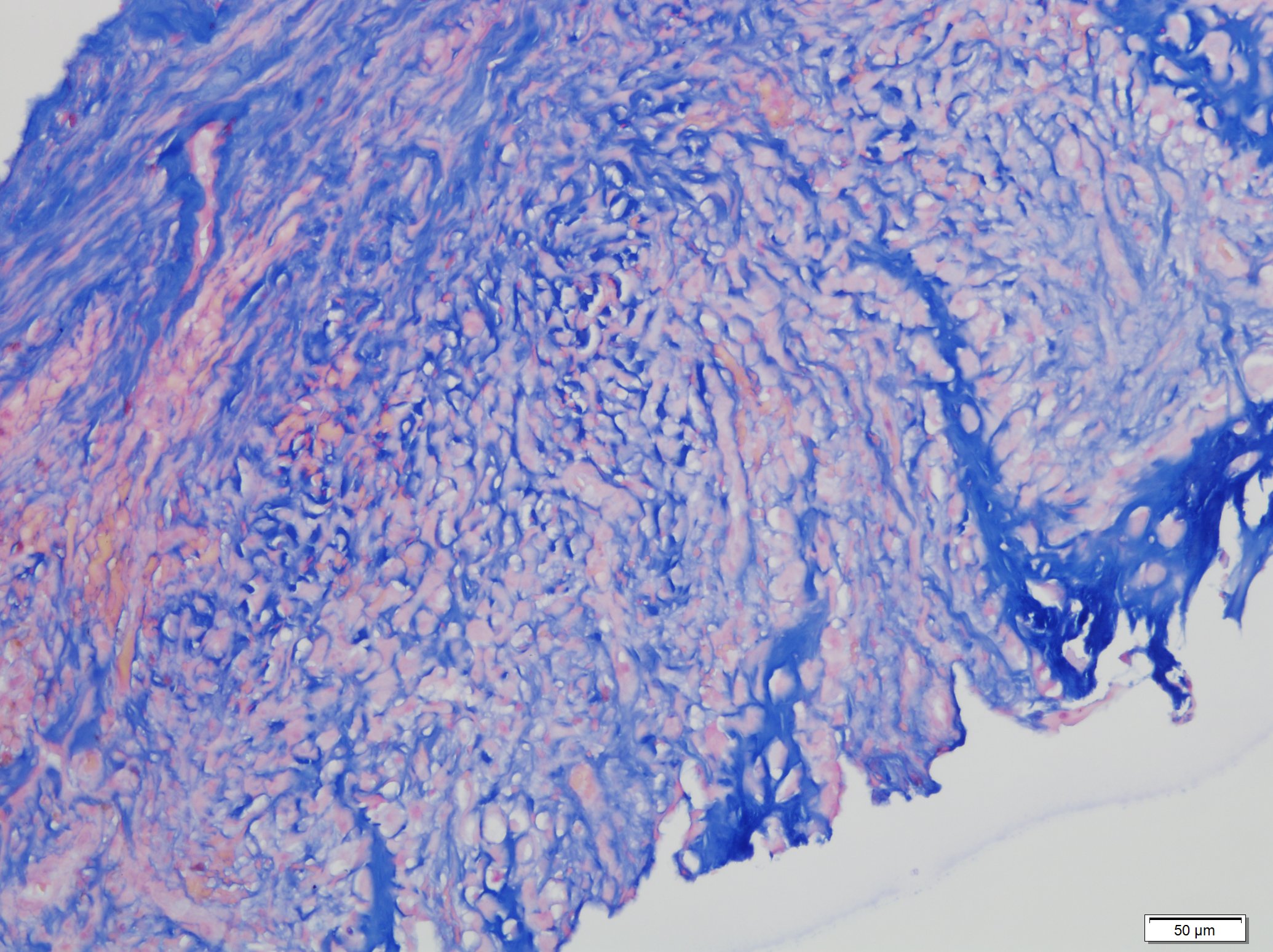

Supplement: S3 File — (ZIP) [file pone.0215499.s003.zip › masson's trichrome/2 weeks/6-4 20x-3.jpg]

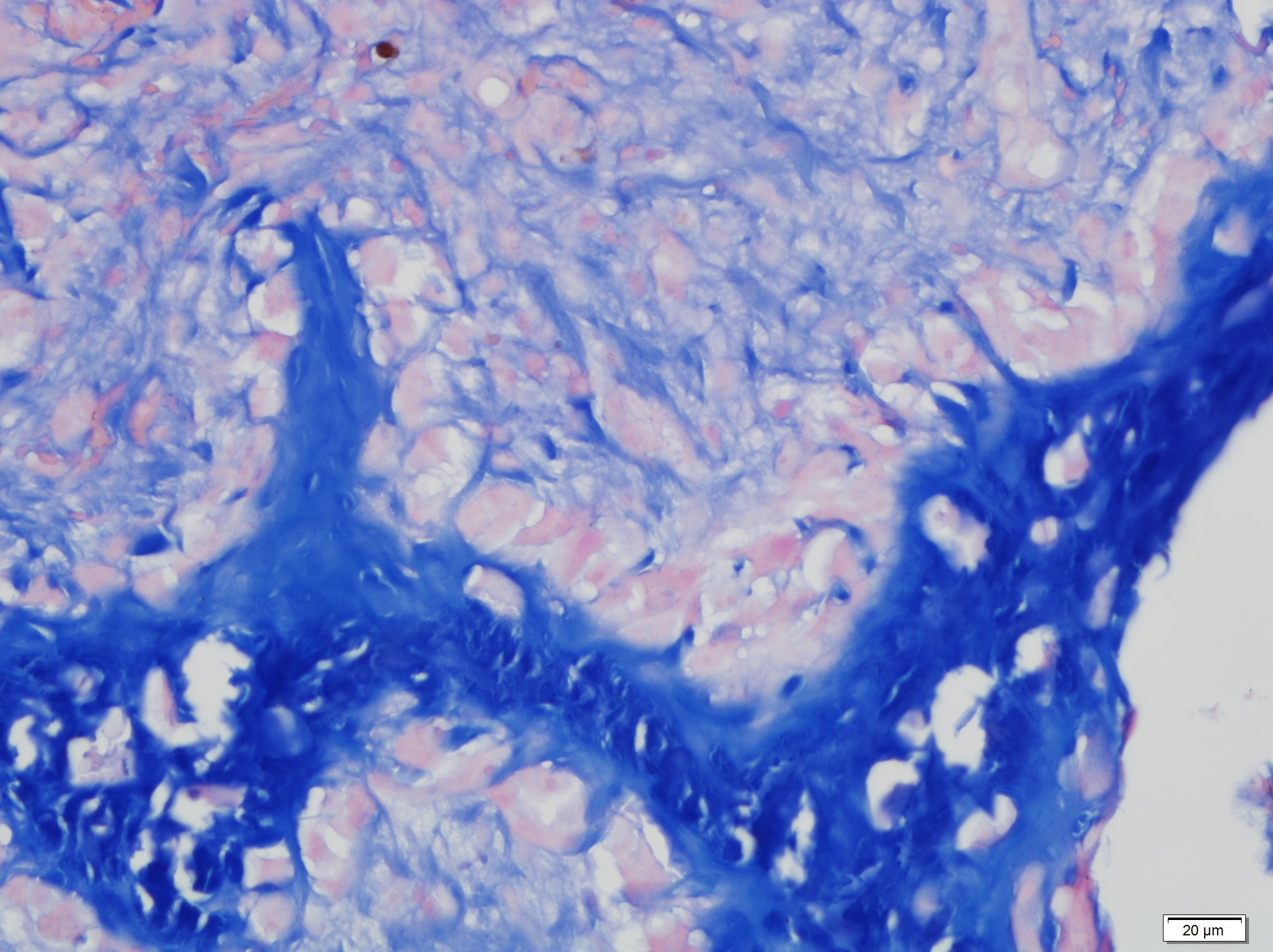

Supplement: S3 File — (ZIP) [file pone.0215499.s003.zip › masson's trichrome/2 weeks/6-4 40x-1.jpg]

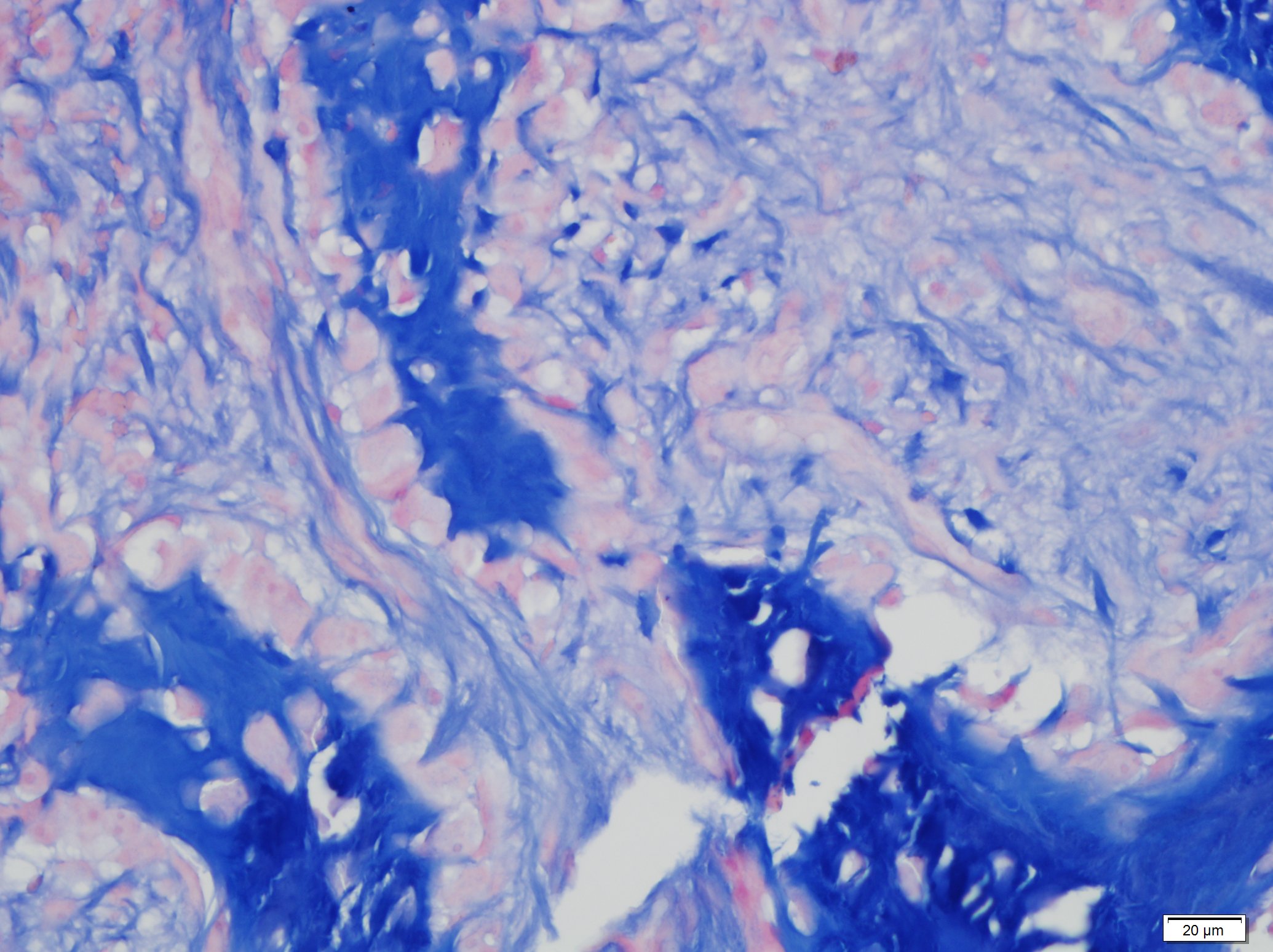

Supplement: S3 File — (ZIP) [file pone.0215499.s003.zip › masson's trichrome/2 weeks/6-4 40x-2.jpg]

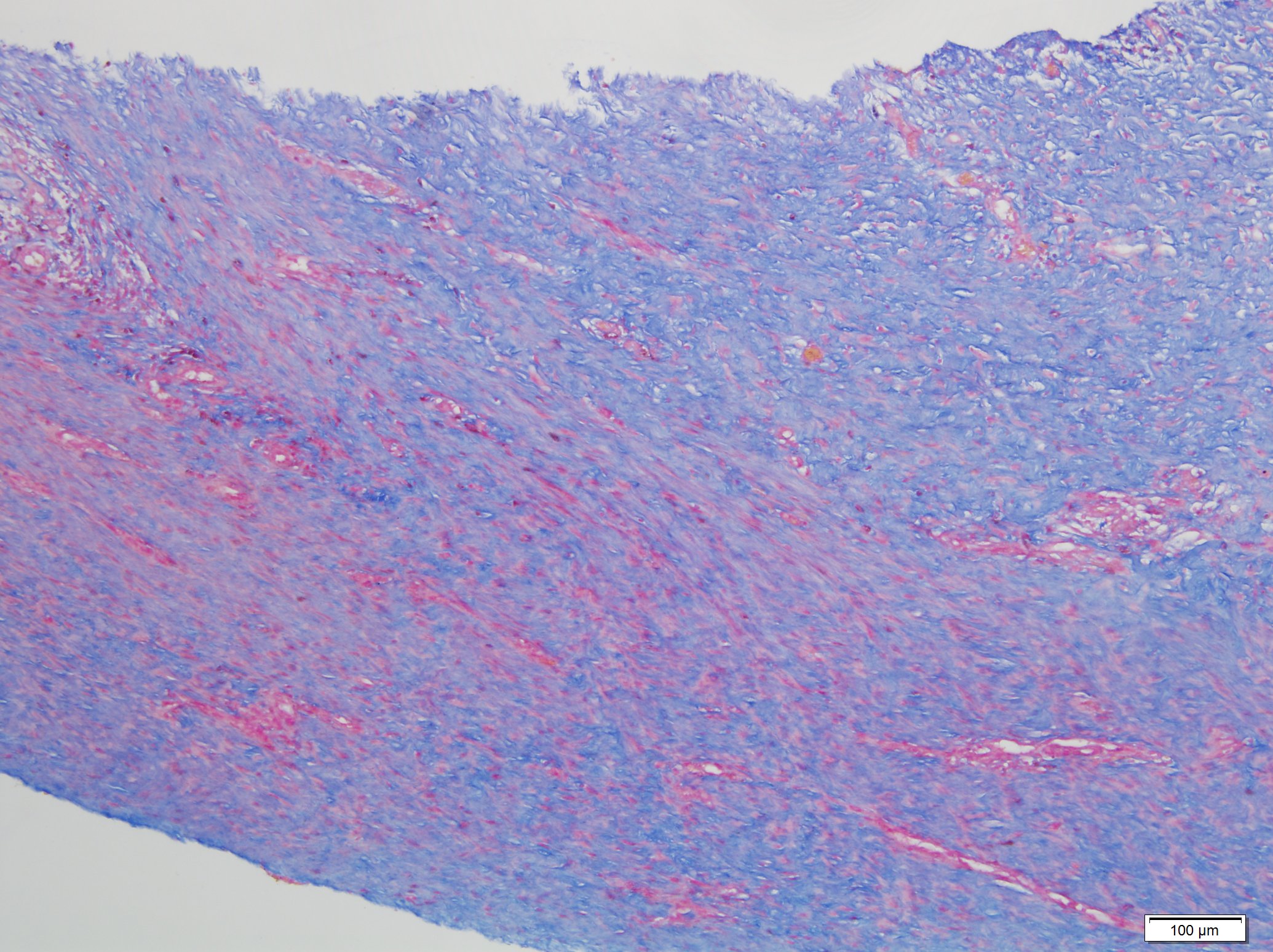

Supplement: S3 File — (ZIP) [file pone.0215499.s003.zip › masson's trichrome/2 weeks/6-6 10x-2.jpg]

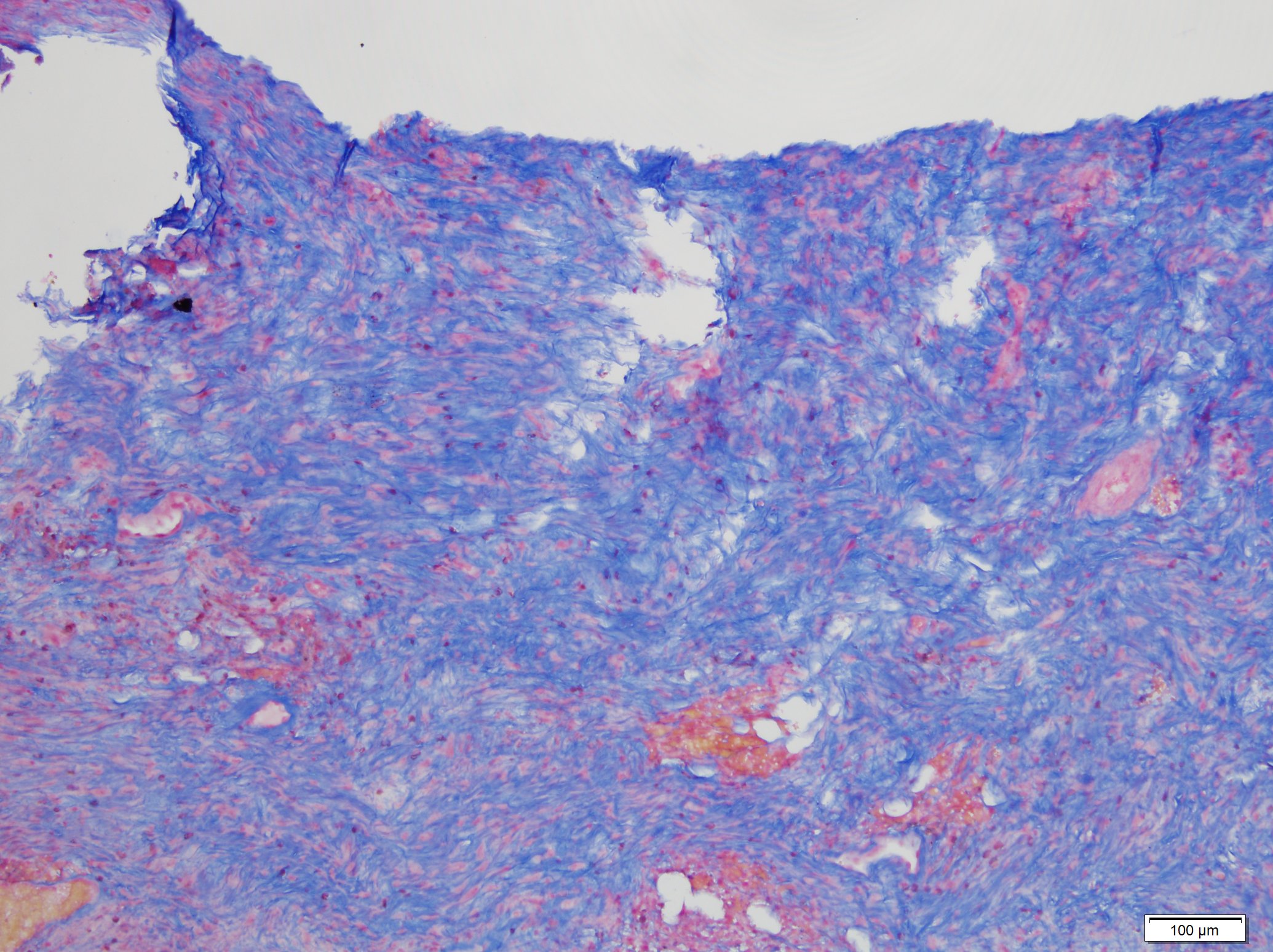

Supplement: S3 File — (ZIP) [file pone.0215499.s003.zip › masson's trichrome/2 weeks/6-7 10x-1.jpg]

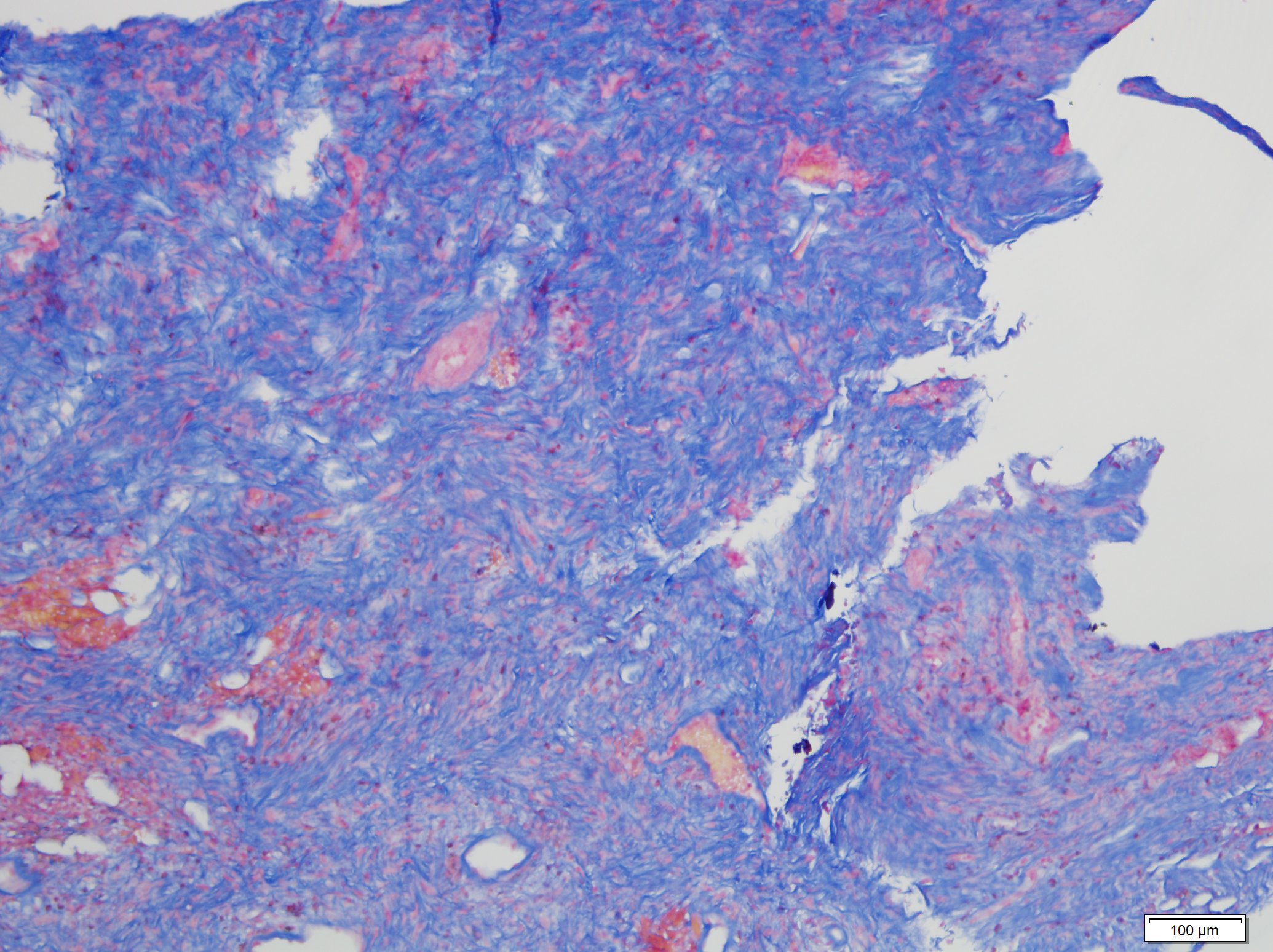

Supplement: S3 File — (ZIP) [file pone.0215499.s003.zip › masson's trichrome/2 weeks/6-7 10x-2.jpg]

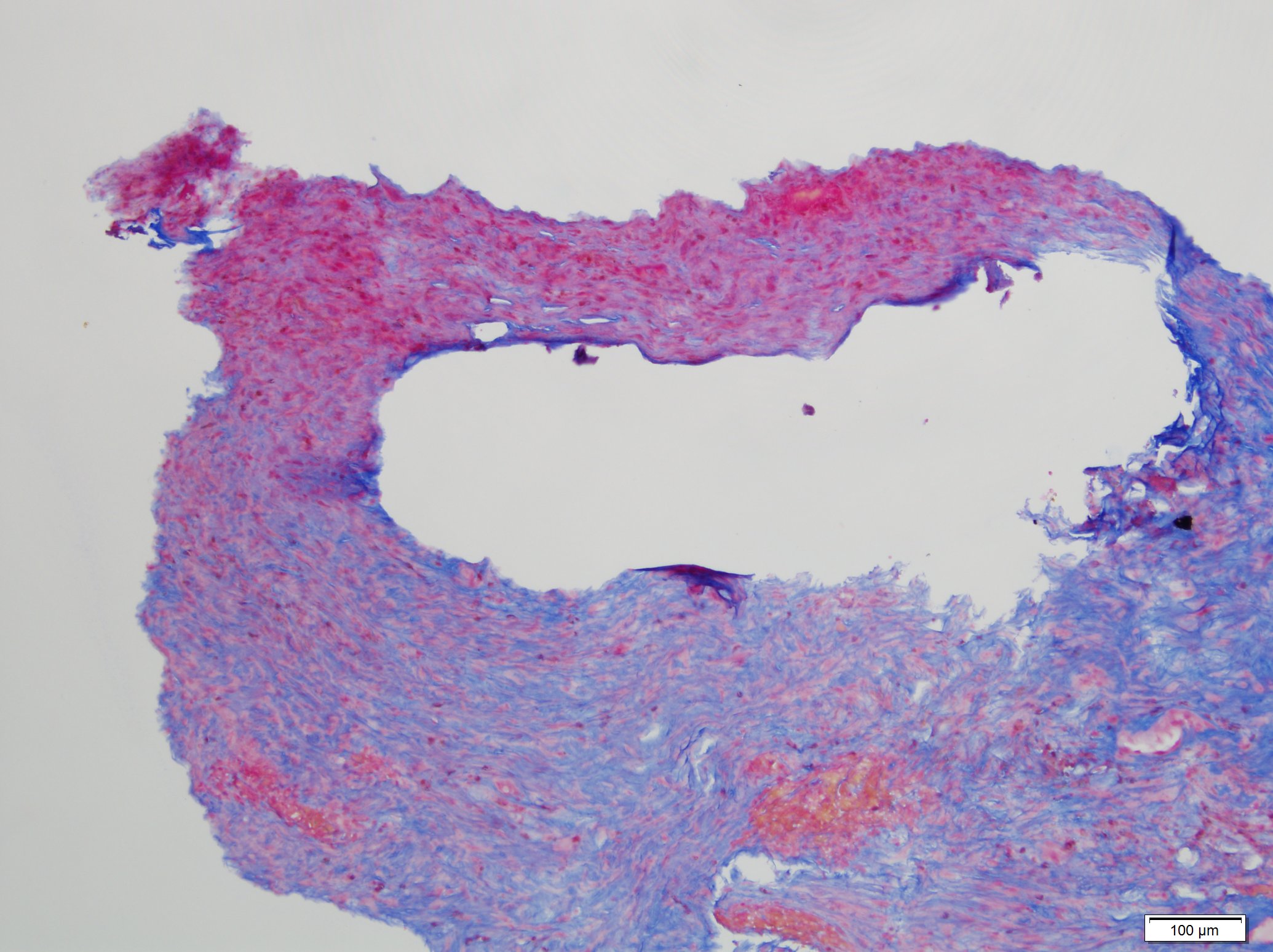

Supplement: S3 File — (ZIP) [file pone.0215499.s003.zip › masson's trichrome/2 weeks/6-7 10x-3.jpg]

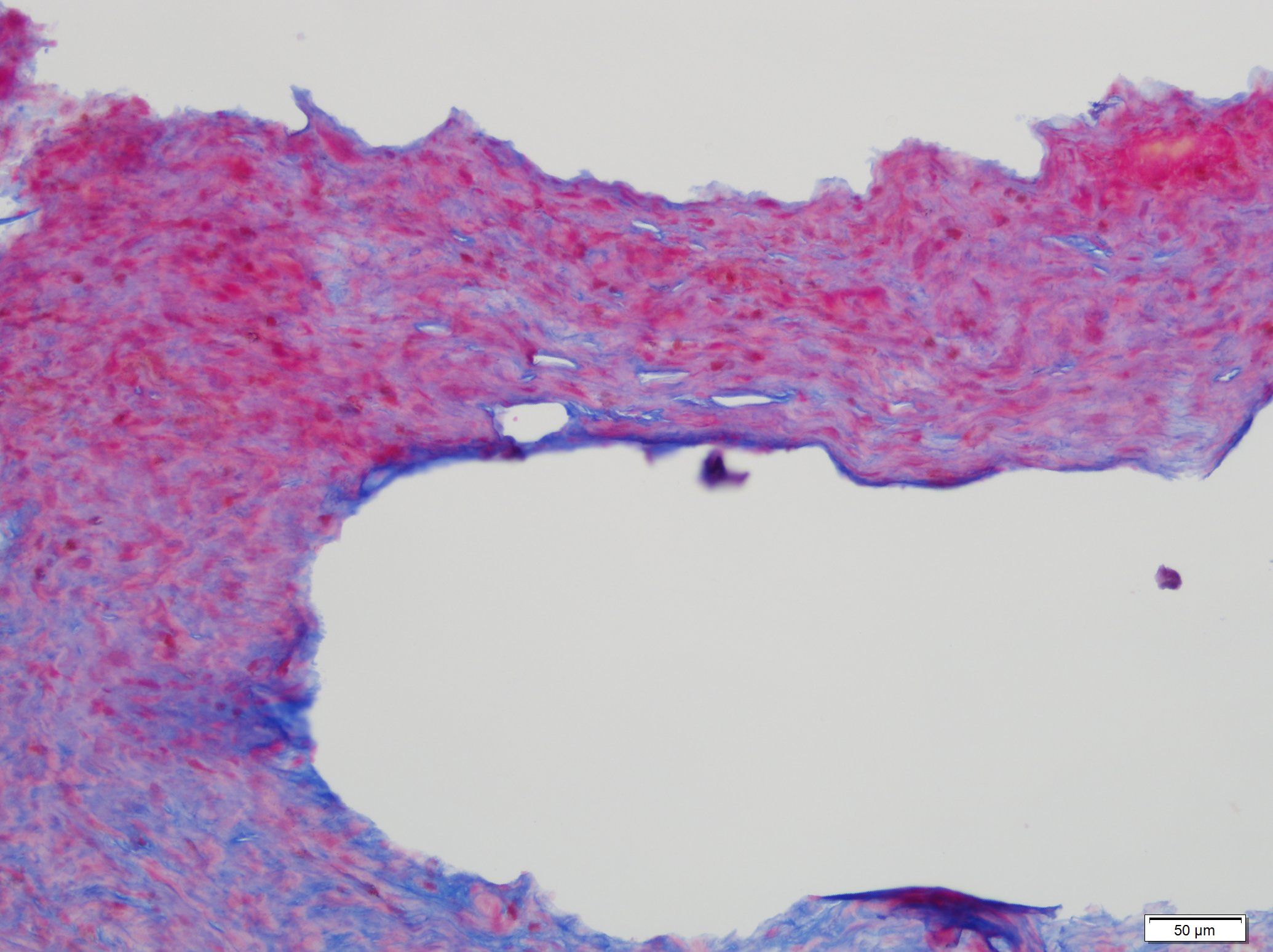

Supplement: S3 File — (ZIP) [file pone.0215499.s003.zip › masson's trichrome/2 weeks/6-7 20x-1.jpg]

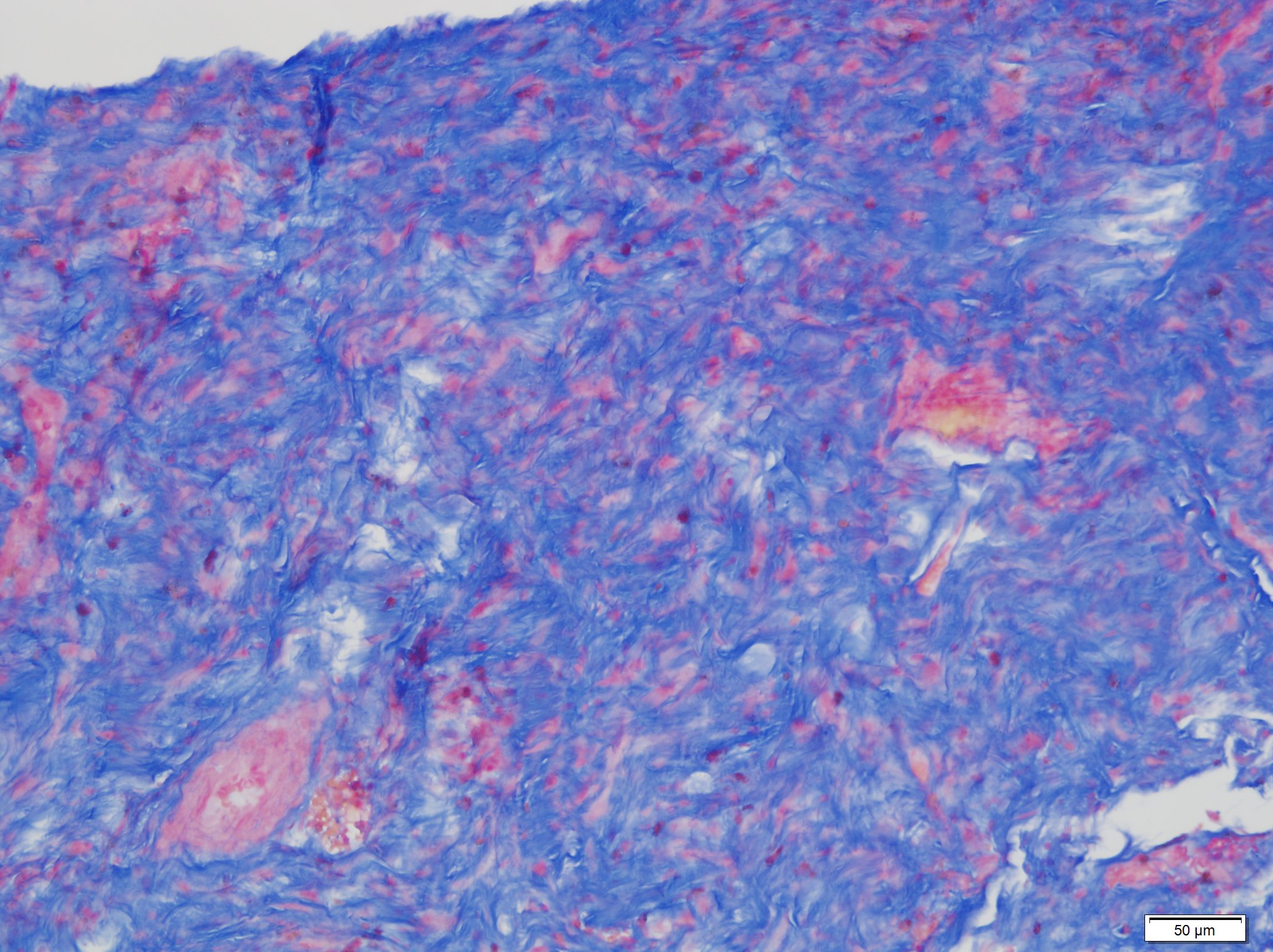

Supplement: S3 File — (ZIP) [file pone.0215499.s003.zip › masson's trichrome/2 weeks/6-7 20x-2.jpg]

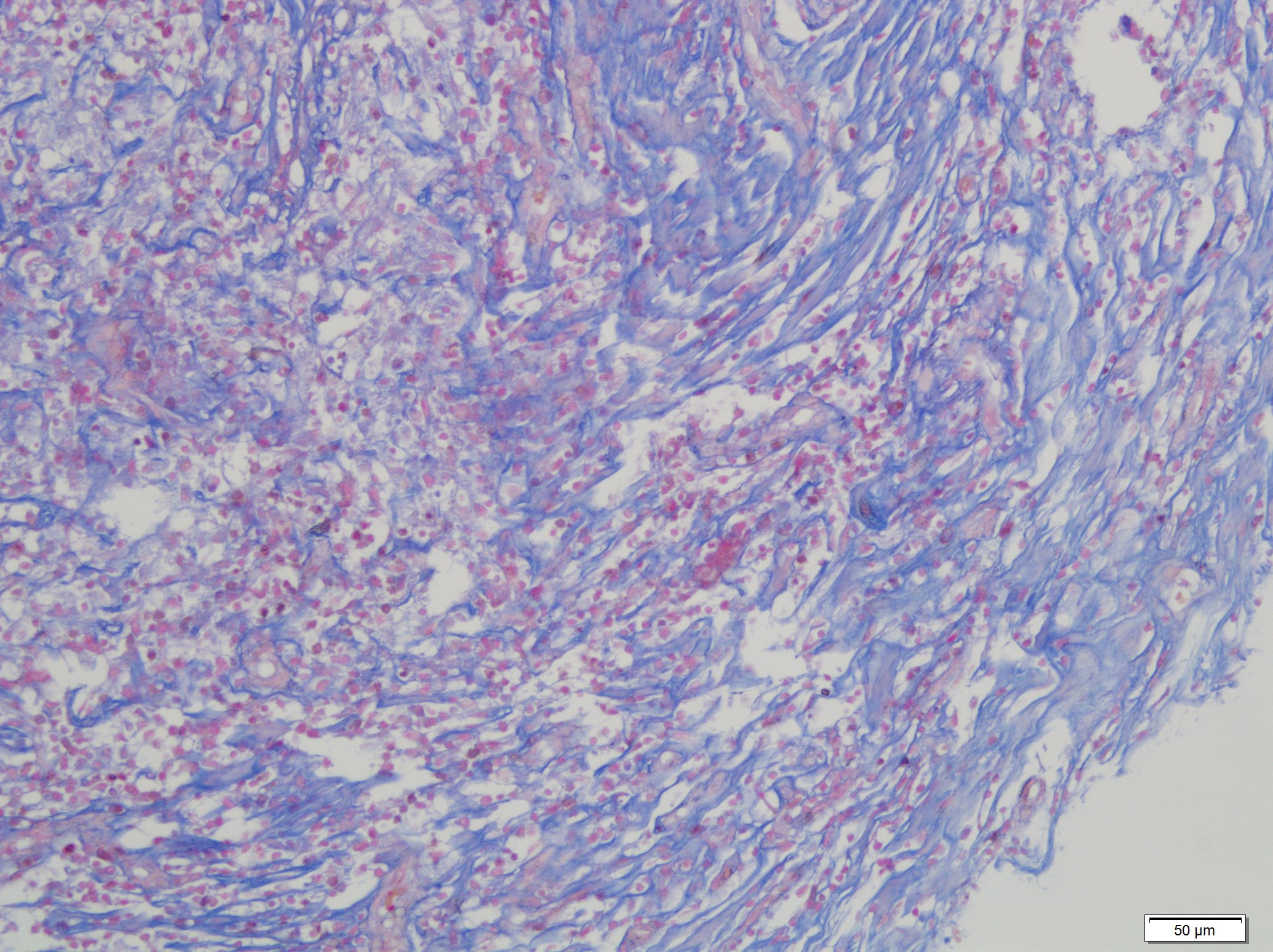

Supplement: S3 File — (ZIP) [file pone.0215499.s003.zip › masson's trichrome/3 weeks/5-5 20x-3.jpg]

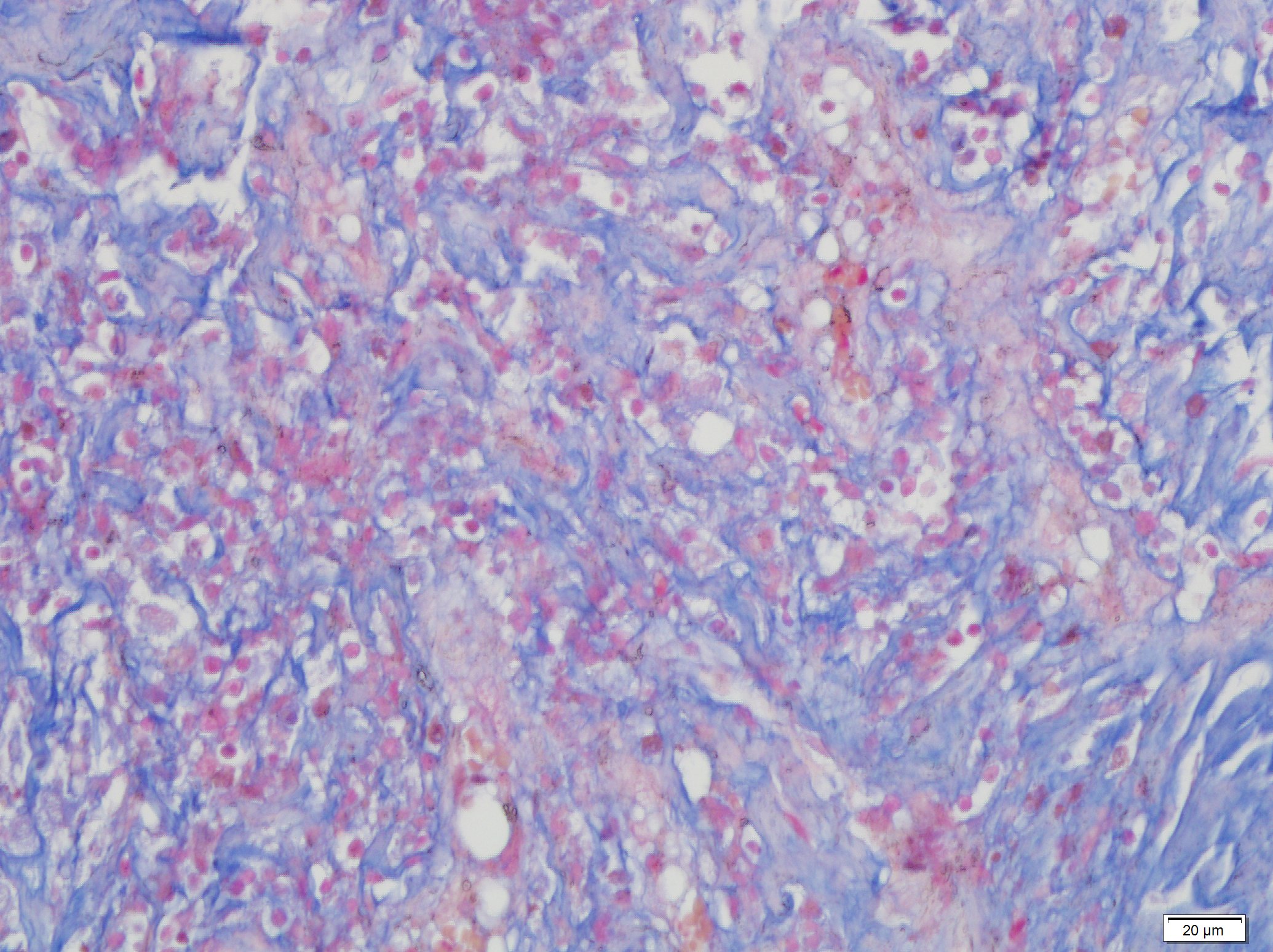

Supplement: S3 File — (ZIP) [file pone.0215499.s003.zip › masson's trichrome/3 weeks/5-5 40x-1.jpg]

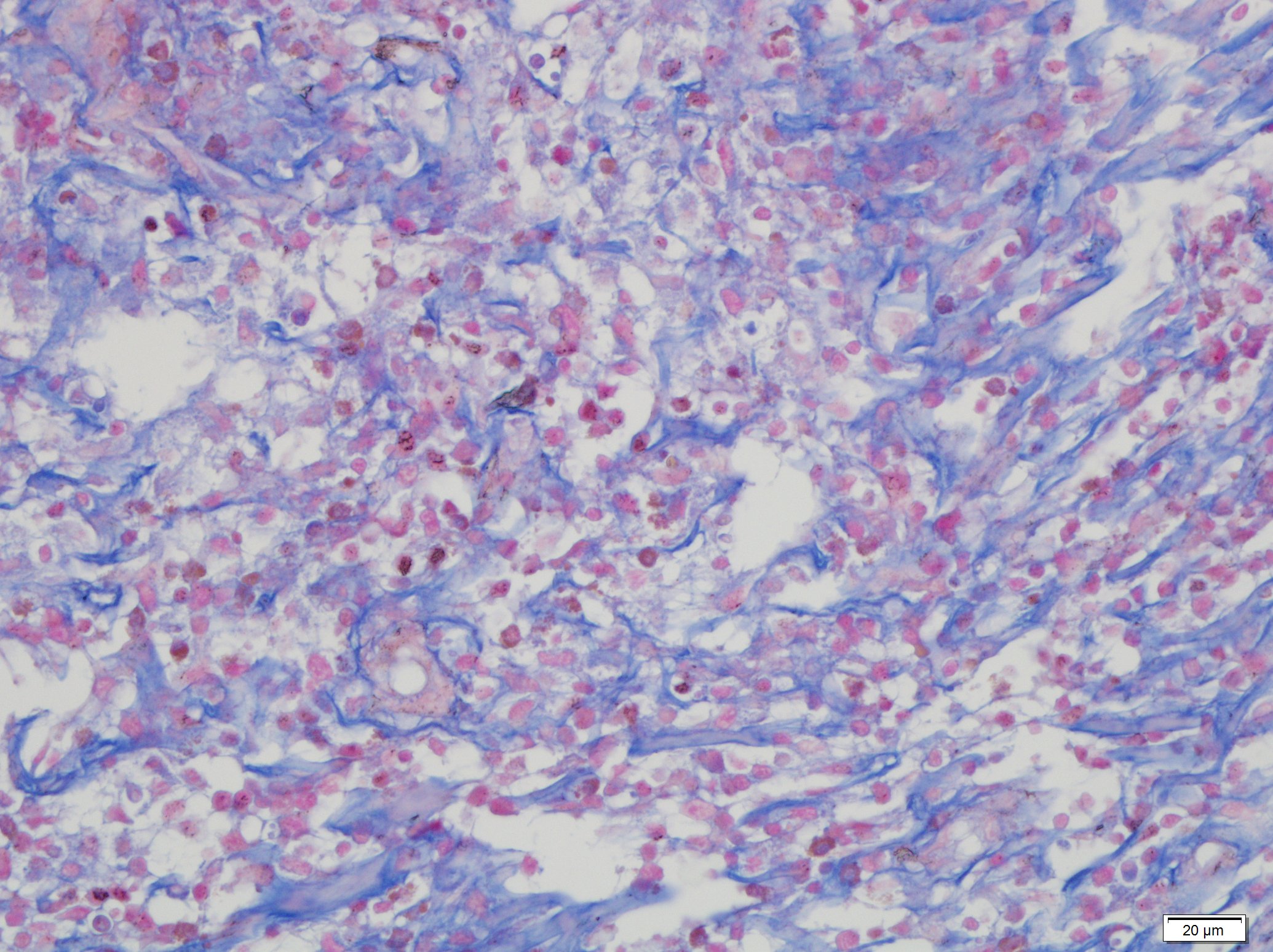

Supplement: S3 File — (ZIP) [file pone.0215499.s003.zip › masson's trichrome/3 weeks/5-5 40x-2.jpg]

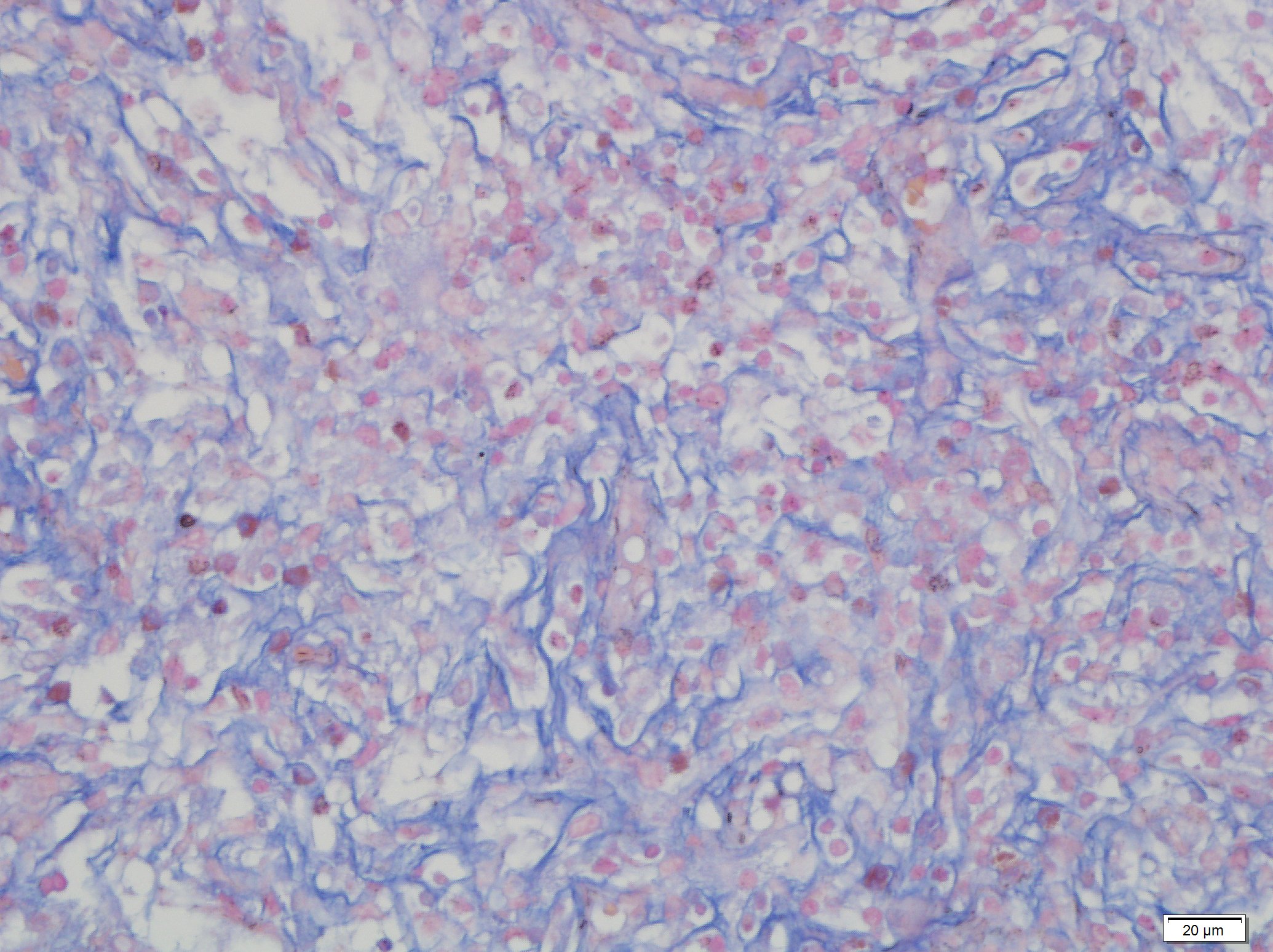

Supplement: S3 File — (ZIP) [file pone.0215499.s003.zip › masson's trichrome/3 weeks/5-5 40x-3.jpg]
