# Supplementary figures and images for: Bone regeneration in Ds-Red pig calvarial defect using allogenic transplantation of EGFP-pMSCs – A comparison of host cells and seeding cells in the scaffold (part 2 of 2)
Source: PLoS One. 2019 Jul 18;14(7):e0215499. doi: 10.1371/journal.pone.0215499 (PMC6638893; doi:10.1371/journal.pone.0215499)

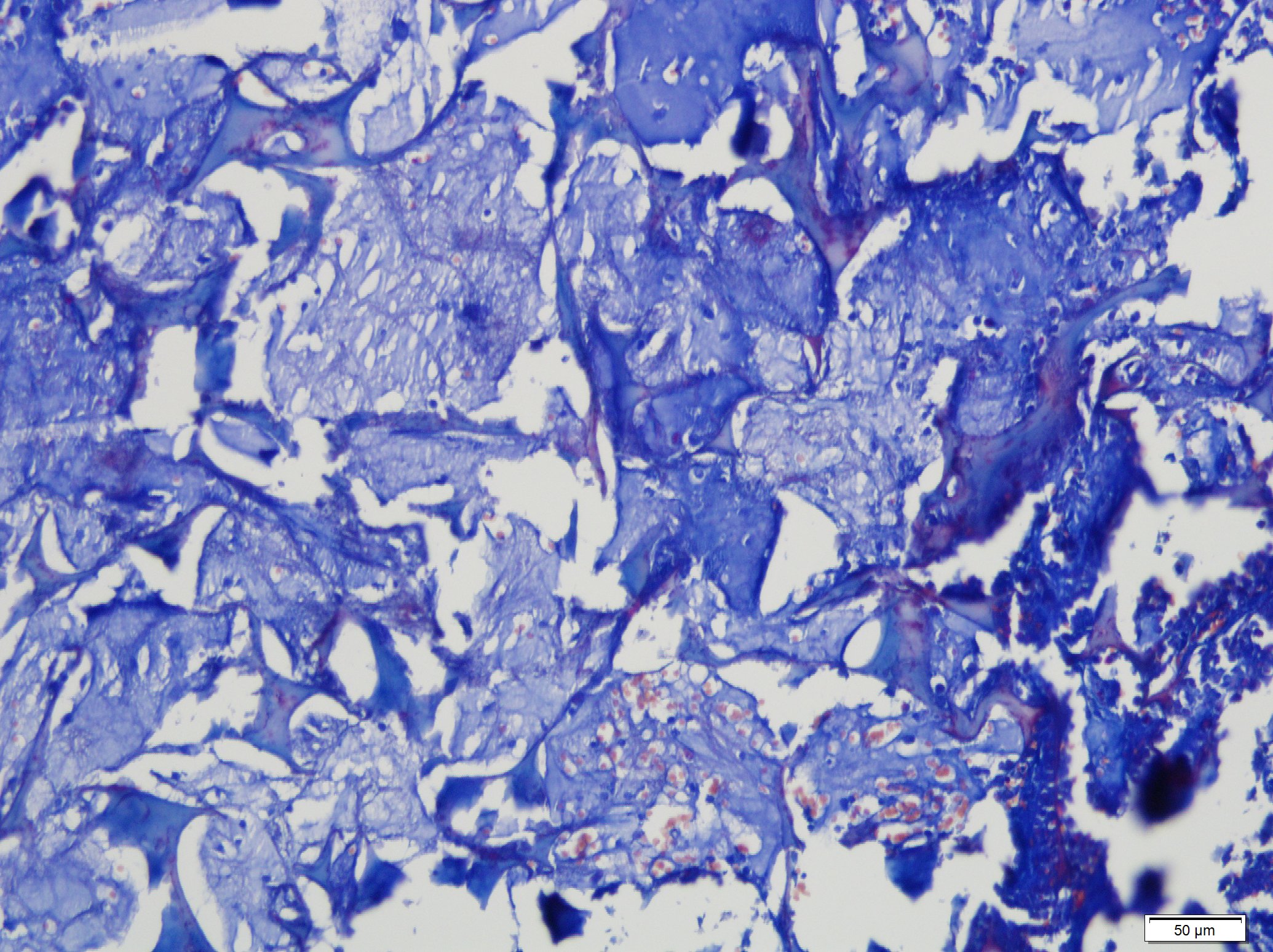

Supplement: S3 File — (ZIP) [file pone.0215499.s003.zip › masson's trichrome/3 weeks/5-6 20x-2.jpg]

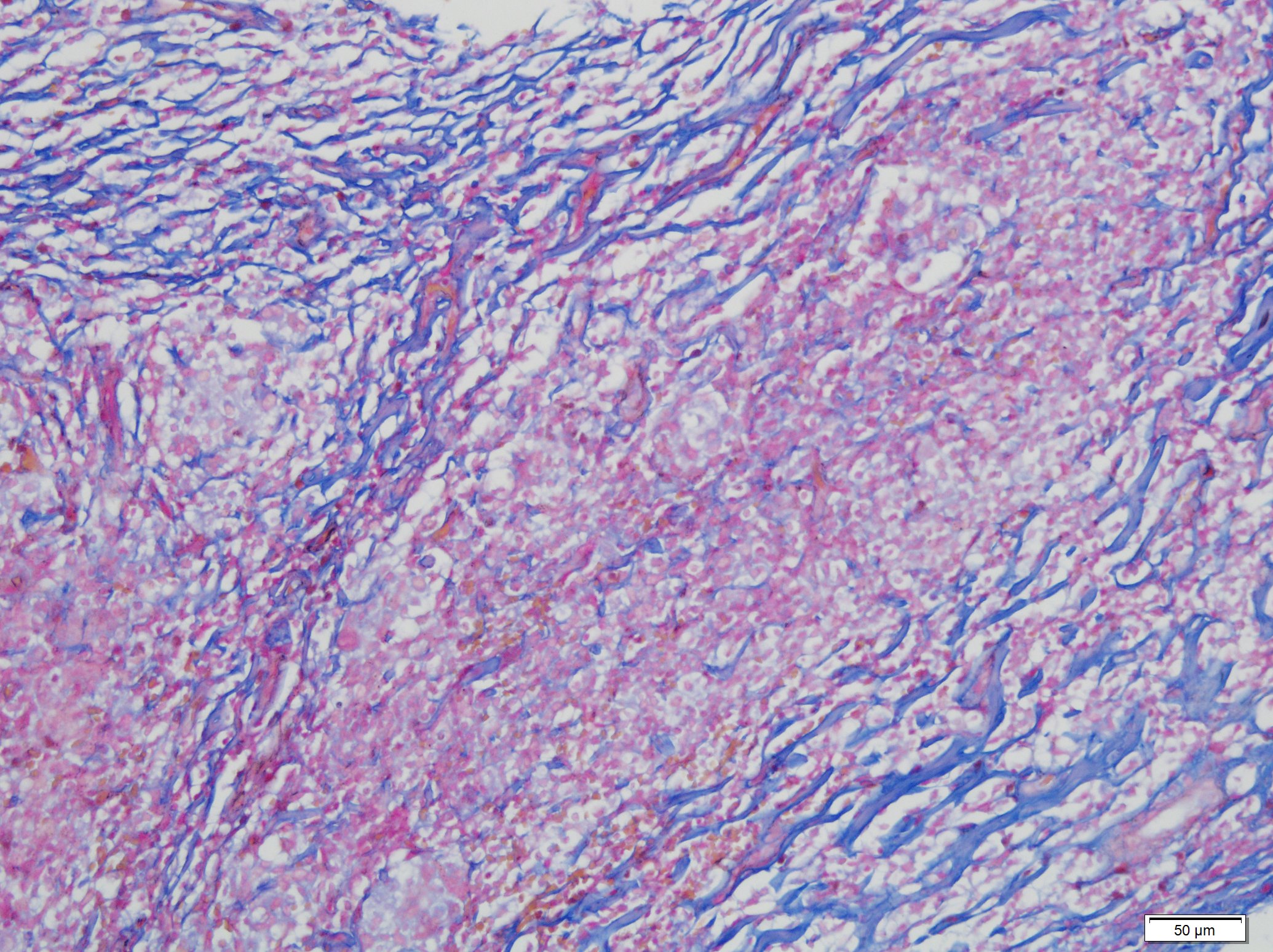

Supplement: S3 File — (ZIP) [file pone.0215499.s003.zip › masson's trichrome/3 weeks/5-7 20x-2.jpg]

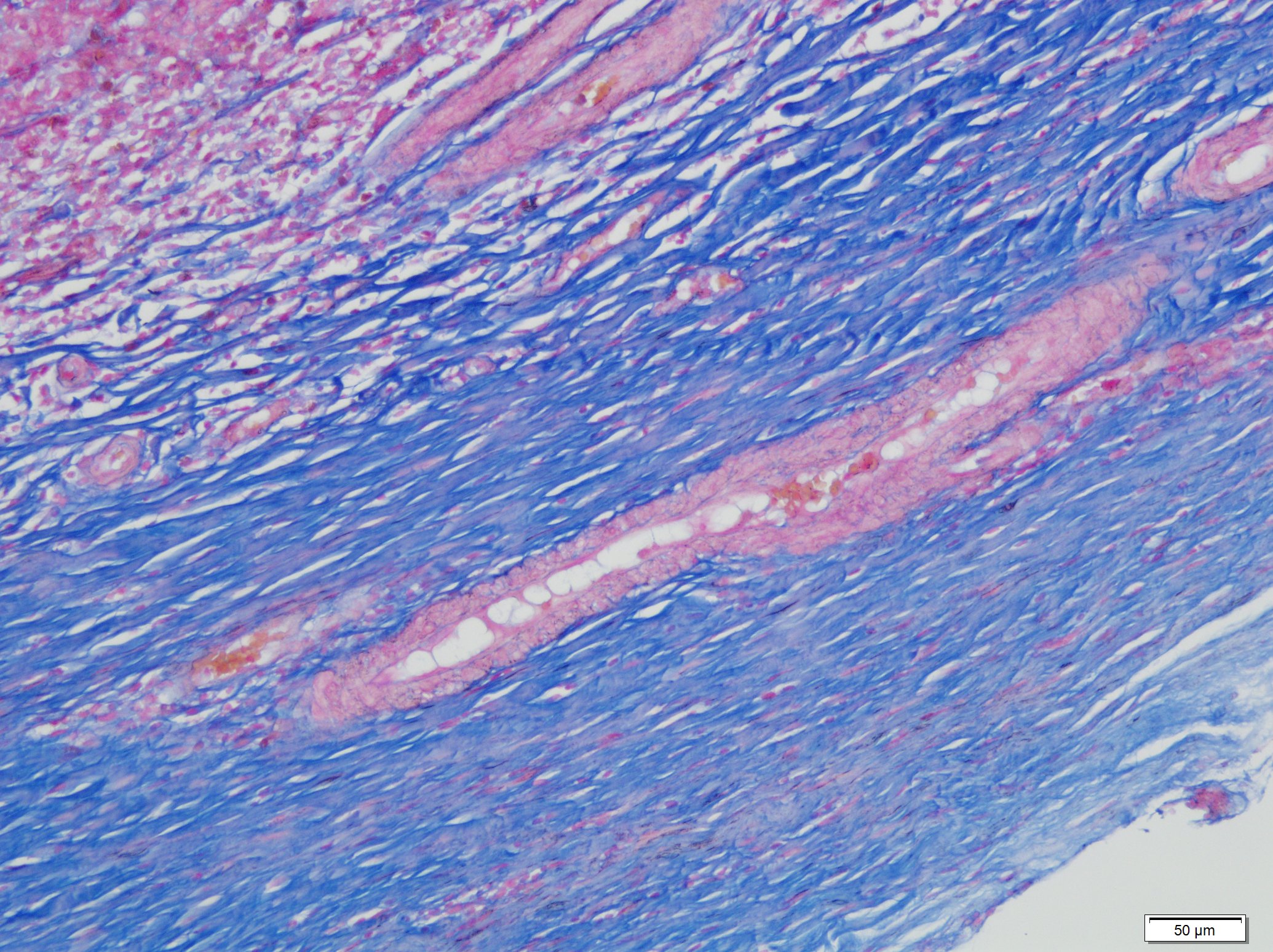

Supplement: S3 File — (ZIP) [file pone.0215499.s003.zip › masson's trichrome/3 weeks/5-7 20x-3.jpg]

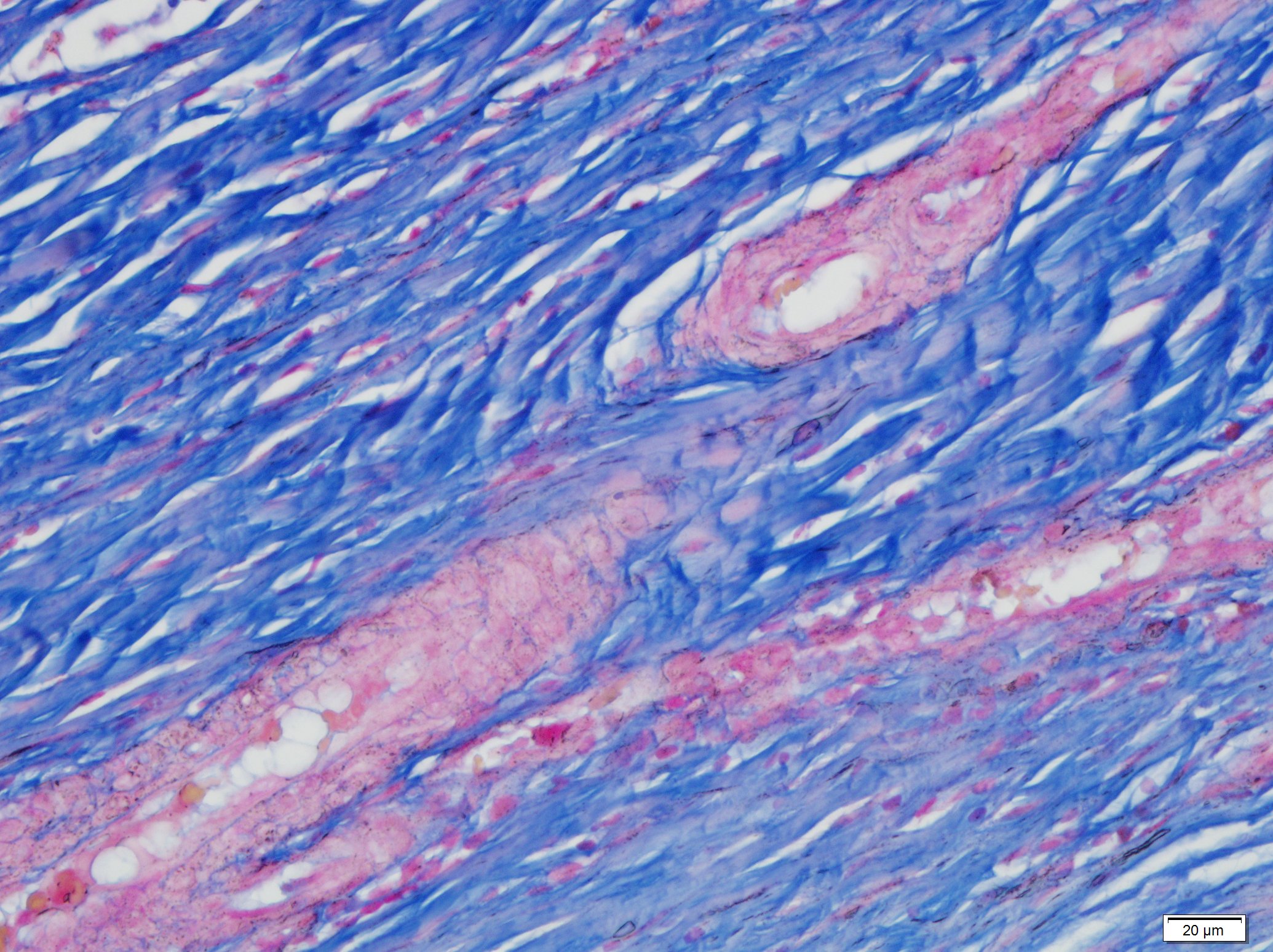

Supplement: S3 File — (ZIP) [file pone.0215499.s003.zip › masson's trichrome/3 weeks/5-7 40x-2.jpg]

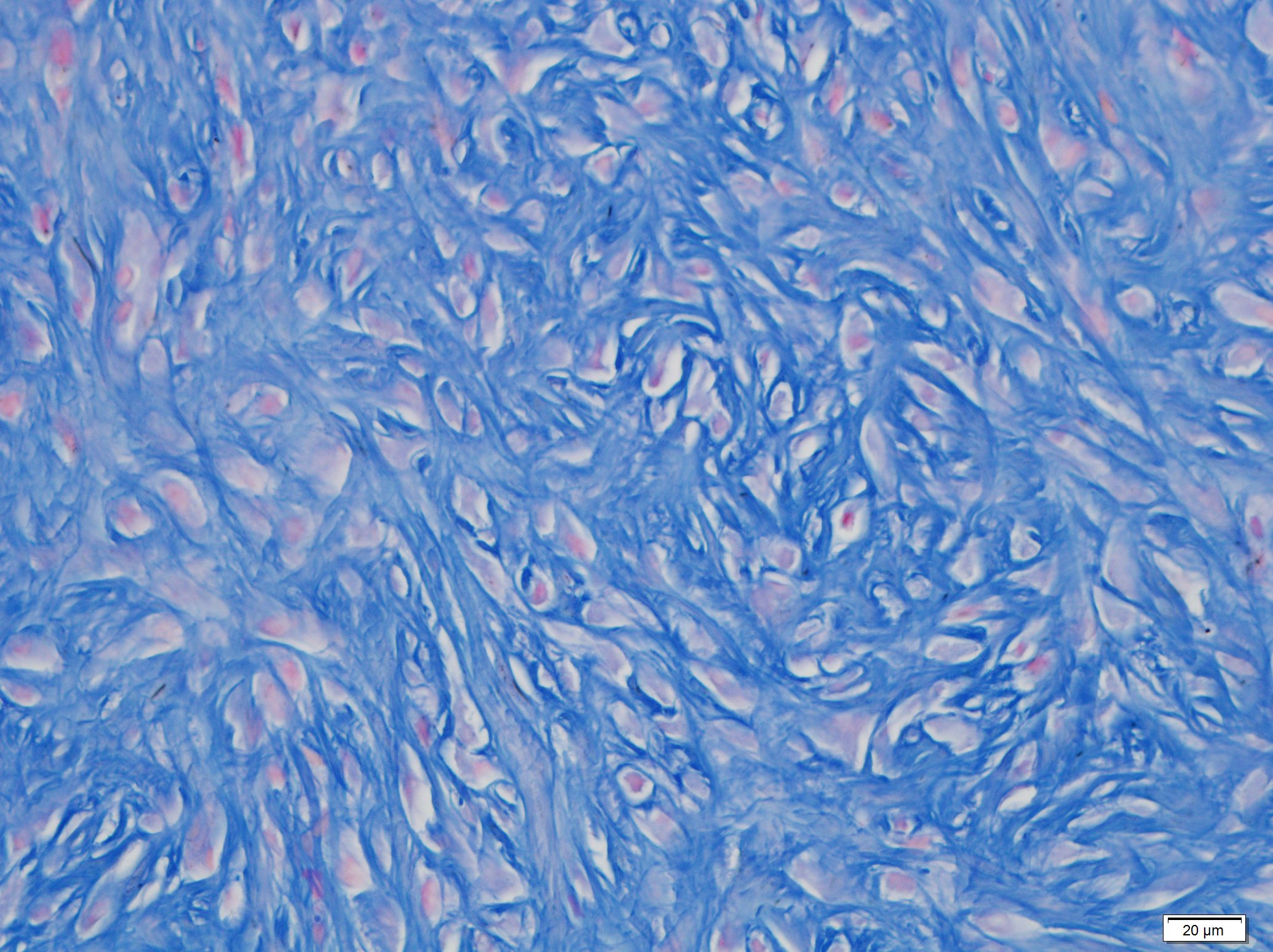

Supplement: S3 File — (ZIP) [file pone.0215499.s003.zip › masson's trichrome/4 weeks/4-4 40x.jpg]

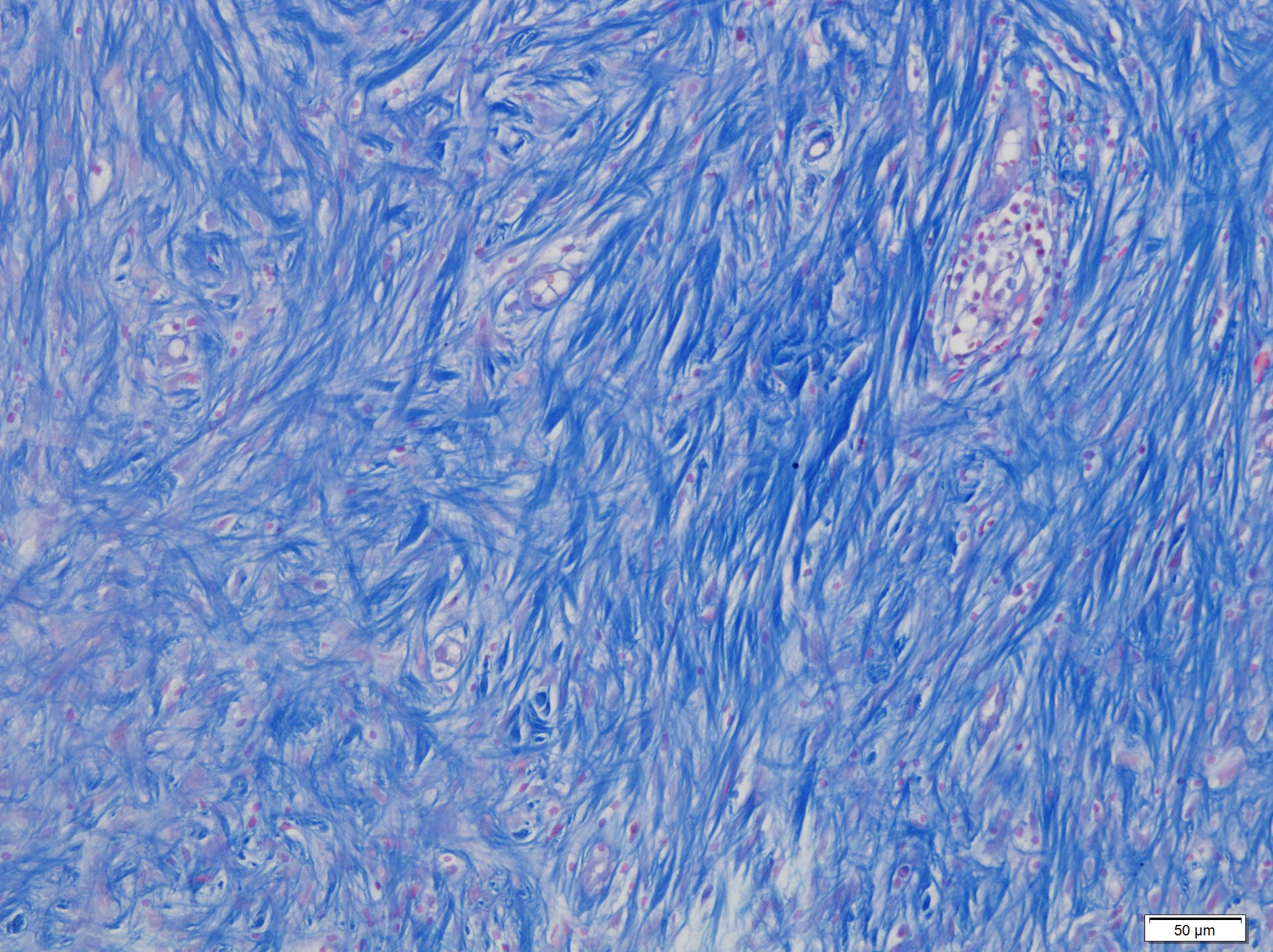

Supplement: S3 File — (ZIP) [file pone.0215499.s003.zip › masson's trichrome/4 weeks/4-6 20x-3.jpg]

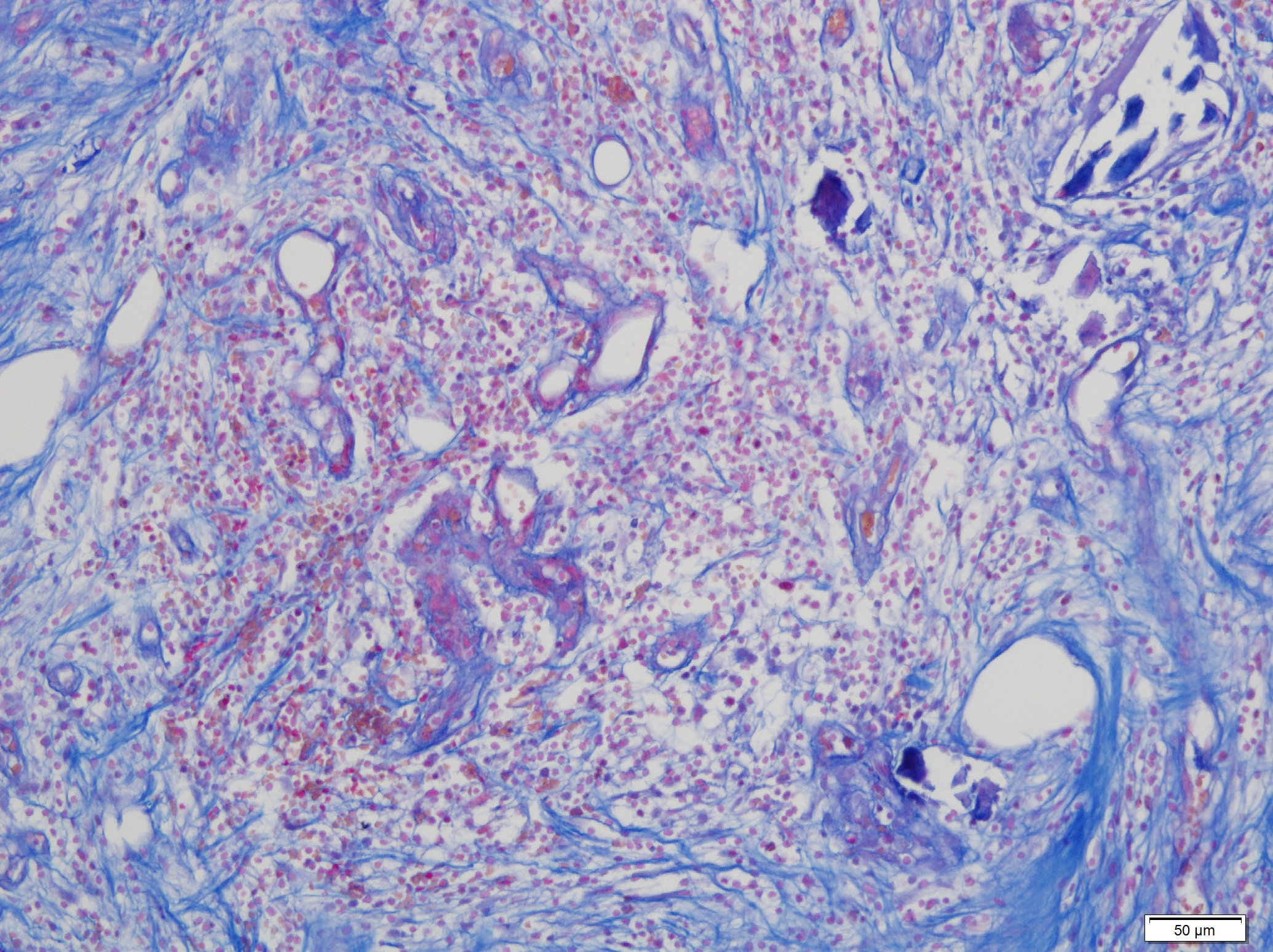

Supplement: S3 File — (ZIP) [file pone.0215499.s003.zip › masson's trichrome/4 weeks/4-6 20x.jpg]

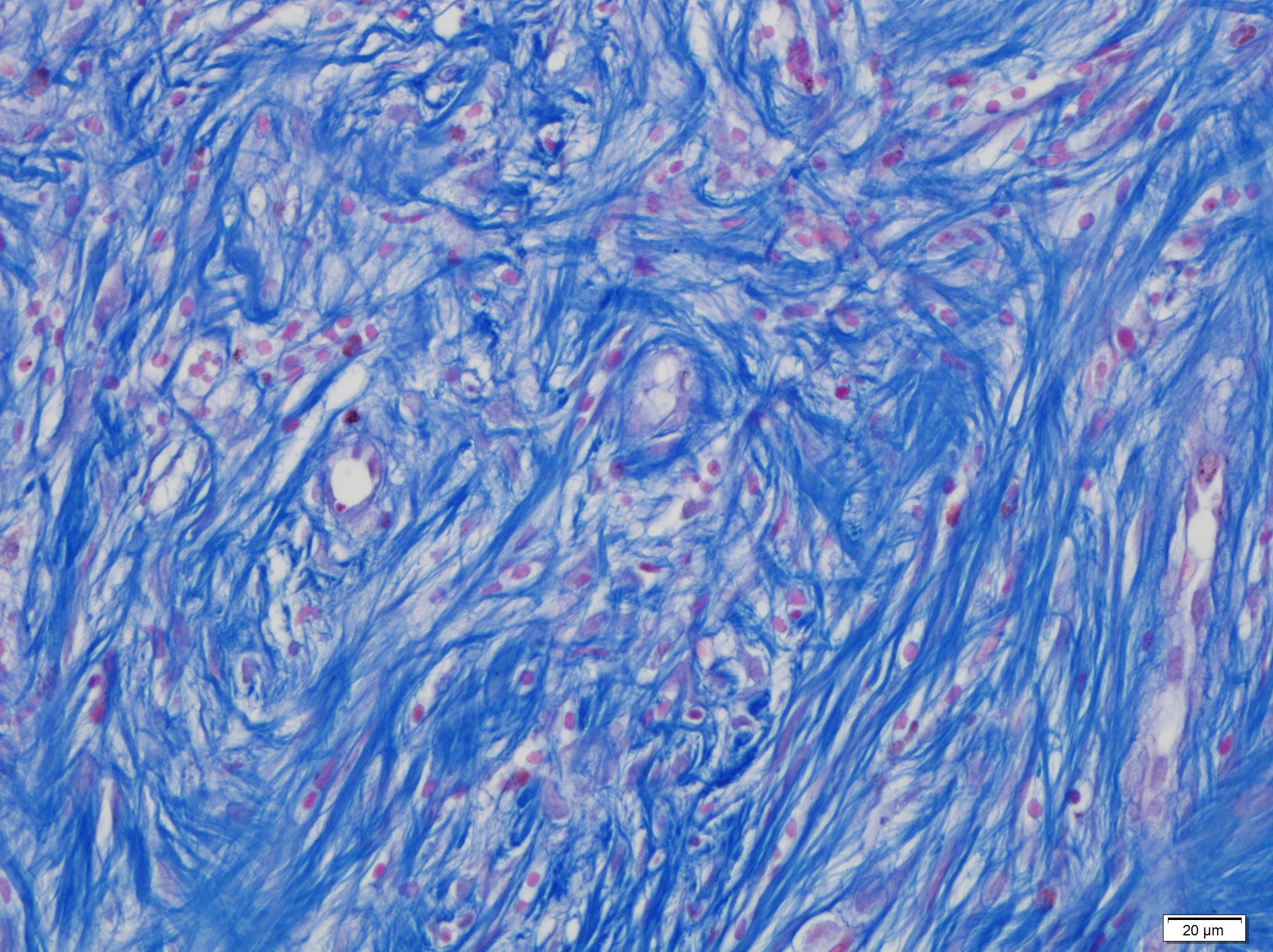

Supplement: S3 File — (ZIP) [file pone.0215499.s003.zip › masson's trichrome/4 weeks/4-6 40x-3.jpg]

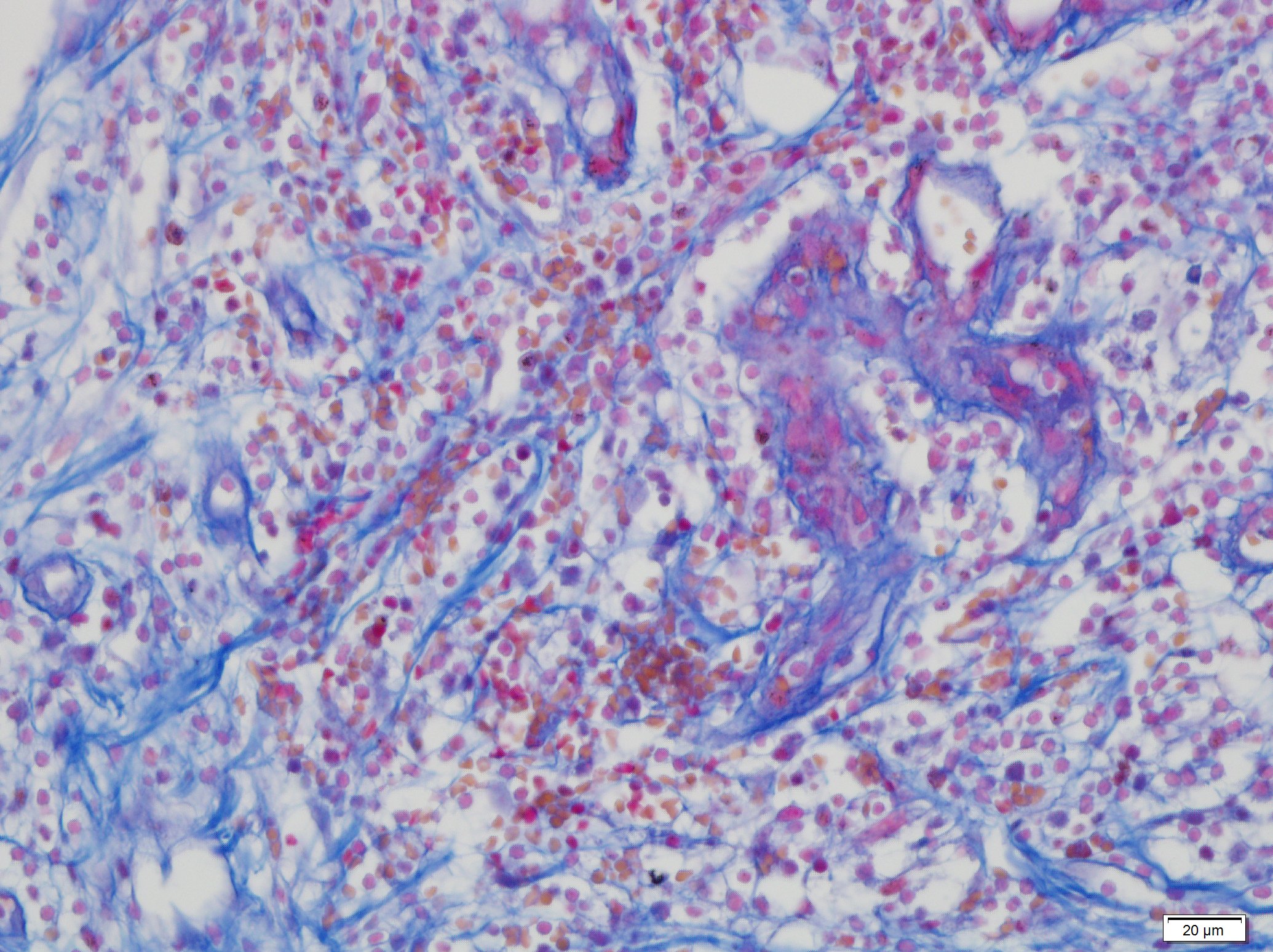

Supplement: S3 File — (ZIP) [file pone.0215499.s003.zip › masson's trichrome/4 weeks/4-6 40x.jpg]

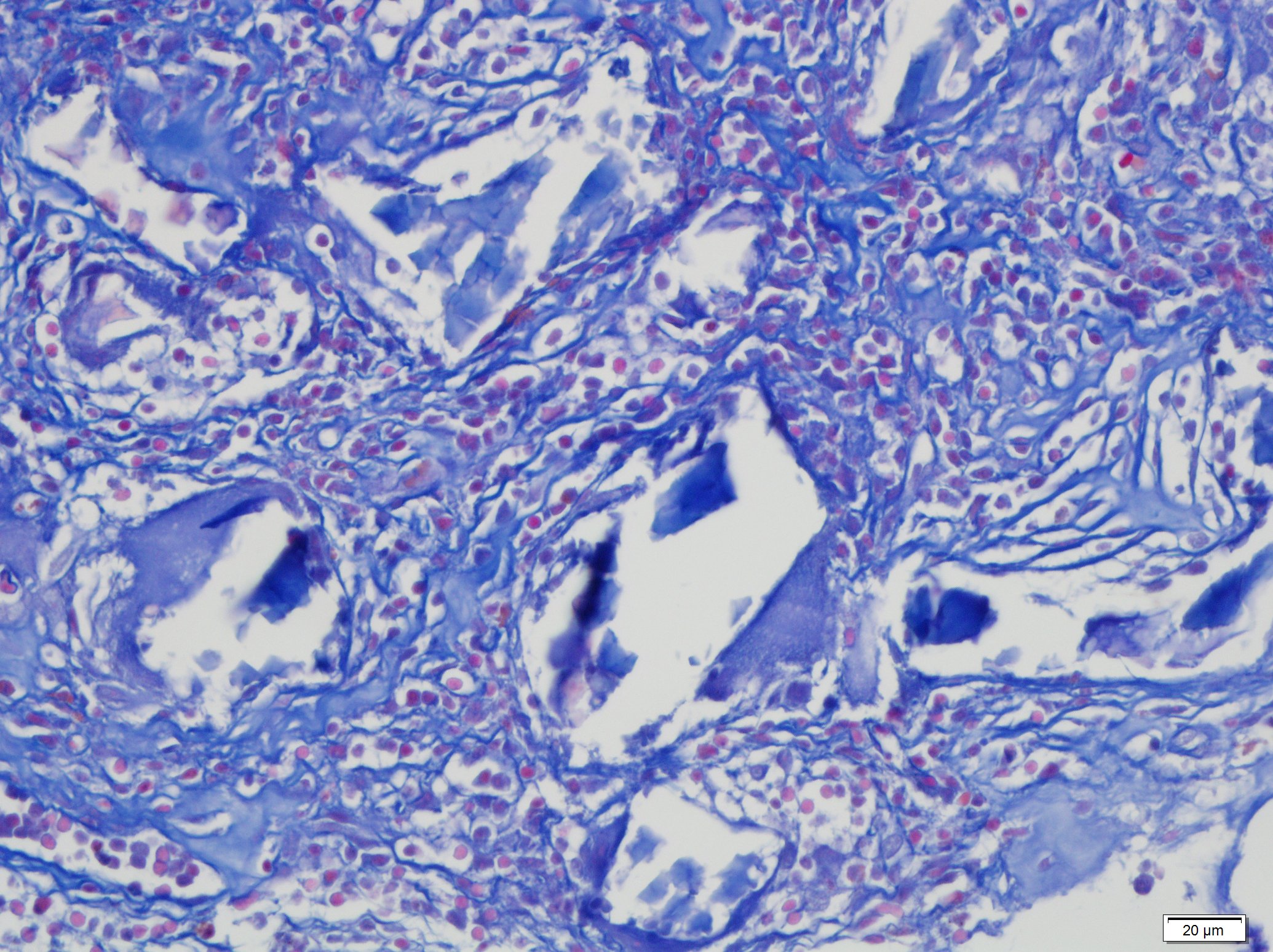

Supplement: S3 File — (ZIP) [file pone.0215499.s003.zip › masson's trichrome/4 weeks/4-7 40x-3.jpg]

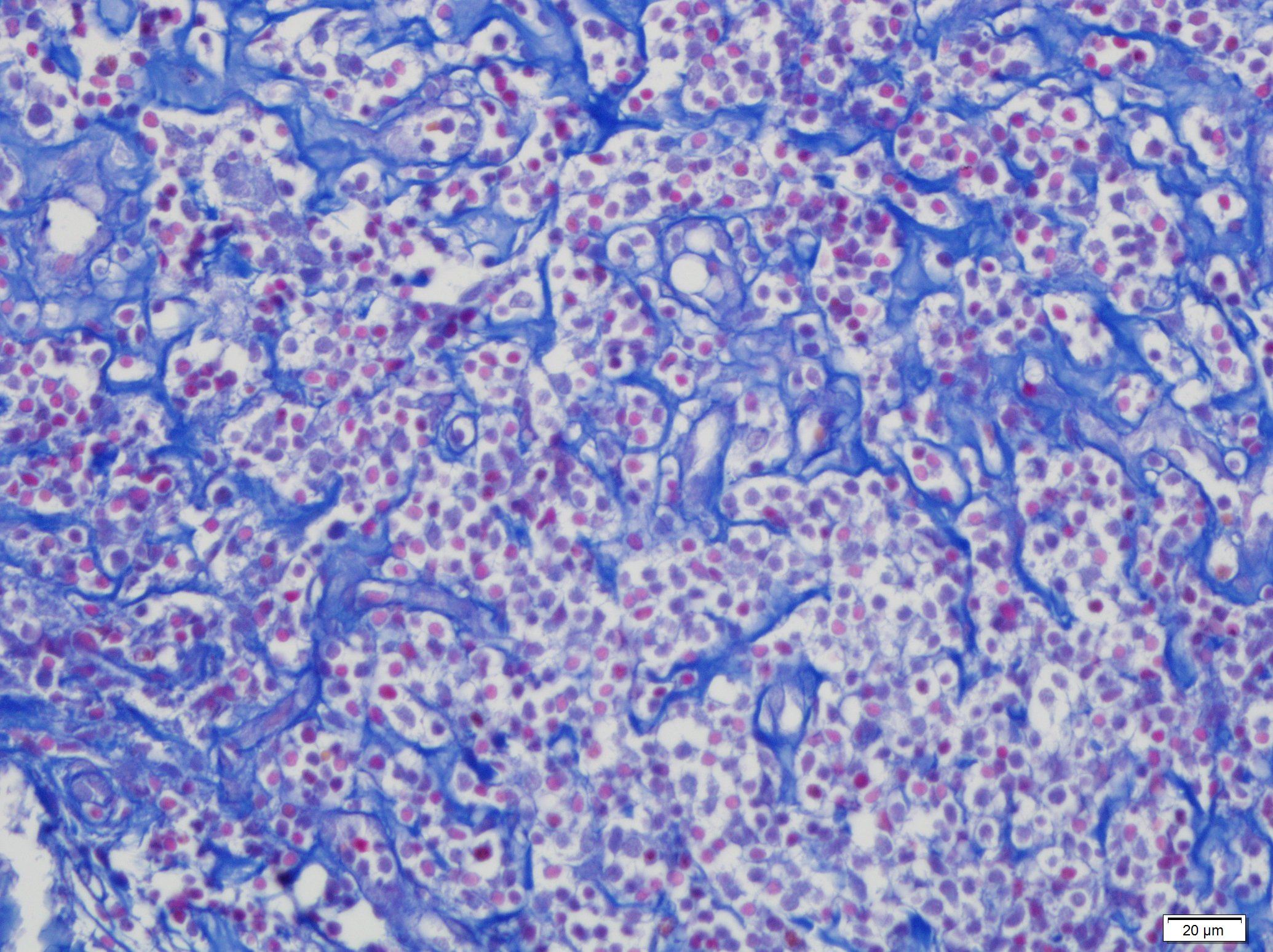

Supplement: S3 File — (ZIP) [file pone.0215499.s003.zip › masson's trichrome/4 weeks/4-7 40x-4.jpg]

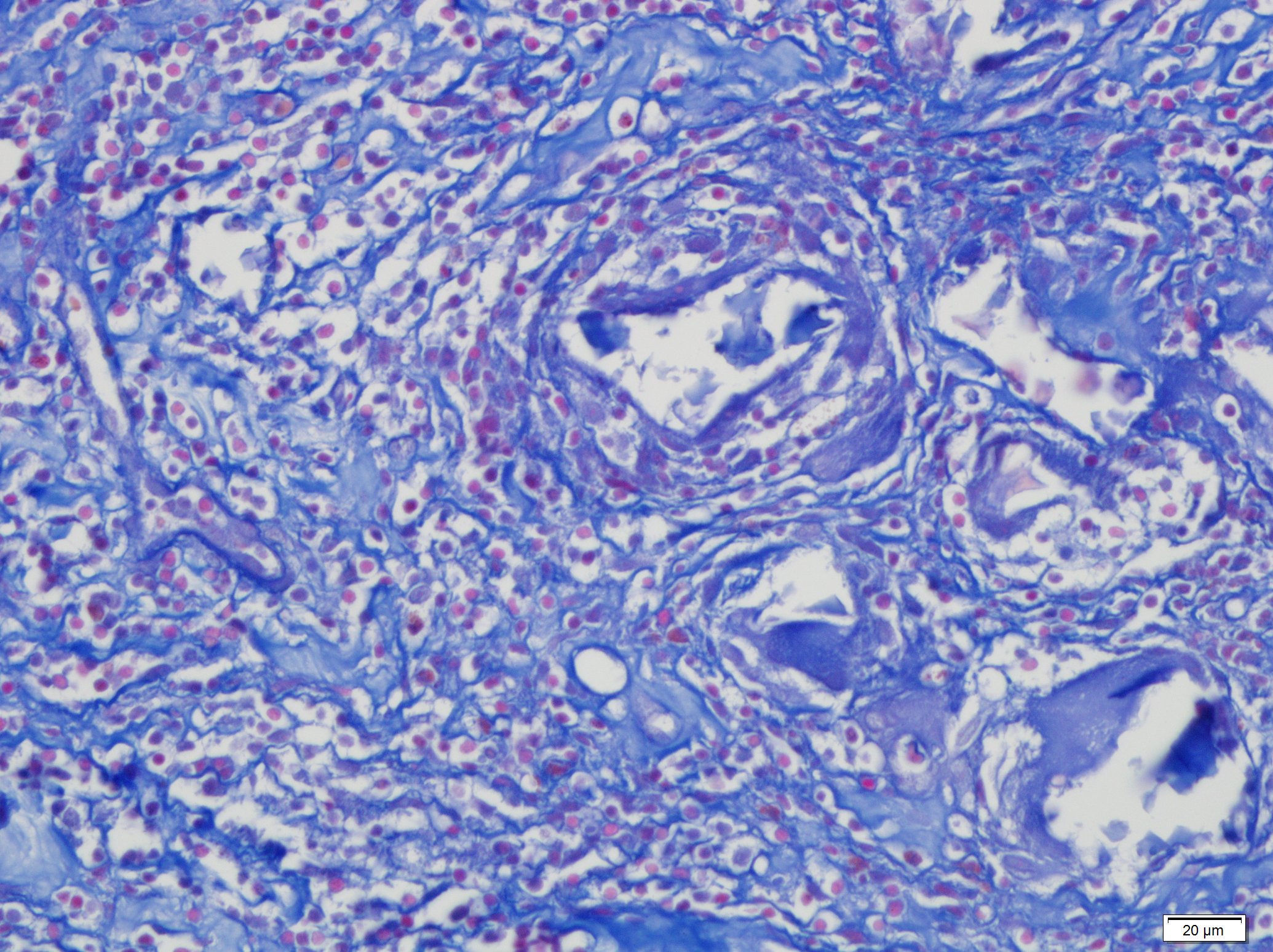

Supplement: S3 File — (ZIP) [file pone.0215499.s003.zip › masson's trichrome/4 weeks/4-7 40x.jpg]
